# A general mixture loss function to optimize a personalized predictive model

**DOI:** 10.1177/09622802261488055

**Published:** 2026-09-14

**Authors:** Tatiana Krikella, Joel A Dubin

**Affiliations:** 1 Department Mathematics & Statistics, 7991 York University, Toronto, Canada; 2 Department of Statistics and Actuarial Science, 8430 University of Waterloo, Waterloo, Canada

**Keywords:** Calibration, discrimination, mixture loss function, precision medicine, prediction model, subpopulation

## Abstract

Advances in precision medicine increasingly drive methodological innovation in health research. A key development is the use of personalized prediction models (PPMs) for a specific index patient, which are fit using a subpopulation similar to that index patient based on their covariates, and have been shown to outperform one-size-fits-all models, particularly in terms of model discrimination performance. We propose a generalized loss function that enables tuning of the subpopulation size used to fit a PPM. This loss function allows joint optimization of discrimination and calibration, allowing both the performance measures and their relative weights to be specified by the user. To reduce computational burden, we conducted extensive simulation studies to identify practical bounds for the grid of subpopulation sizes. Based on these results, we recommend using a lower bound of 20% and an upper bound of 70% of the entire training dataset. We apply the proposed method to both simulated and real-world datasets and demonstrate that previously observed relationships between subpopulation size and model performance are robust. Furthermore, we show that the choice of performance measures in the loss function influences the optimal subpopulation size selected. These findings support the flexible and computationally efficient implementation of PPMs in precision health research.

## 1. Introduction

Personalized predictive models (PPMs) are models which make predictions for a patient of interest, whom we call the index patient, using a model developed on a subpopulation of patients similar to that index patient. It has been shown that PPMs can lead to better prediction for an index patient compared to one-size-fits-all models which instead use the entire available population as training data.^1–3^ When fitting a PPM, one needs to decide how to define similarity, how many patients to include in the similar subpopulation, and how to measure model performance. It has been said that when taking these PPMs into practice, finding the optimal size of similar subpopulations will be important to ensure the best performing models, especially in a health care setting.^4^ Focusing on a binary response prediction problem and motivated by the fact that it has been criticized that calibration is not assessed nearly as often as discrimination,^5^ in previous work^2^ we proposed an algorithm to fit a PPM which jointly optimizes discrimination and calibration. *Discrimination* is the ability of the predictive model to distinguish between outcome classes, while *calibration* is the level of agreement between the observed outcomes and the predictions.

In our published algorithm, the size of subpopulation is tuned to minimize a loss function, which is an extension of a decomposition of the Brier Score, an overall performance measure, that consists of terms related to both discrimination and calibration. Specifically, the measure of calibration is related to Spiegelhalter’s *z*-statistic, and the measure of discrimination measures the lack of spread in the predictions.^6^ Both of these terms have an expected value of 0 under perfect performance, and we include a mixture term α∈[0,1] with the purpose of allowing the emphasis of one performance measure over another based on the needs of the researcher.

Van Calster et al.^7^ propose a calibration hierarchy consisting of four increasingly strict levels of calibration: mean, weak, moderate and strong. They say that although strong calibration is the utopic goal, it is unrealistic in practice, thus we should strive for models that are moderately calibrated. Many calibration measures reported in the literature do not reach the level of moderate calibration. Thus, we want to use a moderate measure of calibration when possible, such as the Integrated Calibration Index.^8^

Many algorithms proposed to fit PPMs have been based on optimizing the size of subpopulation using a popular measure of discrimination, the area under the receiver operating characteristic curve (AUROC).^3,9–11^ The relationship between the size of subpopulation, M , and the AUROC has thus been earlier investigated and, in Krikella and Dubin,^2^ we confirmed the results of Lee et al.^3^ and demonstrated that the relationship between the AUROC and M is negatively proportional, aside from the lowest values of M . The intuition behind this relationship is that when all patients available in the training data are used to fit a model to an index patient, the heterogeneity in patients makes it harder to distinguish between outcome classes. Although we hypothesize that this holds true under any dataset, we do not know whether this relationship is specific to the data considered: in Lee et al.,^3^ only one dataset was used, and in Krikella and Dubin,^2^ we only considered one simulated dataset and one dataset from the eICU Collaborative Research (intensive care unit patient) database.^12^ Further, we showed that although the best calibrated models use the entire training data, a smaller M may be the better choice that still results in a well-calibrated PPM. In other words, we showed that the relationship between M and all levels of calibration considered is quadratic, which is harder to understand intuitively. We want to further explore this relationship between M and calibration to determine whether this is much more generally the case.

We wish to address some important limitations of our earlier algorithm.^2^ Specifically, we introduce an updated loss function to tune M that is more generalized and allows the user to choose the measures of discrimination and calibration in the function. We also test our algorithm under a variety of datasets to further investigate the relationship between M and a number of performance measures, the results of which we then use to define lower and upper bounds on the grid of values on which to tune M to reduce the computational burden of the algorithm.

This article is organized as follows. We propose the updated loss function, along with an updated algorithm, in Section 2. This algorithm is then tested on 12 simulated datasets to determine the robustness of the relationship between M and various performance measures, the design of which and results are included in Section 3. Based on these results, we also provide recommendations for obtaining lower and upper bounds for the grid of values on which to tune M . In Section 4, our proposed updated algorithm is tested on data from the eICU database^12^ using two different loss functions, each differing in the measure of discrimination chosen. We conclude with a discussion of this proposed work in Section 5.

## 2. Methods

### 2.1. Notation

Let there be $n_{\mathrm{train}}$ patients in the training data, where each patient k is represented by a Euclidean vector, $X_{k}$ , k = 1,…, $n_{\mathrm{train}}$ , in the multi-dimension predictor space defined by p predictors. We let $x_{h}$ , h=1,…,p , represent the $n_{\mathrm{train}}\times 1$ vector consisting of each patient’s value of a given predictor. Further, let $X_{I}$ , $I=1,\ldots ,n_{\mathrm{test}}$ be the Euclidean vector representation of an index patient, I , in the test data for whom we would like to make a prediction.

The focus of the prediction task in this article is on binary classification. Thus, we consider a binary outcome. The actual outcome for an index patient I is represented by $y_{I}\in \{0,1\}$ . Let M be a scalar representing the size of the subpopulation in the training data consisting of similar patients to the index patient. Finally, let $p_{I}^{\left(M\right)}\in [0,1]$ represent the predicted probability of the outcome for a patient I found using a model fit on a similar subpopulation of size M .

### 2.2. Patient similarity metric

Prior to fitting a PPM, one must decide on a patient similarity metric to use. That is, a criterion that will be used to measure the similarity between individuals. In general, we label this similarity metric as $d(X_{I},X_{k})$ , which represents the similarity between an index patient I and a patient k in the training set. In our work, we focus on an unsupervised process, and ideally use subject-matter specific covariates that will be used both for the similarity metric calculation and in the prediction models. In our context, the unsupervised method is appropriate, since, in some settings, such as a new patient I being admitted to a hospital, we will not have an outcome label.

We use the same patient similarity metric as was used in Krikella and Dubin^2^: the *cosine similarity metric*. This was chosen as it is bounded, interpretable and able to handle non-continuous data. For more details, please see Krikella and Dubin.^2^

We note that the covariates used to determine the similarity between patients are the same which are used to fit the PPM.

### 2.3. Performance measures

To measure the performance of a PPM, one should measure both the discrimination and calibration of the model.^13^ In this study, to measure discrimination we use the area under the receiver operating characteristic curve (AUROC), the area under the precision recall curve (AUPRC), and the *lack of spread* in predictions.

The AUROC is a common measure of discrimination, however, in cases where the outcome we wish to predict is fairly rare, that is, has a low prevalence, AUPRC is a better measure of discrimination.^14,15^ The precision recall curve plots the trade-off between precision, which is true positive/(true positive + false positive) , often referred to as the *positive predictive value* (or PPV), and sensitivity, for all possible thresholds. The AUPRC has a value between 0 and 1, but unlike the AUROC which will be 0.5 for an uninformative model, the AUPRC of an uninformative model is dependent on the prevalence, and approaches 0 as the prevalence decreases.

The lack of spread in predictions,^6^ although not used often in isolation in practice, is the measure corresponding to discrimination in the Brier Score decomposition discussed by Rufibach.^16^ This term is 0 when the discrimination is perfect (i.e. all predictions are either 1 or 0), and under the worst case scenario of discrimination (i.e. all predictions determined are 0.5), the value of this term is 0.25.

We consider measures from the three increasingly strict levels of calibration proposed by Van Calster et al.^7^ These measures, in order of strength, include: the calibration-in-the-large (CITL), the calibration slope, and the integrated calibration index (ICI).^13^ These measures are identical to those used as measure of calibration in our previous work. For more details on these measures, please see Krikella and Dubin.^2^

Rufibach^16^ has introduced a two-term decomposition of the Brier Score into its calibration and discrimination subparts, shown in equation (1) below. Here, $n_{\mathrm{test}}$ denotes the size of the testing set (i.e. the number of index patients), $y_{I}$ corresponds to the observed binary outcome of the index patient, I , and ${\hat{p_{I}}}^{\left(M\right)}$ corresponds to the predicted probability for individual I experiencing the event, obtained from fitting a PPM on a similar subpopulation of size M .

(1) $$\frac{1}{n_{\mathrm{test}}}\sum_{I=1}^{n_{\mathrm{test}}}(y_{I}-{\hat{p_{I}}}^{\left(M\right)}{)}^{2}=\frac{1}{n_{\mathrm{test}}}\sum_{I=1}^{n_{\mathrm{test}}}[(y_{I}-{\hat{p_{I}}}^{\left(M\right)})(1-2{\hat{p_{I}}}^{y(M)})]+\frac{1}{n_{\mathrm{test}}}\sum_{I=1}^{n_{\mathrm{test}}}[{\hat{p_{I}}}^{\left(M\right)}(1-{\hat{p_{I}}}^{\left(M\right)})]$$

The first term after the equal sign measures the lack of calibration, with an expectation of 0 under perfect calibration. Specifically, if we define this calibration term in terms of the calibration hierarchy, since it measures the overall agreement between the predicted probabilities to the actual outcomes, this term is actually a measure of mean calibration, the weakest measure. The second term after the equal sign, as mentioned above, is a measure of discrimination, specifically, it is the lack of spread of the predictions.

Van Calster et al.^7^ recommend that when fitting clinical predictions models, we strive for moderate calibration, however the Brier Score assesses calibration through mean calibration (and discrimination through lack of spread). Further, it has been shown that the Brier Score may be lower for a model that is miscalibrated with good discrimination compared to a model that is better calibrated with average discrimination^17^ (note that a lower Brier Score indicates better performance). These facts support that we should be assessing discrimination and calibration separately to obtain a better representation of a model’s performance.

### 2.4. Tuning the size of subpopulation

When fitting a PPM, Lee^4^ suggests that choosing the optimal size of subpopulation, M , will become important when taking these methods into practice. Motivated by this idea, we introduced an algorithm^2^ that tunes M to find the value that jointly optimizes discrimination and mean calibration using a loss function, shown in equation (2) that is inspired by Rufibach’s^16^ two-term decomposition of the Brier Score:

(2) $$L^{\left(M\right)}=\frac{\alpha }{n_{\mathrm{test}}}\sum_{I=1}^{n_{\mathrm{test}}}(y_{I}-{\hat{p_{I}}}^{\left(M\right)})(1-2{\hat{p_{I}}}^{\left(M\right)})+\frac{1-\alpha }{n_{\mathrm{test}}}\sum_{I=1}^{n_{\mathrm{test}}}{\hat{p_{I}}}^{\left(M\right)}(1-{\hat{p_{I}}}^{\left(M\right)}).$$

The difference between our loss function in equation (2) and the Brier Score decomposition in equation (1) is that we have included a mixture term, α , which allows the emphasis of one performance measure over the other, if desired. As we increase α in equation (2), more emphasis is put on the mean calibration term in the loss function. Using this loss function is acceptable, especially if mean, weak and moderate calibration will always result in the same optimal M value. However, because we do not know this for sure, we propose another method of tuning the size of subpopulation. We present a more general loss function that allows the user to choose their preferred measures of calibration and discrimination, represented by C and D , respectively. This general loss function is shown in equation (3), and note that we keep the mixture term α∈[0,1] , which allows flexibility in weighting one performance measure over another. Alternatively, a researcher can decide to tune α . In this study, we do not tune α explicitly, however, we will show in both the simulation study and data analysis how changing the value of α has an effect on the optimal size of subpopulation, M .

(3) $$L^{\left(M\right)}=\frac{\alpha }{n_{\mathrm{test}}}\sum_{I=1}^{n_{\mathrm{test}}}C_{k}^{\left(M\right)}+\frac{1-\alpha }{n_{\mathrm{test}}}\sum_{I=1}^{n_{\mathrm{test}}}D_{k}^{\left(M\right)}$$

In the loss function shown in equation (3), we can, for example, use the ICI as the calibration measure, and lack of spread as the discrimination measure, to obtain the loss function shown in equation (4), which optimizes M in terms of discrimination and moderate calibration. In equation (4), ${\tilde{p_{I}}}^{\left(M\right)}$ are the smoothed predicted probabilities based on the LOESS calibration curve:

(4) $$L^{*(M)}=\frac{\alpha }{n_{\mathrm{test}}}\sum_{I=1}^{n_{\mathrm{test}}}\left|{\tilde{p_{I}}}^{\left(M\right)}-{\hat{p_{I}}}^{\left(M\right)}\right|+\frac{1-\alpha }{n_{\mathrm{test}}}\sum_{I=1}^{n_{\mathrm{test}}}{\hat{p_{I}}}^{\left(M\right)}(1-{\hat{p_{I}}}^{\left(M\right)}).$$

The original iteration of this algorithm^2^ also suffered from a large computational burden. One way of addressing this computational burden that we propose in this article is to determine a lower and upper bound on the grid of values used to tune M . Specifying a lower bound would also rectify the limitation of the algorithm where an optimal M could be chosen in the training/testing step that is not a sufficient sample size in the validation step to obtain stable predictions.

To determine appropriate bounds for M , we must assess the robustness of its relationship with the performance measures described in Krikella and Dubin.^2^ This ensures that the selected bounds preserve both discrimination and calibration quality. Prior findings indicate that lower values of M yield good discrimination, while the relationship between M and all three levels of calibration is generally quadratic. If these trends are robust across datasets with varying characteristics, as evaluated in Section 3, we will choose the lower bound to ensure sufficient sample size, and the upper bound to balance improved calibration without substantial loss in discrimination. The recommended bounds, in the form of proportions, are formally introduced following the simulation results in Section 3.2.

An updated algorithm to determine the optimal size of subpopulation to use when fitting PPMs is proposed below. This algorithm consists of six steps, with the bulk of the work occurring in Steps 3 and 4. Step 3 is the tuning step, where the optimal M is determined by fitting a PPM for each index patient in the testing set. We loop through a grid of M values to determine the similar subpopulation to which the PPM is fit for each index patient, then the optimal M is the one which results in the lowest value of the specified loss function (averaged over repeated cross-validation folds, with further details below). In Step 4, the optimal M value is validated using a bootstrap approach. We note that, when using this algorithm in practice, the tuning and validation step only need to occur once on a desired training set to obtain an optimal M value that can be used for any new patient. We discuss the idea of a unique optimal M for each index patient in Section 5. If an optimal M has already been determined using this algorithm (on training data from a population that is similar to that of the index patient), then fitting a PPM simply involves determining the subpopulation similar to the index patient using that optimal M , and fitting the desired model (e.g. logistic regression, random forest) to that subpopulation to ultimately obtain a prediction.

Split the entire population into a (100q) % hold-out validation set ( $n_{\mathrm{val}}$ ) and (100[1−q]) % training–testing (TrTe) set ( $n_{\mathrm{TrTe}}$ ), where q∈[0,1] . We often use q=0.2 .
Determine a measure of discrimination and a measure of calibration to be included in the generalized loss function, $L^{\left(M\right)}$ , displayed in equation (3), along with a desired α value.
Tune M by following the steps below:
  - Randomly split the TrTe set into distinct training and testing sets using K -fold cross-validation. The testing set contains $n_{\mathrm{test}}$ index patients for whom we wish to determine a prediction using a similar subpopulation of the training set.
    i. For index patient I in the test set, calculate $d(X_{I},X_{k})$ , for $k=1,\ldots ,n_{\mathrm{train}}$ in the training set, where $d(X_{I},X_{k})$ is the similarity metric between the index patient and the $k^{th}$ training set patient.
    ii. Using our recommendations for an upper and lower bound described in Section 3.2, define a grid of M values and tune M through random grid search.
      A. Sort all $d(X_{I},X_{k})$ in descending order, and the patients corresponding to the top M scores form the subpopulation.
      B. Fit the predictive model of interest, such as logistic regression, random forest, and so on, on the subpopulation.
      C. Average the values of the specified loss function over the K -folds.
    iii. Repeat the K -fold cross validation v times, where vK≥200 .
    iv. The optimal M , called $M^{\mathrm{opt}}$ , is chosen as the one which minimizes the updated loss function over the v repeated K -folds.
    v.
$M^{opt}$ is divided by the number of patients in the training set to obtain the optimal M proportion, $M_{\mathrm{prop}}^{\mathrm{opt}}=M^{\mathrm{opt}}/n_{\mathrm{train}}$ .
4. Draw B bootstrap samples from the validation sample (each the same size as the validation sample). Do the following steps for each bootstrap sample:
  - Randomly split the bootstrap sample into an 80% training sample and 20% testing sample.
  - Set the integer $M=\mathrm{ceiling}(n_{\mathrm{val}.\mathrm{train}}M_{\mathrm{prop}}^{\mathrm{opt}})$ where $n_{\mathrm{val}.\mathrm{train}}$ is the size of the training set.
  - For index patient I in the test set, calculate $d(X_{I},X_{k})$ , for $k=1,\ldots ,n_{\mathrm{val}.\mathrm{train}}$ in the training set, where $d(X_{I},X_{k})$ is the similarity metric between the index patient and the $k^{th}$ patient.
  - Use the M obtained in step 4(b) to form the subpopulation.
  - Fit the model of interest on the subpopulation.
  - Measure the performance of the model using, for example, the discrimination and calibration measures discussed in Section 2.3.
5. To quantify the uncertainty of the performance measures, fit bias-corrected and accelerated (BCa) bootstrap confidence intervals^18^ for each of the performance measures using the B bootstrap estimates.
6. Repeat Step 1-6 Z times, that is, repeat the process with Z different validation hold-out samples (and, thus, TrTe samples) to view consistency of the tuning of M (step (3) above) as well as the model performance measures in different hold-out validation samples. We suggest Z to be approximately ten.

Although seemingly very similar, there are two important distinctions between this algorithm and the one in Krikella and Dubin.^2^ Firstly, we updated the previous loss function to be more general, shown in equation (3), in the sense that we do not specify which measures of discrimination and calibration to be included in the function, they are user-specified. Thus, in this algorithm, we have included Step 2, which involves choosing measures of discrimination and calibration to include in the defined loss function. Secondly, this algorithm contains specific instructions on how to define the grid of values on which to tune M with the intention of lowering the computation time of running this algorithm. We specifically motivate and state our recommendations for these bounds in Section 3. Also, if the user wishes, α could be tuned as an additional step in the above algorithm.

## 3. Simulation studies

We conducted two sets of simulation studies using R version 4.4.1.^19^ The first involves investigating the relationship between the size of subpopulation and six different performance measures under various outcome models. The goal of this simulation study was to determine if the relationships found in Krikella and Dubin^2^ are robust against various true underlying outcome models. In the second simulation study, we demonstrate our previously published algorithm but with bounds put on the grid of values on which M is tuned to lessen the computational burden, and using the updated loss function with the lack of spread as the discrimination measure and the ICI as the calibration measure.

### 3.1. Setup

We describe the data generation, methods, and performance measures relevant to the simulation studies conducted.

We considered a total sample size of n=10,000 and 40 predictors.

We replicated a scenario in which we have two clusters of patients of size $n_{1}=5000$ and $n_{2}=5000$ , each cluster differing in the distributions of their respective predictors, as well as the strength of their association to the binary outcome of interest. Both patient clusters have the same predictors relating to the outcome, but the coefficients may differ. In the first cluster of patients, of the 40 predictors generated, 20 predictors were continuous with eight predictors simulated from a N(30,4) distribution, five predictors simulated from a N(5,1) distribution and seven predictors simulated from a N(89,29) distribution. We then simulated seven predictors from a Bernoulli(0.3) distribution, nine predictors from a Bernoulli(0.5) distribution and four predictors from a Bernoulli(0.7) distribution. In the second cluster of patients, 20 predictors were continuous with eight predictors simulated from a N(20,8) distribution, five predictors simulated from a N(15,8) distribution and seven predictors simulated from a N(50,9) distribution. We then simulated seven predictors from a Bernoulli(0.5) distribution, nine predictors from a Bernoulli(0.15) distribution and four predictors from a Bernoulli(0.45) distribution. These two clusters of patients were included in one dataset of size $n_{1}+n_{2}=n=10000$ . We standardized each predictor within this dataset so that all values of each predictor were between − 1 and 1. This specific standardization was employed to be naturally incorporated into the cosine similarity metric calculation.

We defined 12 different true outcome models using the outline described above. We simulated six outcomes from a linear model which differed by the number of predictors associated with the outcome, which could be either 10/40 or 20/40, or by prevalence of the outcome, which were low( ∼ 0.05), moderate ( ∼ 0.15) or balanced ( ∼ 0.5). We also simulated six outcomes from a non-linear model which differed by the number of predictors associated with the outcome, or by prevalence, using the same specifications described above.

We provide examples of two of these outcome models in equations (5) and (6). Specifically, equation (5) is a linear model which results in an outcome with low prevalence, and has ten predictors associated with the outcome, and equation (6) is a non-linear model which results in an outcome with moderate prevalence, and has ten predictors associated with the outcome. In this model, $z_{1}$ and $z_{2}$ are the predictors of the first and second cluster of patients, respectively. Each cluster of patients has the same predictors associated with the outcome, but these relationships generally differ in direction and/or strength of the association.

(5) $$\begin{array}{rl} z_{1} & =-3-3x_{1}+x_{2}+0.5x_{9}+x_{16}+x_{19}+x_{22}+x_{30}\\ & \;-x_{34}-4x_{38}-5x_{39}+\epsilon \\ z_{2} & =-3.5+3x_{1}+x_{2}-x_{9}+2x_{16}+x_{19}+x_{22}+4x_{30}\\ & \;+5x_{34}+3x_{38}+3x_{39}+\epsilon \end{array}$$

(6) $$\begin{array}{rl} z_{1} & =-2x_{1}x_{39}-4\exp\;(x_{6}x_{30})-2\sin\;(x_{9}x_{16})\\ & \;+0.01(x_{19}{)}^{3}+3x_{22}x_{34}+2x_{38}+\epsilon \\ z_{2} & =x_{1}x_{39}-\exp\;(x_{19}x_{38})-2\cos\;(x_{9}x_{16})\\ & \;+0.01(x_{6}{)}^{2}-3x_{22}x_{34}+3x_{30}+\epsilon \end{array}$$

After the outcome was simulated from one of the 12 different models, both $z_{i}$ , i=1,2 were passed through an inverse logit function to obtain two probabilities, $\pi_{i}$ , i=1,2 , as shown in equation (7). These probabilities were then used to simulate Bernoulli outcomes, $y_{ki}$ , where $Y_{ki}∼Bernoulli(\pi_{i})$ .

(7) $$\pi_{i}=\frac{1}{\left(1+\exp\;(-z_{i})\right)}.$$

The final dataset includes the 40 predictors and the binary outcome of all n=10,000 patients. We stress again that, as we considered 12 different outcome models, we ultimately simulated 12 different scenarios.

We conduct two different simulation studies in this section using the scenarios described above. In the first, we are simply investigating the relationship between M and the various performance measures, so we do not split the data into a TrTe set and a validation set. We simply perform 10-fold cross validation to internally validate the performance, resulting in a training set of size $n_{\mathrm{train}}=9000$ , and a testing set of size $n_{\mathrm{test}}=1000$ . Recall that the testing set consists of the index patients for whom we wish to make a prediction. In the second simulation study, we demonstrate how to tune M , and here we do first split the dataset into an 20% hold-out validation set ( $n_{\mathrm{val}}=2000$ ), and an 80% training-testing (TrTe) set ( $n_{\mathrm{TrTe}}=8000$ ). The TrTe set is then further split via 10-fold cross validation ( $n_{\mathrm{train}}=7200$ , $n_{\mathrm{test}}=800$ ).

When fitting the PPMs, we used a logistic regression model. We comment on the use of other, more flexible non-likelihood based models in Section 5. When tuning the optimal size of subpopulation, M , we internally validated these models by conducted 20-repeated 10-fold cross validation. We do this for each of the 12 scenarios, and for each scenario, we simulate ten randomly generated datasets using ten different seeds to allow for the calculation of the Monte Carlo standard error (MCSE) for each performance measure described in Section 2.3. To calculate each performance measure, we obtain predictions for each index patient, the predictions are stored in a vector, and we compare this vector of predictions to the true outcomes. We only repeat each simulation scenario ten times due to the considerable computational expense of each simulation run. This is a point we further address in Section 5.

In the validation step, we calculate BCa bootstrap confidence intervals^18^ for each performance measure, where we simulate B=1000 bootstrap samples.

### 3.2. Relationships between the size of subpopulation and the performance measures

In this section, we display the results from two of 12 scenarios. Scenario A has a linear true outcome model with 20 predictors associated with the outcome and a resulting prevalence that is approximately 0.15. Scenario B has a non-linear true outcome model, also with 20 predictors associated with the outcome and a resulting prevalence that is lower, at approximately 0.05. Descriptions of all 12 scenarios are shown in Table 1, with the two specific scenarios described above in bold. Note that Scenario A corresponds to Scenario 5 in the table, and Scenario B corresponds to Scenario 10 in the table. Results from the remaining ten scenarios are found in Supplemental Material, Section S.1.

**Table 1.** Characteristics of the 12 simulation scenarios.

Scenario	Linearity of outcome	Number of predictors associated with outcome	Outcome prevalence
Scenario 1	Linear	10	0.05
Scenario 2	Linear	10	0.15
Scenario 3	Linear	10	0.50
Scenario 4	Linear	20	0.05
**Scenario 5**	**Linear**	**20**	**0.15**
Scenario 6	Linear	20	0.50
Scenario 7	Non-Linear	10	0.05
Scenario 8	Non-Linear	10	0.15
Scenario 9	Non-Linear	10	0.50
**Scenario 10**	**Non-Linear**	**20**	**0.05**
Scenario 11	Non-Linear	20	0.15
Scenario 12	Non-Linear	20	0.50

We analyze the relationships between M and six performance measures (AUROC, AUPRC, lack of spread, CITL, calibration slope and ICI) for Scenario A from Table 1, shown in Figure 1. The Monte Carlo simulation error (MCSE), calculated across ten simulated datasets for each performance measure and each value of M , is presented in the caption of Figure 1. The relationship between M and the AUROC and AUPRC, as seen in Figure 1(a) and (b), is negatively proportional after M=3000 , that is, the discrimination improves up to a point, where it then begins to deteriorate. Under the lack of spread measure, displayed in Figure 1(c), the relationship is negatively proportional, even for the lowest values of M . As M increases, the discrimination deteriorates, meaning that a low value of M will result in a PPM with relatively better discriminative ability than a higher value of M . Figure 1(d)–(f) displays the relationship between M and calibration using the three increasingly strict measures of calibration: the CITL, calibration slope, and the ICI. The calibration slope and the ICI both have a concave quadratic relationship with M , with both low and high values of M resulting in models with very good calibration. Under calibration slope, the best performing model is when M=1250 , which is the second best performing model under the ICI; the best, only by a difference of 0.003, being under M=9000 for the latter measure of calibration. The CITL has a convex relationship with M , and is minimized at M=4000 . We do note that the range of CITL values, which has differences in the thousandths, specifically 0.003, is much smaller compared to the calibration slope and the ICI, which have a ranges of 0.631 and 0.017, respectively.

**Figure 1.**
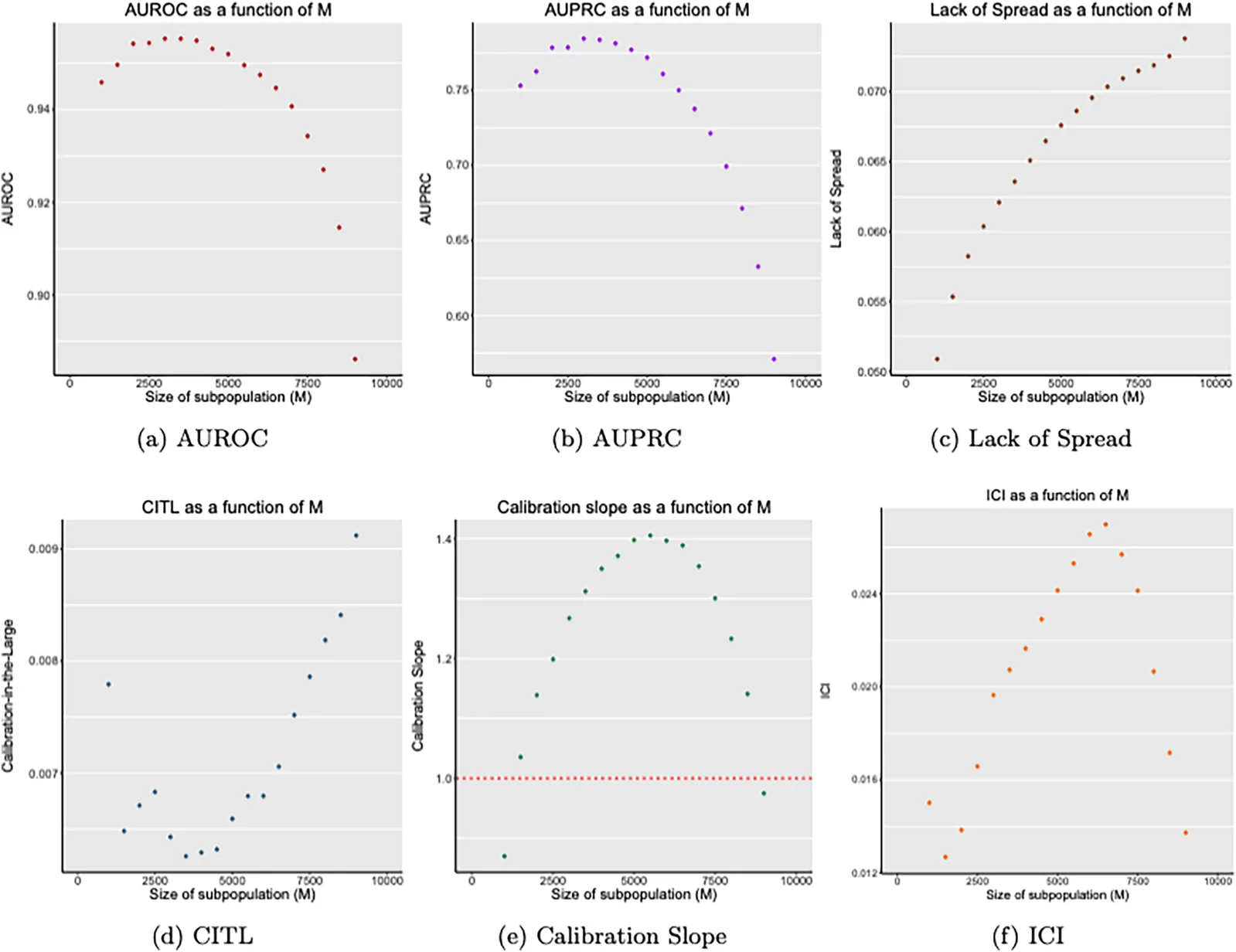
Performance measures as a function of *M* for Scenario A. The Monte Carlo simulation error (MCSE) across all values of *M* for the AUROC, AUPRC, Lack of Spread, CITL and the ICI was less than 0.0045. The MCSE for the calibration slope across all values of *M* was less than 0.015.

The relationship between M and the same six performance measures for Scenario B from Table 1 is shown in Figure 2, where the MCSE is also presented in the caption. As expected, the AUROC and the AUPRC improve up to a point, specifically at M=2500 , where they then begin to deteriorate as M increases. We note, however, that it might be argued that the AUROC is not the best measure of discrimination to consider under an outcome proportion of near 0.05. The relationship between lack of spread and M , shown in Figure 2(c), is also generally negatively proportional, however, at M=3500 , the discrimination under this measure begins to improve slightly before deteriorating again. Also, there is an outlier at M=9000 that indicates improvement in the lack of spread from the previous M value of M=8500 . We would like to note that out of the 12 scenarios considered, this case is the only one which did not result in a true negative proportional relationship between M and the lack of spread. The complexity of the outcome model (i.e. non-linear, low prevalence and 20 predictors associated with the outcome) may have had an effect on this relationship.

**Figure 2.**
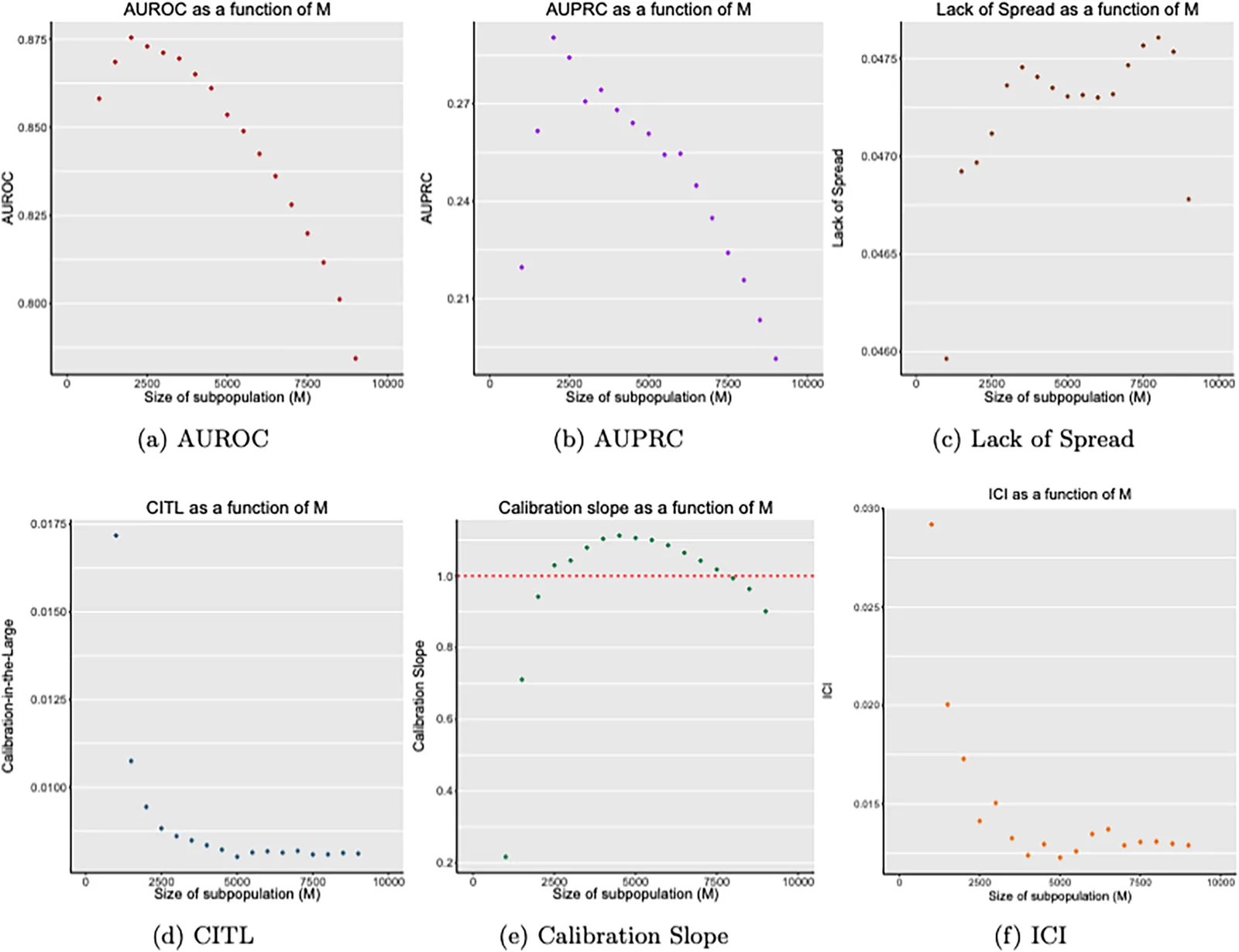
Performance measures as a function of *M* for Scenario B. The Monte Carlo simulation error (MCSE) across all values of *M* for the AUROC, CITL and the ICI was less than 0.0045. The MCSE for the AUPRC and the lack of spread across all values of *M* was less than 0.01. The MCSE for the calibration slope across all values of *M* was less than 0.04.

Both the CITL and the ICI, as shown in Figure 2(d) and (f), are optimal under relatively larger values of M , the former being minimized at M=7250 and the latter at M=6500 . The relationship between M and the calibration slope is, once again, concave, seen in Figure 2(e), and is optimal, or a negligible distance away from optimal, at both M=2250 and M=8500 . Similar to what was seen in the previous case, the second best choice of M under the ICI is the same value which optimizes the calibration slope: M=2250 . All three measures are in agreement in terms of the worst calibrated model, which in the case of this scenario, is the model which is fit on the smallest size of subpopulation considered: M=1000 . The difference between the best calibrated PPM and worst calibrated PPM, in terms of the CITL, is 0.014, which is a much wider range compared to the range of CITL values in the previous scenario. The calibration slope also has a much wider range compared to what was seen in Figure 1, where here, the difference between the best and worst calibrated model is 0.989. The ICI has a similar range to the previous example, valued at 0.016. In this simulation study, higher values of M perform better than lower values of M for all three levels of calibration, however, a lower value of M still results in a model with good weak and moderate calibration.

We would like to point out that the relationships observed in the above two cases are not unique to the specific characteristics of the scenarios considered. For instance, Scenario B has an outcome that is generated from a non-linear model, and in Figure 2, we see that the optimal model under the ICI is one which is trained on approximately 65% of the training dataset. However, there were other scenarios we considered, shown in Supplemental Material Section S.1, which had outcomes generated from non-linear models and resulted in a low optimal M proportion under the ICI. We cannot conclude that any of these results are due to the specific characteristics we varied, however, we can make some overall conclusions that we discuss below.

Under all 12 simulation scenarios, the relationship between M and discrimination was negatively proportional, aside from the lowest values of M when using the AUROC or the AUPRC as measure of performance. In terms of calibration, the relationships between M and calibration differed under different measures, as well as under different scenarios. In many cases, a low M was good, if not the best, under the ICI. The calibration slope, in most cases, had a concave relationship with M , resulting in relatively better weak calibration in models fit on both small and large M values. Due to this, there was usually agreement between the optimal M under the calibration slope and the ICI. The optimal M under the CITL was more sensitive to changes in characteristics, with no clear pattern among the 12 cases, but in many cases, it was not in agreement with the optimal M value found under the slope or ICI. As measures of moderate calibration ensure that models have good mean, weak and moderate calibration, we emphasize the recommendation by Van Calster et al.^7^ and state that, in the general loss function shown in equation (3), although there is flexibility in which calibration measure to use, we encourage the use of a measure of moderate calibration, such as the ICI.

To investigate the effect of sample size on these relationships we conducted a small simulation study using Scenario A where we considering a sample size of N=20,000 and N=5,000 . We found that changing the sample size did not drastically change the manner of the relationship between M and discrimination or calibration. We have included the results of this small simulation study in Supplemental Material Section S.2.

Based on these results, we recommend that when tuning M , the lower bound on the grid of values should be $0.2n_{\mathrm{train}}$ , where $n_{\mathrm{train}}$ is the size of the training set (in this case, $n_{\mathrm{train}}=0.9*n_{\mathrm{TrTe}}$ to account for the 10-fold cross validation), and the upper bound on the grid of values to be either $0.5n_{\mathrm{train}}$ or $0.7n_{\mathrm{train}}$ , depending on the interests of the study. We recommend the lower bound be 20% of the training data as we see that this proportion gives at the very least, good, if not the best, discriminative ability under the AUROC and AUPRC while still ensuring that the sample size in the validation step results in stable model estimation. During the simulation study, there was empirical evidence of model instability when smaller proportions were used, which motivated the selection of this lower bound. In smaller datasets or settings with very low outcome prevalence, we recommend that researchers additionally consider the absolute number of outcome events to ensure stable model estimation and reliable performance assessment. The upper bound on this grid of values will depend on the α value chosen, specifically, how much of an interest there is in the calibration of the model. For α=0.5 or lower, thus if there is equal interest in both discrimination and calibration, or even a greater interest in discrimination, we suggest an upper bound of 50%, which greatly reduces the values on which to tune M while still capturing values which are known to include the optimal size under discrimination and, possibly, the optimal size under calibration as well. For larger values of α , that is, values of α greater than 0.5, when there is greater interest in calibration, we recommended an upper bound of 70% of the training data, which could capture the optimal value of M in cases where that value is large in terms of calibration. We do not recommend a value higher than 70% as an M value greater than this results in a significant deterioration in the discriminative ability of the model.

### 3.3. Relationship between the size of subpopulation and the mixture term

Now that we have a defined lower bound on the grid of values on which we tune M , we can consider values of α lower than 0.475 (i.e. the lowest value of α considered in Krikella and Dubin^2^) since the defined bound ensures there will be a sufficient sample size for the validation step. Thus, in this simulation study, we consider the following values of α : 0.5 , 0.5±0.1 , 0.5±0.25 , 0.5±0.4 . We choose 0.5 as the baseline since this weight will put equal value on both the discrimination and calibration terms in the loss function.

We begin by considering Scenario A from Table 1. Recall in Figure 1, a low value M is associated with good discrimination as well as good weak and moderate calibration, but a value of M that is approximately 50% of the whole training data leads to a model with good mean calibration. We applied our proposed algorithm from Section 2.4, and we defined the measure of calibration in the loss function to be the ICI and the measure of discrimination to be the lack of spread to, ultimately, obtain the loss function shown in equation (4). Figure 3(a) shows that the resulting optimal M under the seven values of α considered is low. This plot shows the relationship between M and α using five different randomly generated datasets, and is in line with the information displayed in Figure 1. In Figure 1, the optimal value of M , of one seed considered, under the ICI was M=9000 , but the difference between this optimal model and the second optimal model was very small, thus in this simulation study where we conducted 20 repeated 10-fold cross validation, across ten different seeds, the more robust result shows that the smaller value of M was, indeed, the optimal value. For all ten randomly generated datasets, the optimal M proportion is approximately 0.22 under all values of α , though in the plot we only display five out of ten seeds for ease of visualization.

**Figure 3.**
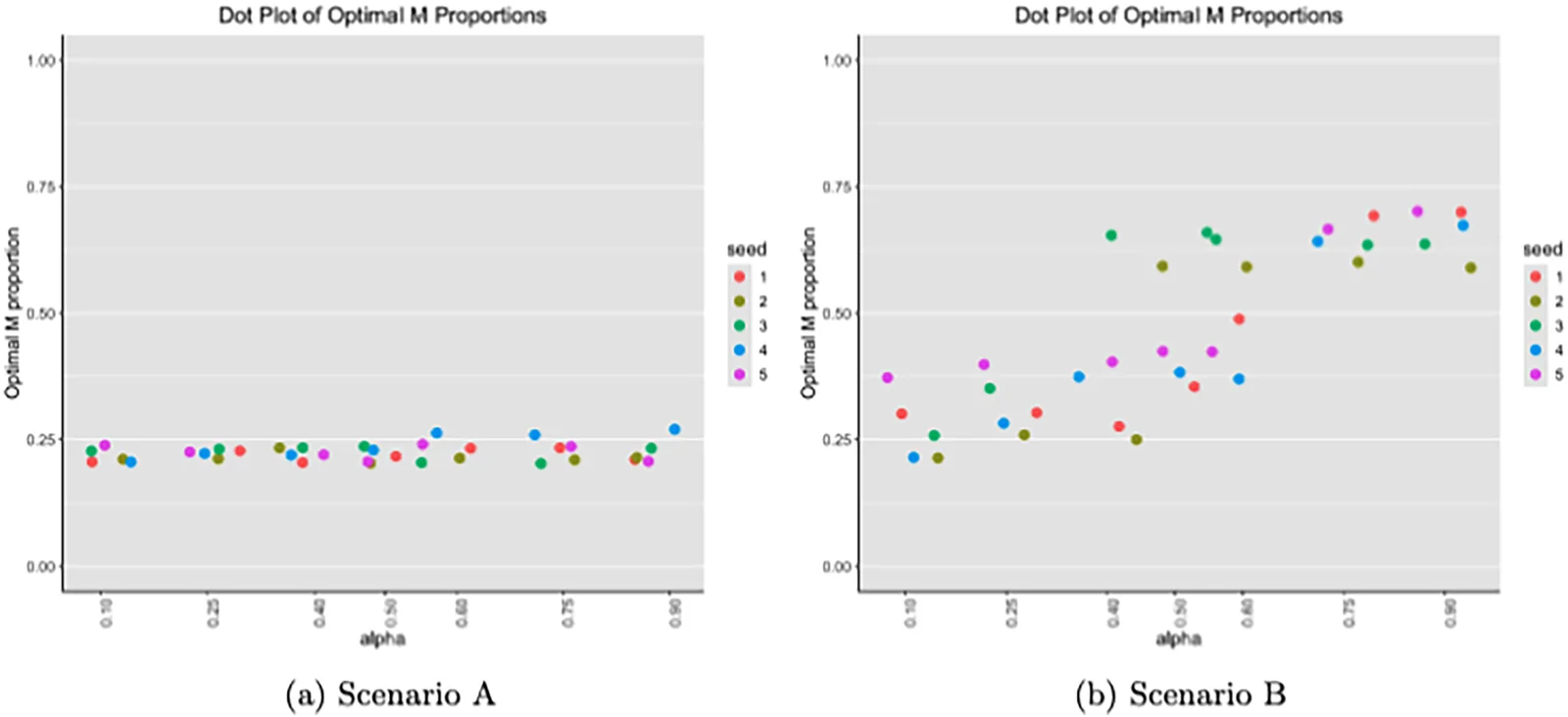
Performance measures as a function of M for Scenarios A and B.

Next, we considered Scenario B described in Table 1. As shown in Figure 2, high values of M are associated with better mean, weak, and moderate calibration, while low values of M are associated with better discrimination. In Figure 3(b), it is shown that as we increase α , the optimal M proportion also increases, under all ten randomly generated datasets, though only five out of ten are shown in this plot. For higher values of α , there is more emphasis on the moderate calibration term in our loss function, thus these results coincide with the results seen in Figure 2: a higher value of M is associated with good moderate calibration. For low values of α , the optimal M proportion is between 0.2 and 0.4. For high values of alpha, the optimal M proportion is between 0.6 and 0.7. Unlike in Figure 3(a), there is more variability in the optimal M proportions, especially for values of α that are in the middle of the range (i.e. 0.4, 0.5, 0.6). However, the results are still consistent with what we expected as there is still the increasing relationship between M and α , regardless of the outliers.

## 4. Data analysis

We applied our proposed algorithm to a dataset from the eICU Database^12^ to predict mortality of patients with diseases of the circulatory system.

This dataset only considers patients in their first ICU stay of their first hospital visit and had a disease of the circulatory system, identified by their ICD9 code. These patients have survived in the ICU for at least 24 hours, but their stay did not exceed 14 days. The minimum of 24 hours was chosen to ensure that we could have the necessary data obtained to calculate the useful APACHE (IVa) score, a useful severity of illness score for ICU patients, which is an improvement built upon APACHE IV.^20^ The maximum of 14 days was chosen as these patients were outliers, with only 2% of unique patients with a circulatory system disease having ICU stays lasting more than 14 days. However, putting these bounds still gave us sufficient sample size to demonstrate our proposed methodology on this dataset. Any patient with missing values in any of the predictors, as well as any patient with no vital sign data collected in the first 24 hours of their ICU stay, was removed. This approach is not recommended in practice, but dealing with missing data was not the focus of the current manuscript. Our primary purpose is to demonstrate the proposed methodology in a real application dataset. A visualization of the cohort selection from the eICU database is included in the Supplemental Material, Section S.3. In total, we have a cohort patient size of 23,928 patients. We used 80% of this cohort as the TrTe set, where we then performed 10-fold cross validation. Thus, the TrTe set consisted of 19,143 patients, and once the TrTe set was split into distinct training and testing sets using 10-fold cross validation, the training set consisted of 17,229 patients and the testing set consisted of 1,914 patients.

For each patient, the following predictors were extracted:

- Admission and demographic data: age, gender (male/female), and hospital discharge status (Expired/Alive).
- Vital signs from the first 24 hours after the start time of ICU admission: oxygen saturation (sao2), respiratory rate (respiration), and heart rate.
- APACHE IVa score: a severity-of-illness score utilizing demographic information as well as medical information collected within the first 24 hours of a patient’s ICU stay.

To identify the dominant modes of temporal variation of the vitals signs in the first 24-hours of a patient’s stay in the ICU, we used functional principal components (FPCs).^21^ We extracted the first three FPCs for each of the vital signs, which cumulatively explained at least 95% of the total variation in the trajectory of each given vital sign. Our resulting dataset thus consists of 13 predictors, including the first three FPC scores for each of the three vital signs.

When testing the algorithm, we considered α={0.1,0.25,0.4,0.5,0.6,0.75,0.9} . We conducted 20 repeated 10-fold CV in the training/testing step of the algorithm, then validated the results with a hold-out sample. We fit a logistic regression model to the data as the outcome (hospital discharge status) is binary (i.e. expired or alive). As the prevalence of outcome in this dataset is approximately 0.107, we use the AUPRC as a measure of discrimination instead of the AUROC. We also include a measure of the lack of spread, and the three measures of calibration used in Section 3.

We show the relationships between M and the performance measures when fitting PPMs to this dataset in Figure 4. As expected, Figure 4(a) shows that the AUPRC of the model increases up to a point, but instead of decreasing as M increases, the value of the measure stays relatively constant after this point, but still decreases slightly. The optimal M value under the AUPRC is M=9000 . Figure 4(b) shows the relationship between M and lack of spread, and, in general, as the size of subpopulation increases, the lack of spread of the model deteriorates, with the optimal M value being M=4500 . Figure 4(c) and (d) shows that the optimal M under mean and weak calibration is very high, however we do note that the differences in the CITL between models fit using varying values of M is very small. Further, similar to what was seen in the simulation study, there are two values of M that result in optimal models under the calibration slope, with the first optimal value being approximately 50% of the entire training data, and the second being nearly the entire dataset. Under the ICI, the optimal M is the smallest value considered, however, moderate calibration performs very well for high values of M as well, as seen in Figure 4(e).

**Figure 4.**
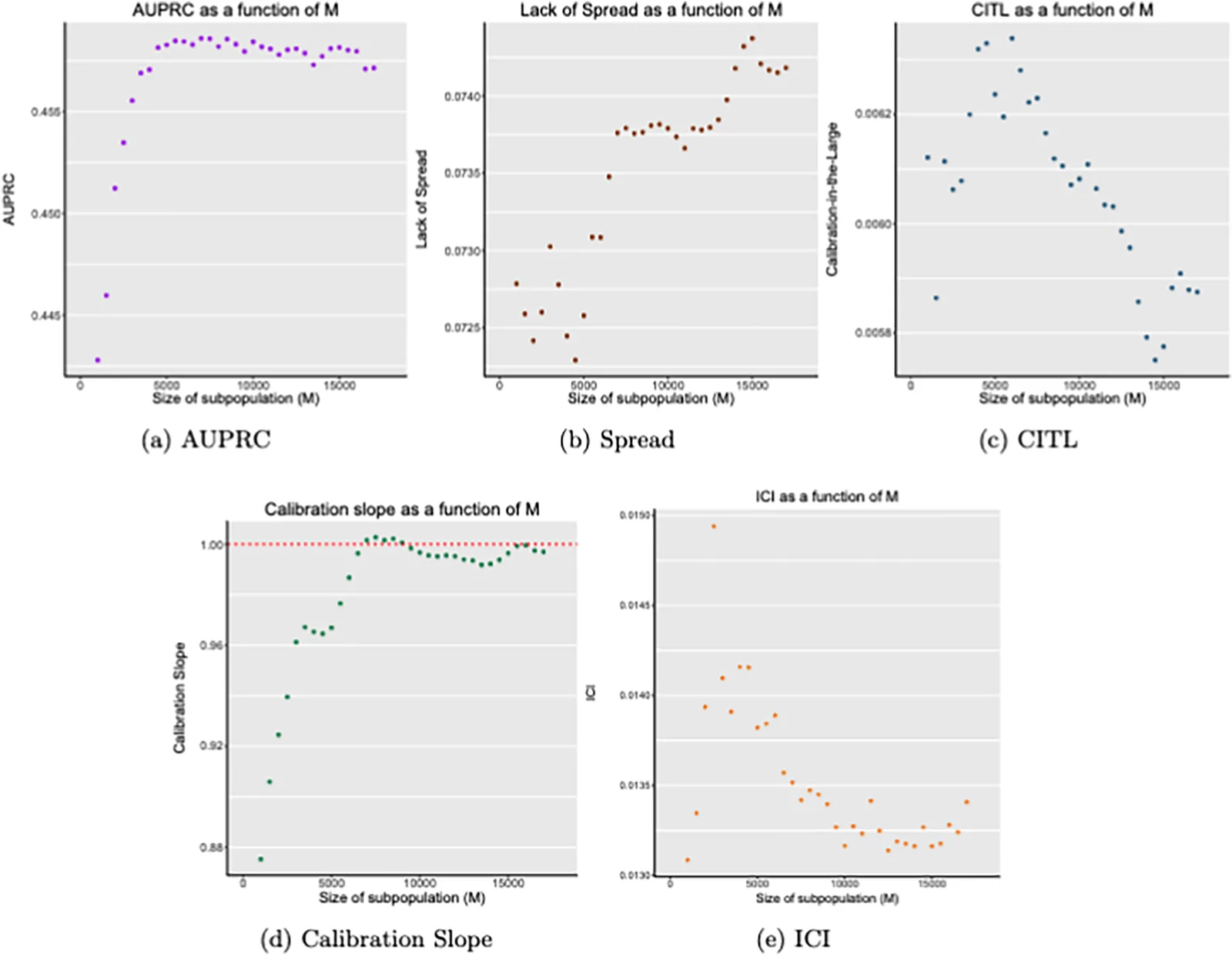
Performance measures as a function of M for the circulatory disease patient dataset from the eICU database. The Monte Carlo Simulation Error (MCSE) across all values of M for all five performance measures considered was less than 0.002.

After investigating the relationships between M and the performance measures, we then applied the proposed algorithm from Section 2.4 to the eICU data. We applied this algorithm twice, considering two different loss functions. The first is the same loss function used in the simulation studies, shown in equation (4), which consists of the ICI and the lack of spread measure, as the measures of calibration and discrimination, respectively. The second loss function we consider consists of the ICI as the calibration measure and the AUPRC as the discrimination measure, as shown below in equation (8). We approximate the AUPRC in the second term after the equal sign and define P(c) as the precision at a cutoff c , c=1,…,C , and △r(c) as the change in recall that occurred between cutoff c−1 and cutoff c . We use ( 1−AUPRC ) as the measure since higher values of the AUPRC correspond to better discriminative ability, and we wish to minimize $L^{**}$ .

(8) $$L^{**(M)}=\frac{\alpha }{n_{\mathrm{test}}}\sum_{I=1}^{n_{\mathrm{test}}}\left|{\hat{p_{I}}}^{\left(M\right)}-p_{I}^{\left(M\right)}\right|+(1-\alpha )\left[1-\sum_{c=1}^{d}P(c)△r(c)\right].$$

In both cases, we consider a grid of values to tune M which has a lower bound of 20% of the entire training dataset, and an upper bound of 70% of the entire training dataset, as per our recommendations given in Section 3. We choose 70% as the upper bound, which we recommended if the interest lies in calibration, rather than the stricter 50% in this data analysis just as a proof of concept, to check where the optimal M values lie in the grid of values considered under the various values of α .

We begin by displaying the results obtained when using equation (4) as the loss function. A dot plot showing the resulting optimal M proportions is shown in Figure 5(a), and is consistent with what we expected: since both moderate calibration and the lack of spread perform well when M is low, the optimal M value would be low regardless of the choice of α . The optimal M proportion under all values of α is 0.29, except α = 0.9 where the optimal M proportion is 0.20.

**Figure 5.**
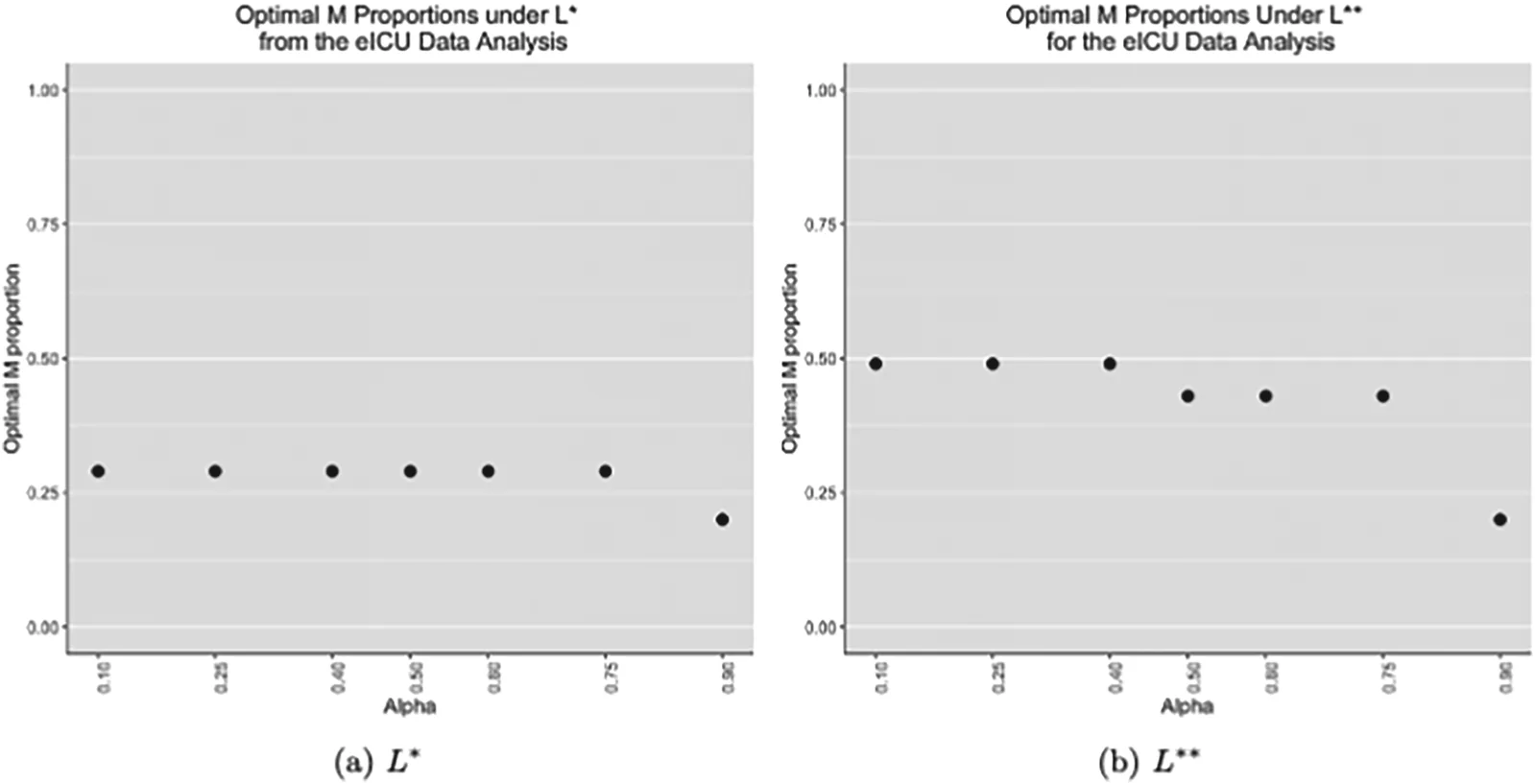
Optimal *M* proportions found using L* from equation (5), and L** from equation (8), for varying values of α found when tuning *M* during the training/testing step of applying the proposed algorithm to the eICU dataset.

Following the training/testing step, we used the optimal proportions shown in Figure 5(a) in the validation step and assessed the performance of the resulting models. The performance of the PPMs under the seven α values, and the performance of the model which uses the entire available training data, is shown in Table 2. Here, we report the BCa confidence interval and the bootstrap standard error in addition to the point estimates of the AUPRC, the lack of spread, CITL, calibration slope, and the ICI. We note that under both the lack of spread measure and the ICI, both of which were included in the loss function used to tune the size of subpopulation, a model fit on a similar subpopulation performs better than one fit on the entire training dataset (i.e. Proportion = 1.0). Specifically, when α=0.1 , the resulting optimal M proportion is 0.29, which is the best performing PPM in terms of lack of spread, with the measure valued at 0.063, compared to the full model, which has a lack of spread value of 0.071. This is expected as low values of α result in a larger emphasis on the discrimination term in the loss function. When α=0.9 , thus giving a larger emphasis on calibration in the loss function, the optimal M proportion is 0.2 and the value of the ICI corresponding to that PPM is 0.013 which is the best moderately calibrated model, which might seem somewhat counter-intuitive with the high α , that is, larger emphasis on calibration, at least based on the simulation results. This said, the difference between the best and worst moderately calibrated model is 0.003, suggesting these small differences are in line with what was found in Figure 4 when investigating the relationship between M and the ICI.

**Table 2.** Model performance in validation step of data analysis using the circulatory disease patient data from the eICU dataset under L*: the loss function which consists of the lack of spread and the ICI.

alpha	Proportion	AUPRC	Lack of Spread	CITL	Slope	ICI
0.1	0.29	0.475 (0.060)	0.063 (0.004)	0.021 (0.006)	1.015 (0.092)	0.015 (0.005)
		(0.363, 0.607)	(0.059, 0.073)	(0.017, 0.023)	(0.914, 1.129)	(0.006, 0.016)
0.25	0.29	0.475 (0.060)	0.063 (0.004)	0.021 (0.006)	1.015 (0.092)	0.015 (0.005)
		(0.363, 0.607)	(0.059, 0.073)	(0.017, 0.023)	(0.914, 1.129)	(0.006, 0.016)
0.4	0.29	0.475 (0.060)	0.063 (0.004)	0.021 (0.006)	1.015 (0.092)	0.015 (0.005)
		(0.363, 0.607)	(0.059, 0.073)	(0.017, 0.023)	(0.914, 1.129)	(0.006, 0.016)
0.5	0.29	0.475 (0.060)	0.063 (0.004)	0.021 (0.006)	1.015 (0.092)	0.015 (0.005)
		(0.363, 0.607)	(0.059, 0.073)	(0.017, 0.023)	(0.914, 1.129)	(0.006, 0.016)
0.6	0.29	0.475 (0.060)	0.063 (0.004)	0.021 (0.006)	1.015 (0.092)	0.015 (0.005)
		(0.363, 0.607)	(0.059, 0.073)	(0.017, 0.023)	(0.914, 1.129)	(0.006, 0.016)
0.75	0.29	0.475 (0.060)	0.063 (0.004)	0.021 (0.006)	1.015 (0.092)	0.015 (0.005)
		(0.363, 0.607)	(0.059, 0.073)	(0.017, 0.023)	(0.914, 1.129)	(0.006, 0.016)
0.9	0.20	0.441 (0.060)	0.066 (0.004)	0.021 (0.007)	0.981 (0.090)	0.013 (0.007)
		(0.358, 0.529)	(0.058, 0.075)	(0.020, 0.025)	(0.905, 1.057)	(0.007, 0.018)
	1.0	0.478 (0.060)	0.071 (0.004)	0.022 (0.006)	1.024 (0.099)	0.016 (0.005)
		(0.375, 0.594)	(0.062, 0.076)	(0.020, 0.026)	(0.878, 1.198)	(0.005, 0.027)

The performance of the personalized predictive modells (PPMs) is shown under seven values of α , and compared to the model where the entire available data is used to train the PPMs. We report the BCa confidence intervals and bootstrap standard errors in addition to the point estimates of the AUPRC, the lack of spread, CITL, calibration slope, and the ICI.

The AUPRC, which was not considered when tuning M but is a more commonly used measure of discrimination than lack of spread, is the best when the model is fit to the entire training dataset. Under the entire training dataset, the AUPRC is valued at 0.478, while under the optimal M proportion when α=0.1 , the resulting PPM has an AUPRC value of 0.475. Based on the investigations shown in Figure 4, the optimal M proportion under the AUPRC is expected to be approximately 0.5, with small differences between the AUPRC of models fit on subpopulations with sizes larger than 50% of the training data. We explore the changes in optimal M proportion when changing the measure of discrimination in the loss function from the lack of spread to the AUPRC later in this section.

Under the CITL, the best performing model is the one corresponding to an M proportion of 0.29. Although the point estimates of the CITL for the PPM fit on 29% and 20% of the entire training dataset are the same, the BCa confidence interval of the former shows better calibration than the latter. The model with the worst CITL is the full model. Similarly, the model with the best weak calibration, measured by the calibration slope, is the one corresponding to an M proportion of 0.29, while the worst model is the full model.

In this example, the best performing model would be the one corresponding to any of the α values except α = 0.9, since almost all performance measures are better under an M proportion of 0.29. Further, even though this is not the optimal model under the ICI, the difference between the point estimates of this model and the one which is optimal is 0.002, so we sacrifice little in terms of moderate calibration. For simplicity, given the results we observed, we will say that the best performing model corresponds to α = 0.5, which had a resulting optimal M proportion of 0.29.

We now discuss the results obtained using the second loss function, $L^{**}$ , described in equation (8), in applying our algorithm to the eICU circulatory disease dataset. Recall that this loss function consists of the AUPRC and the ICI as measures of discrimination and calibration, respectively. Based on the relationship between M and the AUPRC seen in Figure 4, we expected the optimal M proportion under $L^{**}$ to be much higher for low values of α compared to what was optimal using $L^{*}$ as the loss function, as shown in Figure 5(a). The results of this investigation are shown in Figure 5(b), where our hypothesis is shown to be correct. Unlike the results using the lack of spread as the measure of discrimination, here, for low values of α , the optimal M proportion is 0.49. The optimal M proportion remains at 0.49 until we set α to be 0.5, which places equal weight on both the AUPRC and the ICI in the loss function, where the proportion then decreases slightly to 0.43. Then, for α=0.9 , the optimal M proportion is 0.2, which is exactly what was found under the previous loss function. This is not surprising, as in both loss functions considered, we used the same measure of calibration. These results match the relationships between M and the AUPRC and M and the ICI we observed in Figure 4(a) and (e), respectively.

Using the optimal M proportion found under each value of α , we then validated the results and display our findings in Table 3. We immediately notice that the AUPRC of the model corresponding to α=0.1 has improved compared to the results using the prior loss function, shown in Table 2, and it outperforms the AUPRC of the full model. The resulting AUPRC performance confirms the relationship between M and the AUPRC we observed in Figure 4, where the model with the optimal AUPRC is fit on approximately 50% of the full training dataset, but the difference in AUPRC values between the optimal and full models is very small. Specifically, here, the optimal model, which is fit on a similar subpopulation made up of 49% of the patients from the full training dataset, has an AUPRC value of 0.481, while the full model has an AUPRC value of 0.478. As expected, the lack of spread of the model is not optimal under an M proportion of 0.49, with a value that is 0.006 units higher than what was displayed in Table 2 when we set α=0.1 , however, the performance is still better than the full model, which, again, confirms the relationships seen in Figure 4.

**Table 3.** Model performance in validation step of data analysis using the circulatory disease patient data from the eICU dataset under L**: the loss function which consists of the AUPRC and the ICI.

alpha	Proportion	AUPRC	Lack of Spread	CITL	Slope	ICI
0.1	0.49	0.481 (0.060)	0.069 (0.004)	0.022 (0.006)	1.024 (0.099)	0.016 (0.006)
		(0.375, 0.595)	(0.059, 0.075)	(0.020, 0.026)	(0.878, 1.198)	(0.005, 0.027)
0.25	0.49	0.481 (0.060)	0.069 (0.004)	0.022 (0.006)	1.024 (0.099)	0.016 (0.006)
		(0.375, 0.595)	(0.059, 0.075)	(0.020, 0.026)	(0.878, 1.198)	(0.005, 0.027)
0.4	0.49	0.481 (0.060)	0.069 (0.004)	0.022 (0.006)	1.024 (0.099)	0.016 (0.006)
		(0.375, 0.595)	(0.059, 0.075)	(0.020, 0.026)	(0.878, 1.198)	(0.005, 0.027)
0.5	0.43	0.475 (0.060)	0.068 (0.004)	0.021 (0.006)	0.848 (0.097)	0.014 (0.005)
		(0.369, 0.593)	(0.060, 0.074)	(0.020, 0.030)	(0.660, 0.979)	(0.005, 0.023)
0.6	0.43	0.475 (0.060)	0.068 (0.004)	0.021 (0.006)	0.848 (0.097)	0.014 (0.005)
		(0.369, 0.593)	(0.060, 0.074)	(0.020, 0.030)	(0.660, 0.979)	(0.005, 0.023)
0.75	0.43	0.475 (0.060)	0.068 (0.004)	0.021 (0.006)	0.848 (0.097)	0.014 (0.005)
		(0.369, 0.593)	(0.060, 0.074)	(0.020, 0.030)	(0.660, 0.979)	(0.005, 0.023)
0.9	0.20	0.441 (0.060)	0.066 (0.004)	0.021 (0.007)	0.981 (0.009)	0.013 (0.007)
		(0.358, 0.529)	(0.058, 0.075)	(0.020, 0.025)	(0.905, 1.057)	(0.007, 0.018)
	1.0	0.478 (0.060)	0.071 (0.004)	0.022 (0.006)	1.024 (0.099)	0.016 (0.005)
		(0.375, 0.594)	(0.062, 0.076)	(0.020, 0.026)	(0.878, 1.198)	(0.005, 0.027)

The performance of the personalized predictive modells (PPMs) is shown under seven values of α , and compared to the model where the entire available data is used to train the PPMs. We report the BCa confidence intervals and bootstrap standard errors in addition to the point estimates of the AUPRC, the lack of spread, CITL, calibration slope, and the ICI.

As we did not change the measure of calibration used to tune the size of subpopulation in the loss function, we do not see many changes in the calibration of these models. The model with the best moderate calibration is still the model corresponding to an M proportion of 0.2, which was determined when we put most of the weight in the loss function on the calibration term (i.e. α=0.9 ). The model with the best mean calibration, measured through the CITL, is the one which is fit on 43% on the full training dataset, while the model with the best weak calibration is the one which is fit on 20% of the training dataset.

The overall optimal model using $L^{**}$ as the loss function to tune M is not as clear of a decision as we observed when using $L^{*}$ as the loss function. Here, the user would have to make a decision as to what they value more and what measure they are willing to sacrifice. As the differences between measures are very small, these sacrifices will in turn be small in size. We define the optimal model of this study as the one which results in the smallest sacrifices in terms of performance, which, in this case, corresponds to α=0.1 , α=0.25 or α=0.4 , where all three result in an optimal M proportion of 0.49. Without loss of generality, we conclude that the model corresponding to α=0.4 is optimal, where this model has the best discrimination in terms of the AUPRC, and the lack of spread is 0.001 units less than its optimal value, the CITL is 0.001 units smaller than its optimal value, the calibration slope is 0.005 absolute units smaller than its optimal value and the ICI is 0.003 units smaller than its optimal value across all eight models considered.

## 5. Discussion

We introduced a general loss function on which to tune M when fitting a PPM which allows not only the flexibility of weighting one predictive model performance measure over another, but also allows flexibility in choosing which measures of model discrimination and calibration to be considered when optimizing the size of subpopulation. Further work is necessary to verify if this loss function is a proper scoring rule. Further, we investigated the relationship between M and various measures of model discrimination and calibration, and gave recommendations on bounds for the grid of values on which to tune M with the goal of reducing computation time.

We simulated 12 scenarios that differed by their true outcome model, and analyzed the relationships between M and six performance measures: AUROC, AUPRC, lack of spread, CITL, calibration slope and the ICI. In all cases, M had a negatively proportional relationship with measures of discrimination, aside from the lowest values of M when using the AUROC and the AUPRC as performance measures, so a relatively low value of M will result in a PPM with relatively better discriminative ability than a model fit on a larger subpopulation. In many cases, this low value is good enough, if not the best, for model calibration as well. The optimal M was often very similar under the calibration slope and the ICI, which are measures of weak calibration and moderate calibration, respectively. This agreement between the optimal M values did not always extend to the CITL, a measure of mean calibration, the lowest calibration measure in the calibration hierarchy^7^ which had an optimal M that was sometimes different than that under weak and moderate calibration. Although in many scenarios we observe a quadratic relationship between M and all three levels of calibration, the underlying reason for these patterns remains unclear. Future work should aim to determine whether an analytical characterization of the relationship between M and calibration can be derived, which may help explain these observed trends. We also conducted a small simulation study to assess whether changing the sample size has an effect on these relationships, and we found that there are not notable differences when either increasing or decreasing the sample size. However, a more extensive simulation study to test more sample size scenarios, in addition to more outcome prevalence scenarios, would be useful to investigate as future work.

Based on the results from the simulation study described above, we recommend that the grid of values on which to tune M should be bounded below by 20% of the training data, and bounded above by either 50% or 70% of the training data, depending on whether there is greater interest on model discrimination or calibration. A lower bound of 20% ensures that the sample size in the validation step will be large enough to ensure model stability, and corresponds to what we see as optimal under discrimination in most cases we considered. We also recommend, however, that researchers additionally consider the absolute number of outcome events when deciding on a lower bound, as an outcome that is rarer could need a larger lower bound to ensure reliable performance. An upper bound of 50% when there is equal interest in discrimination and calibration, or greater interest in discrimination (i.e. α<0.5 ) tightens the bound and decreases computation time while still likely capturing the optimal M value under discrimination. An upper bound of 70% when greater interest lies in calibration (i.e. α>0.5 ) increases the chance of capturing the optimal M under calibration if that value is large, while ensuring that we do not have too much deterioration in discriminative ability. Since a low value of M is good, if not the best, in many cases in terms of all levels of calibration, we can justify not having the entire size of the training dataset as the upper bound on the grid of values on which to tune M . By bounding the grid of values, computation time can be cut down significantly, which is important for when these methods are taken into practice, as PPM algorithms are computationally intensive, given that a unique model is fit for each individual in the test data. The cases we considered here are not part of an exhaustive list, and these recommendations on bounds are based on the 12 scenarios we did consider. Other datasets may differ in characteristics that may lead to the consideration of a wider grid, however, our recommendations serve, at the very least, as a useful starting point. Future work includes investigating these relationships under more scenarios, as well as more flexible models (e.g. random forest, or more complex machine learning models), as the relationships between M and calibration may differ under non-likelihood based models. We also note that in the proposed algorithm, we are determining an optimal size of subpopulation that is used for any incoming index patient. An area to explore is whether this optimal M should be different for distinct patients. Once again, the computational efficiency of the algorithm must be improved before tackling this extension, however, having a unique optimal size of subpopulation for different patients could potentially further improve predictive performance of the PPMs.

Through an extensive simulation study, we tested our proposed general loss function which consisted of the lack of spread as a measure of discrimination, and the ICI as a measure of calibration. We tested multiple values of α to find the optimal size of subpopulation when fitting a PPM and compared these results to the relationships investigated in Section 3.2. As we increased the weight put on the ICI term in the loss function, we found that the resulting optimal M matched what was optimal under the ICI measure in the relationships displayed in Figures 1 and 2. In future work, we wish to explore whether there are certain combinations of calibration and discrimination measures that can be used in the loss function shown in equation (3) that lead to improved loss and ultimately better predictive performance.

We also applied the proposed algorithm to a dataset from the eICU database to predict the mortality of patients with diseases of the circulatory system. We considered two different loss functions which both had the ICI as the measure of calibration, but differed in the defined measure of discrimination, which was either the lack of spread, as was done in the simulation study, or the AUPRC, a more common measure of discrimination, particularly when the prevalence of the outcome is small, which was the case for our data analysis. In the first loss function considered, where the discrimination in the loss function was the lack of spread, the optimal M proportion under all values of α was low, specifically, it was 0.29 for all values except for α=0.9 , where the optimal M was 0.2. In the second loss function considered, there was more variability in the optimal M proportions across α values, and the optimal M proportions when discrimination was emphasized (i.e. when α was a value close to 0), was a much higher value at 0.49. These results coincided with the relationships between M and spread, as well as M and the AUPRC which we saw in Figure 4: very low values of M correspond to low values of lack of spread, while moderate values of M correspond to higher values of the AUPRC. We fit PPMs to the hold-out dataset using the optimal M proportions found in the training/testing step and calculated BCa bootstrap confidence intervals for each of the point estimates of the performance measures in both cases. Under the first loss function, we concluded that the optimal model is the one corresponding to α=0.5 , which resulted in an optimal M proportion of 0.29, where all performance measures were optimized except the ICI, which performed optimally under α=0.2 (i.e. $M_{\mathrm{proportion}}=0.2$ ). The model fit to the full training dataset had the worst performance across all performance measures, except under the AUPRC, where it performed slightly better. When using the AUPRC as the discrimination in the loss function, we concluded that the optimal model corresponded to α=0.4 (i.e. $M_{\mathrm{proportion}}=0.49$ ), as choosing this model resulted in the smallest sacrifices made in calibration performance. Further, the full model, under the second loss function, was the worst performing model across all performance measures considered. Through this data analysis, we showed how the optimal model differs when changing the choice of performance measures to use in the loss function.

When comparing the relationships between M and the performance measures from the data analysis to what we saw in the simulation studies, we notice that the patterns are slightly different, specifically in the relationship between M and discrimination. In the data analysis, this relationship is not as obviously negatively-proportional as in the simulation study. It is important to note that in a case where all patients have the same distributional mean, a personalized approach to modeling is not preferred over a one-size-fits-all model, since using a larger dataset with exchangeable patients will result in more power. The dataset we used in this study could have a very large proportion of patients who are similar to each other, compared to the one we simulated in Section 3, resulting in smaller gains in performance. In practice, an exploratory analysis of the training data may be conducted prior to fitting PPMs to assess the degree of heterogeneity present in the population and to evaluate whether a personalized approach is likely to provide meaningful improvements over a global model. The presence of latent patient subgroups with distinct predictor–outcome relationships has been proposed as a key driver of the benefits of personalized prediction.^22^ One formal approach to this problem is hypothesis testing for subgroup structure using mixture regression models.^22^ In more applied settings, simpler exploratory techniques such as principal component analysis and clustering can be used to investigate whether patients exhibit structure in the covariate space and whether the covariates used to define similarity capture meaningful variation between subgroups. Evidence of well-separated clusters or strong covariate-driven structure would suggest greater potential benefit from personalized modeling, whereas a lack of structure or substantial overlap between patients would indicate that a global model may be sufficient.

This study has a few limitations. Computational intensity continues to be the greatest challenge to overcome before taking these algorithms into practice. We introduced some coding techniques to help improve computation time, such as the use of Rcpp^23^ and parallelization. We have also improved on the computation time by introducing bounds to the grid of values used to tune M . However, this is still not sufficient, especially if these methods are to be used in real time, for example in an intensive care unit, where decisions must be made very quickly. As mentioned prior, we focus our efforts on a relatively basic model, logistic regression, to explore these relationships between the size of subpopulation, and the effects of patient similarity methods on prediction, under a simple case. Next steps of this work include investigations under more sophisticated machine learning models, that is, to see what the optimal M values would be using the same data but under different models. However, this exploration needs to await further developments on computational efficiencies for the proposed algorithm.

## Supplemental Material

sj-pdf-1-smm-10.1177_09622802261488055 - Supplemental material for A general mixture loss function to optimize a personalized predictive model Supplemental material, sj-pdf-1-smm-10.1177_09622802261488055 for A general mixture loss function to optimize a personalized predictive model by Tatiana Krikella, Joel A Dubin in Statistical Methods in Medical Research

## Data Availability

The datasets analyzed during the current study are available in the eICU Collaborative Research Database. Researchers seeking to use this database are required to apply, with details at the following website: https://eicu-crd.mit.edu/gettingstarted/access/.
