# Supplementary material for: A general mixture loss function to optimize a personalized predictive model
Source: Stat Methods Med Res. 2026 Sep 14;35(10):2182–99. doi: 10.1177/09622802261488055 (PMC13688343; doi:10.1177/09622802261488055)
Supplement: Supplementary file 1 [file sj-pdf-1-smm-10.1177_09622802261488055.pdf]

Supplementary Material

Tatiana Krikella and Joel A. Dubin

S.1 Additional Plots

We include additional plots connected to our simulation study. In the manuscript, we showed the relationships between M and six performance measures under two cases, but we investigated these relationships across 12 varying datasets. We include the results from the other ten cases here.

Recall that the twelve datasets considered differed in the size of their association, which could have been either 10/40 or 20/40, by the prevalence of the outcome, which could have been low($\sim$0.05), moderate ($\sim$0.15) or balanced ($\sim$0.5), and by the linearity, or lack there-of, of the model.

S.1.1 Scenario 1: Linear, 10 Association, Low Prevalence

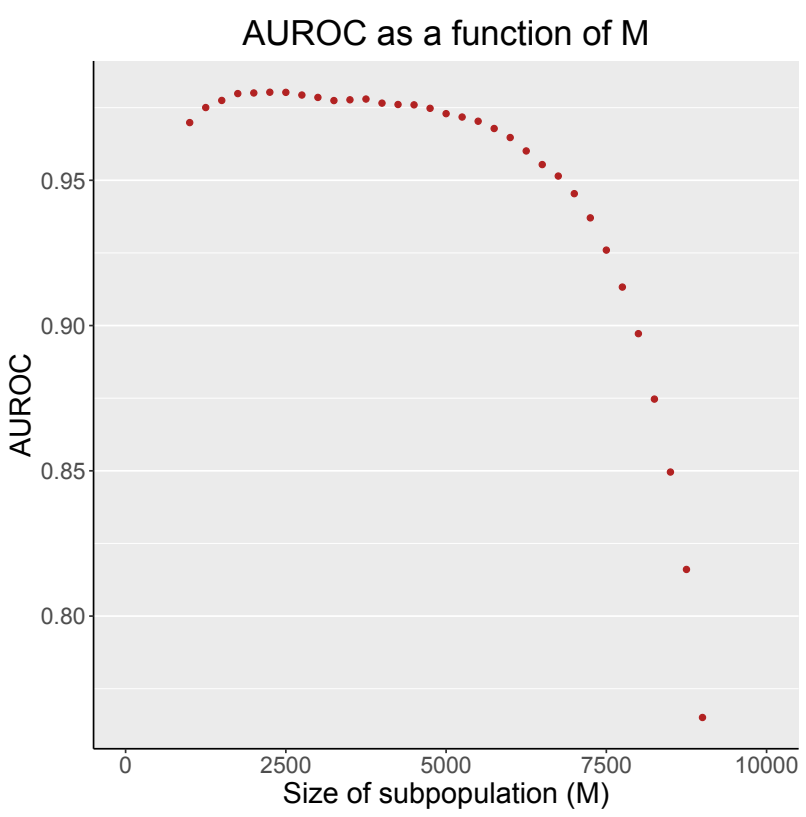

(a) AUROC

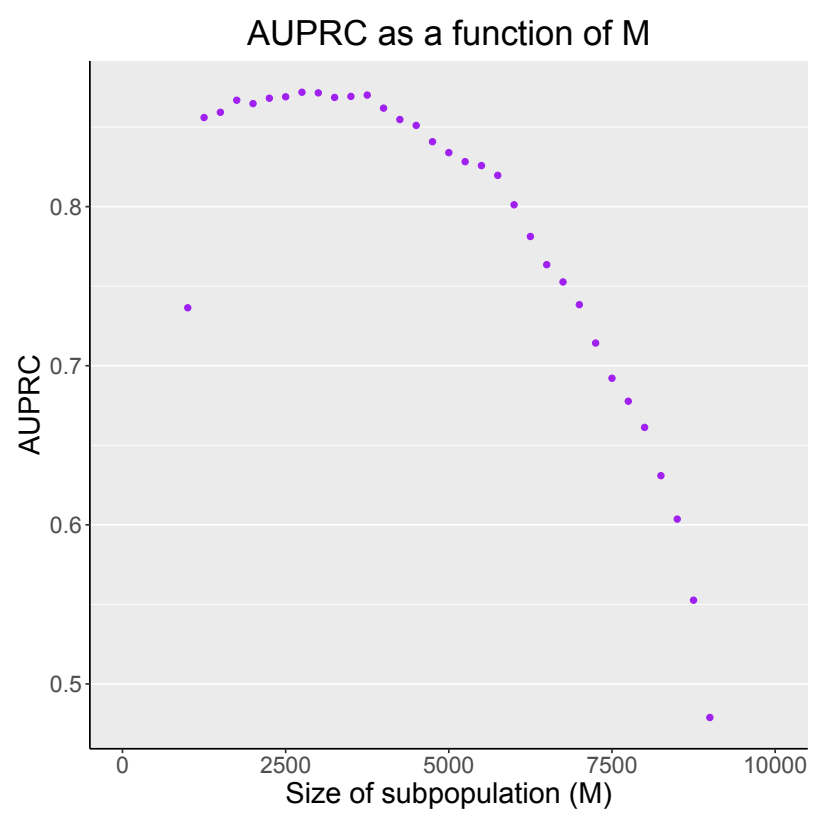

(b) AUPRC

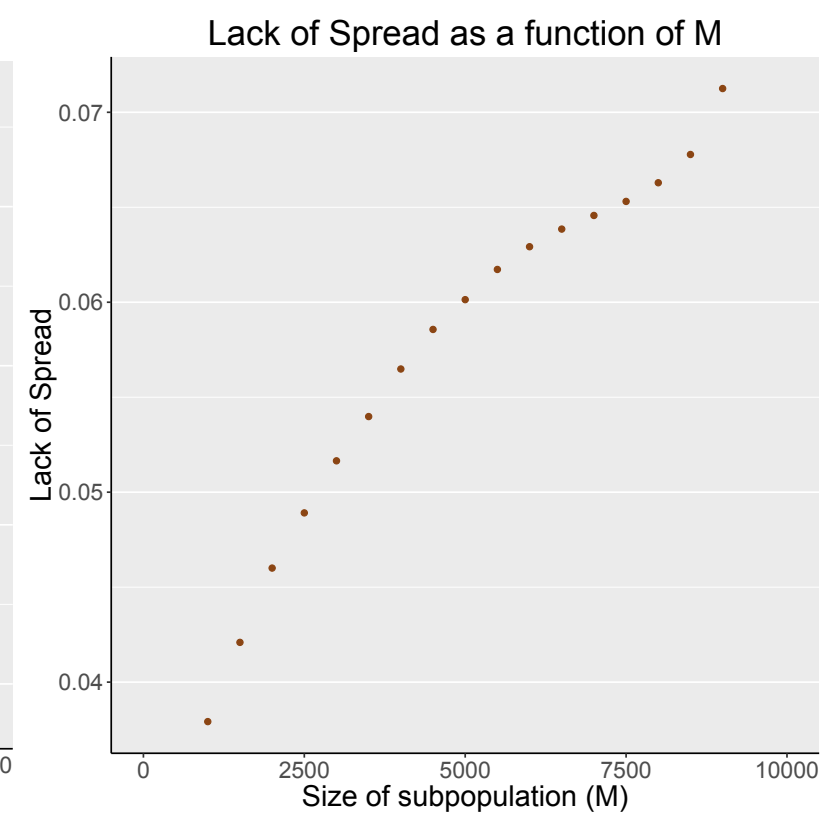

(c) Lack of Spread

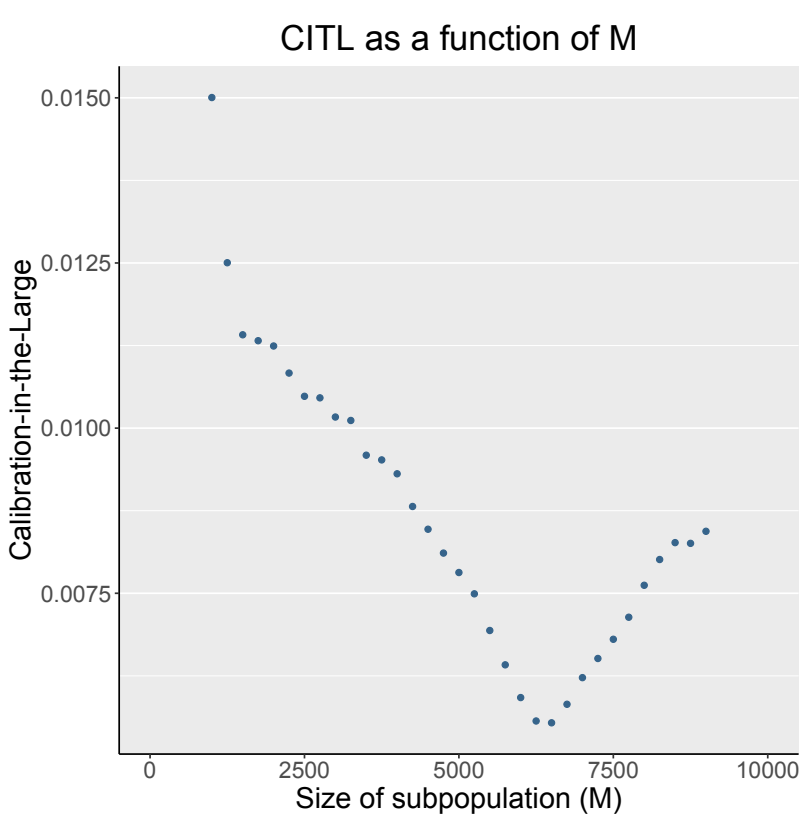

(d) CITL

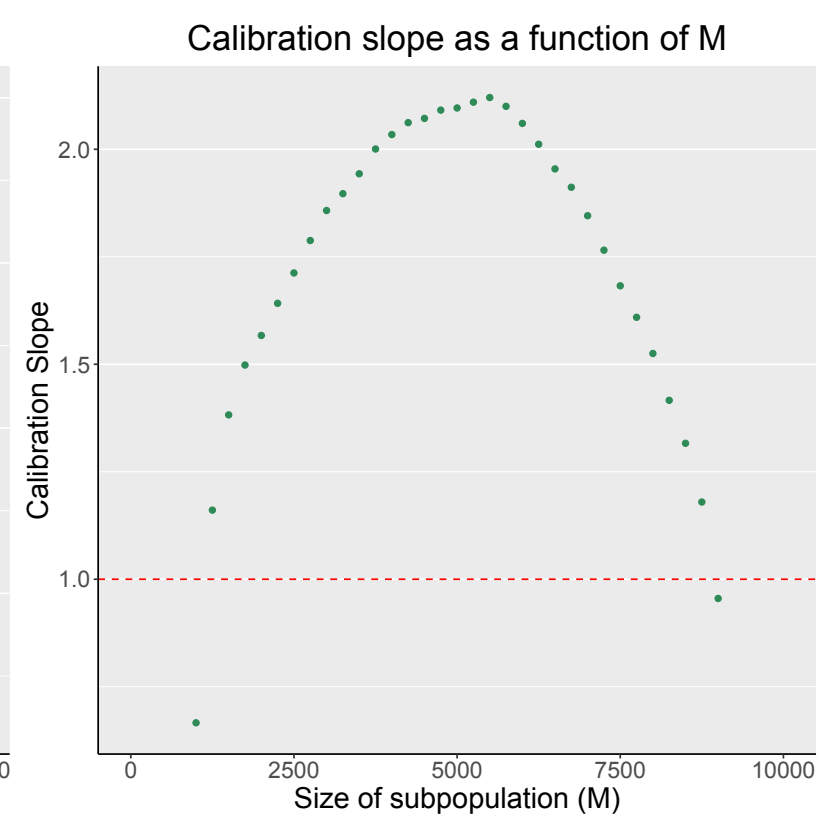

(e) Calibration Slope

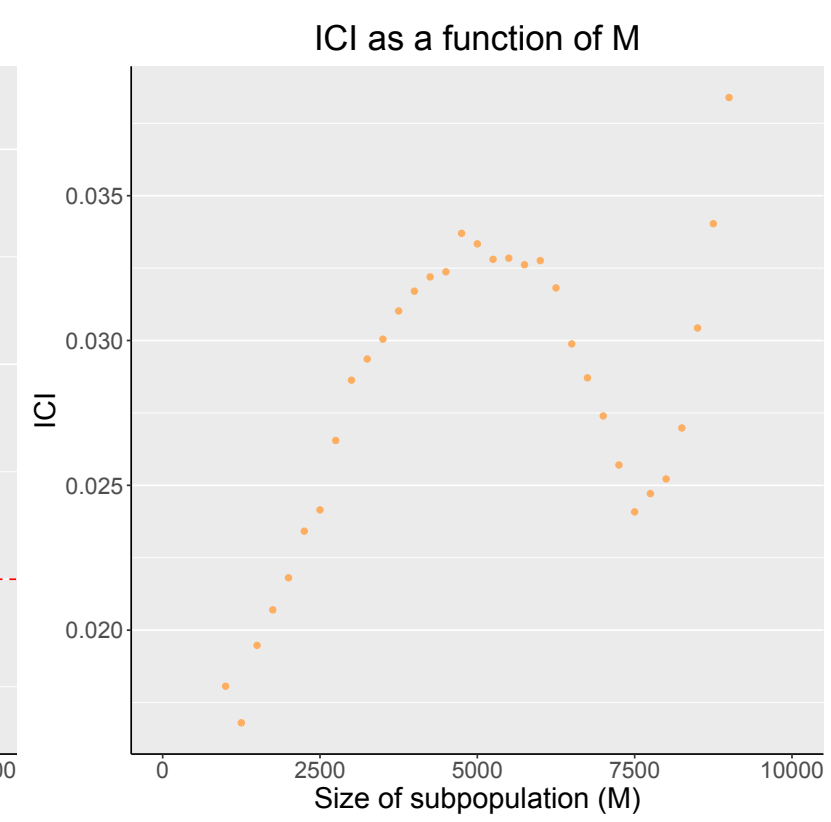

(f) ICI

Figure S1: Performance measures as a function of M when the outcome is simulated by a model which is linear, has 10 predictors associated with the outcome and results in a binary outcome with a prevalence of approximately 0.05.

S.1.2 Scenario 2: Linear, 10 Association, Moderate Prevalence

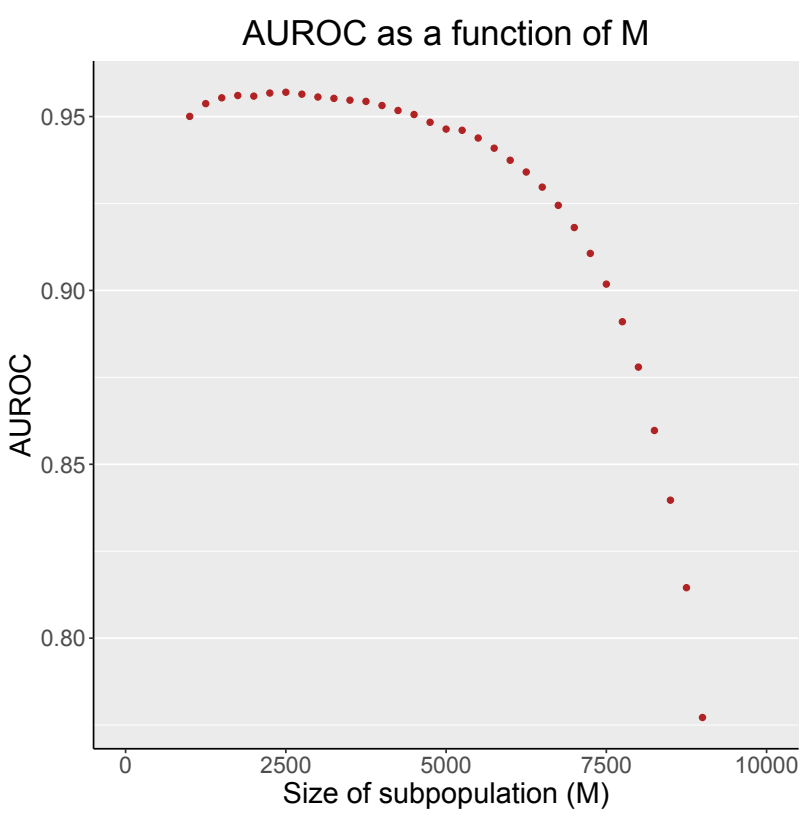

(a) AUROC

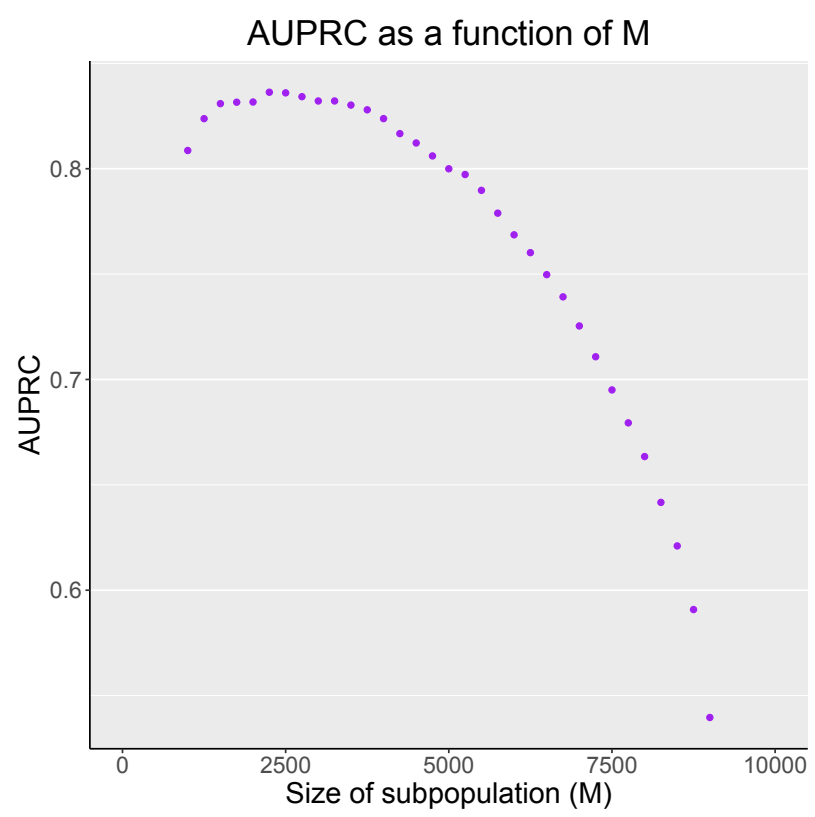

(b) AUPRC

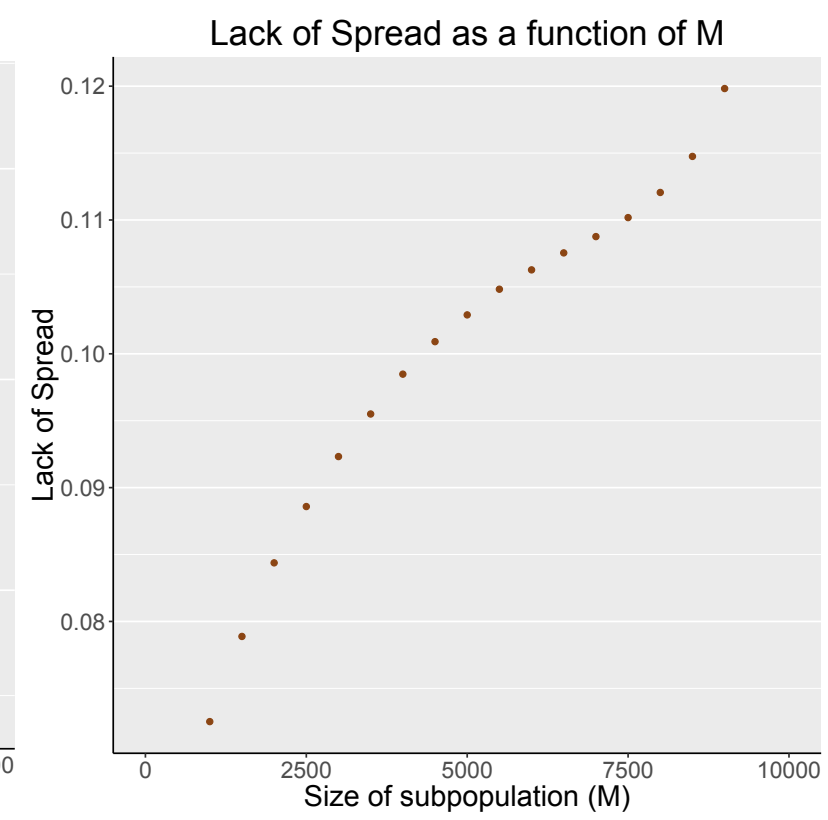

(c) Lack of Spread

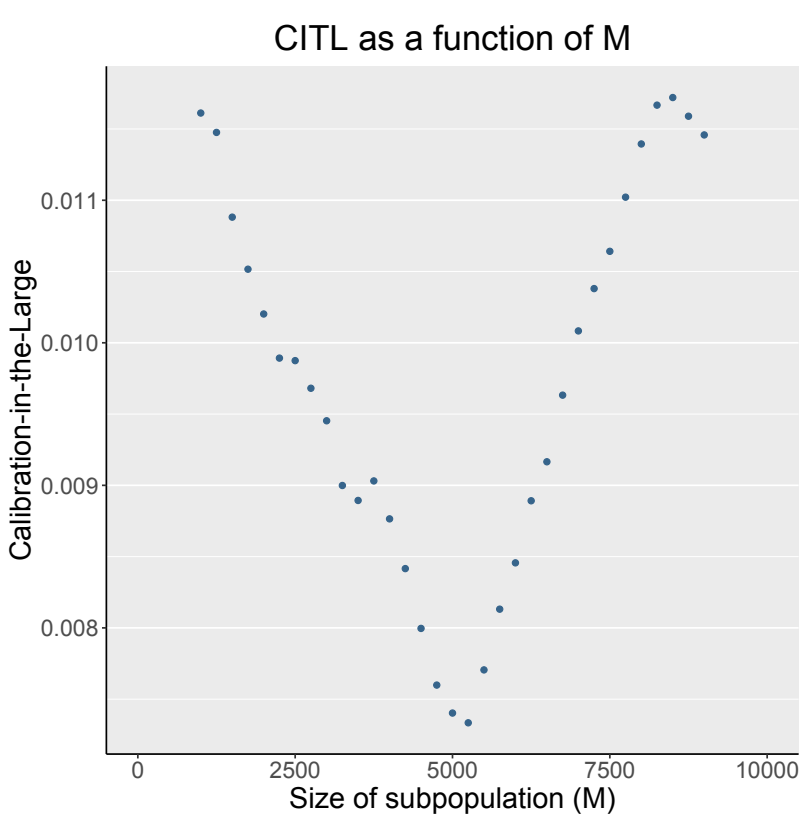

(d) CITL

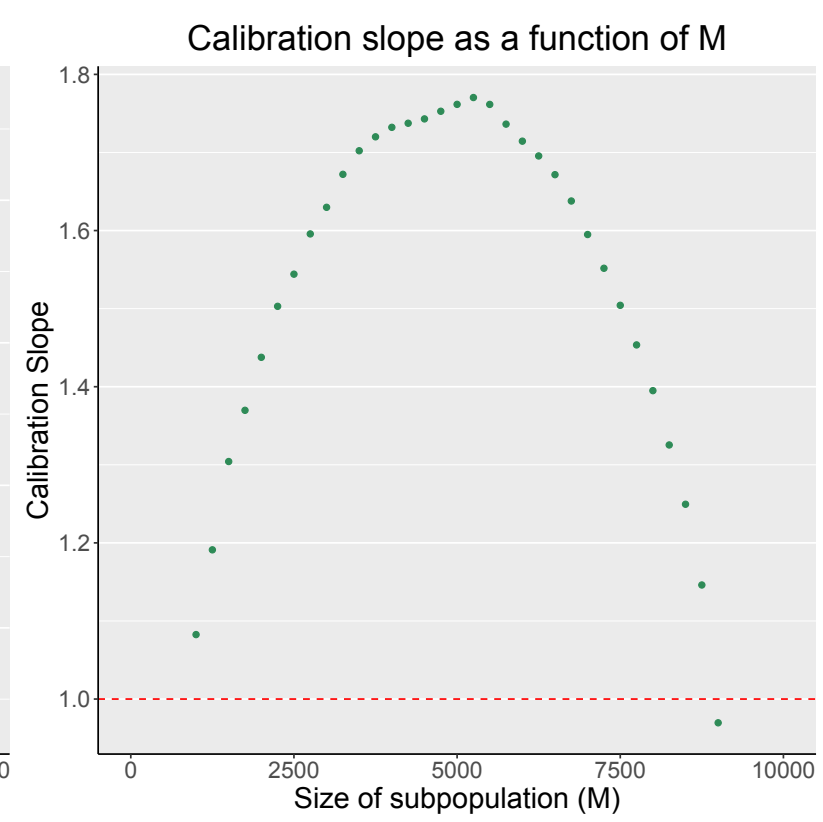

(e) Calibration Slope

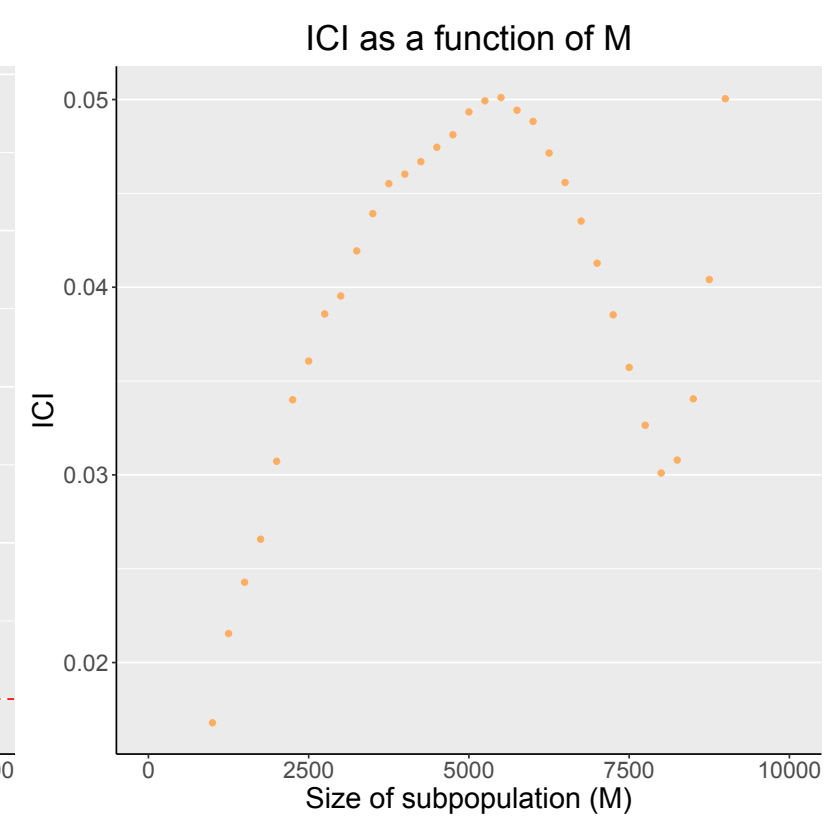

(f) ICI

Figure S2: Performance measures as a function of M when the outcome is simulated by a model which is linear, has 10 predictors associated with the outcome and results in a binary outcome with a prevalence of approximately 0.15.

S.1.3 Scenario 3: Linear, 10 Association, Balanced Prevalence

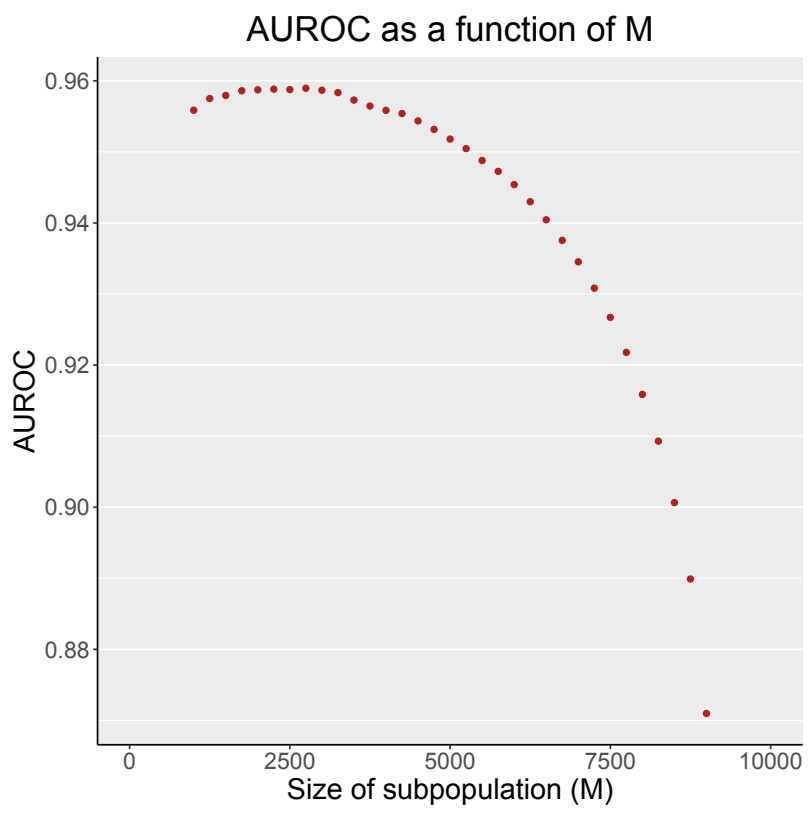

(a) AUROC

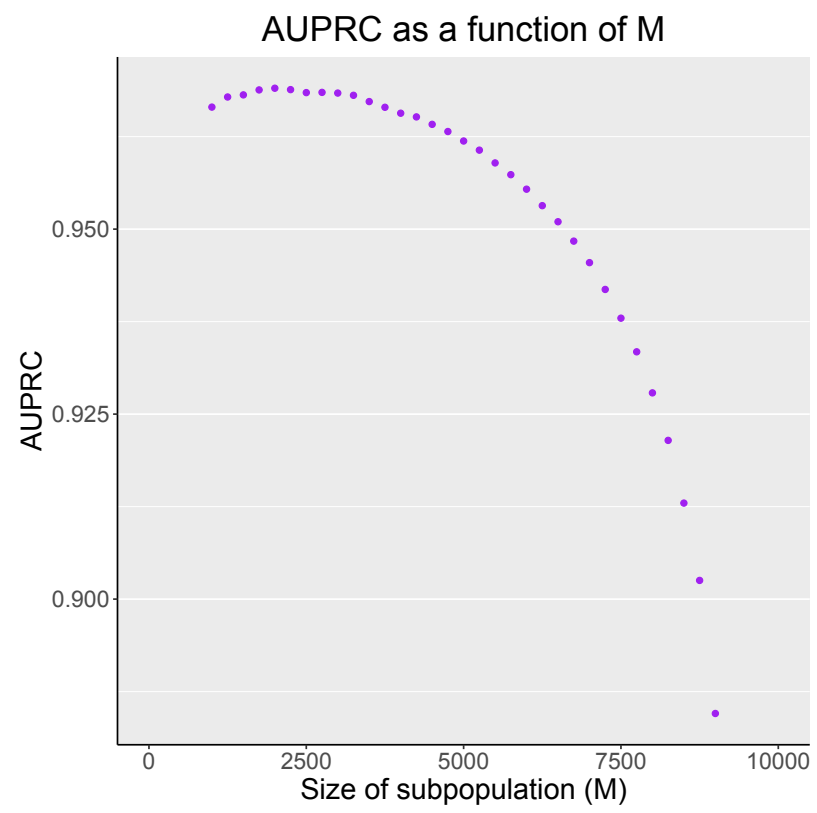

(b) AUPRC

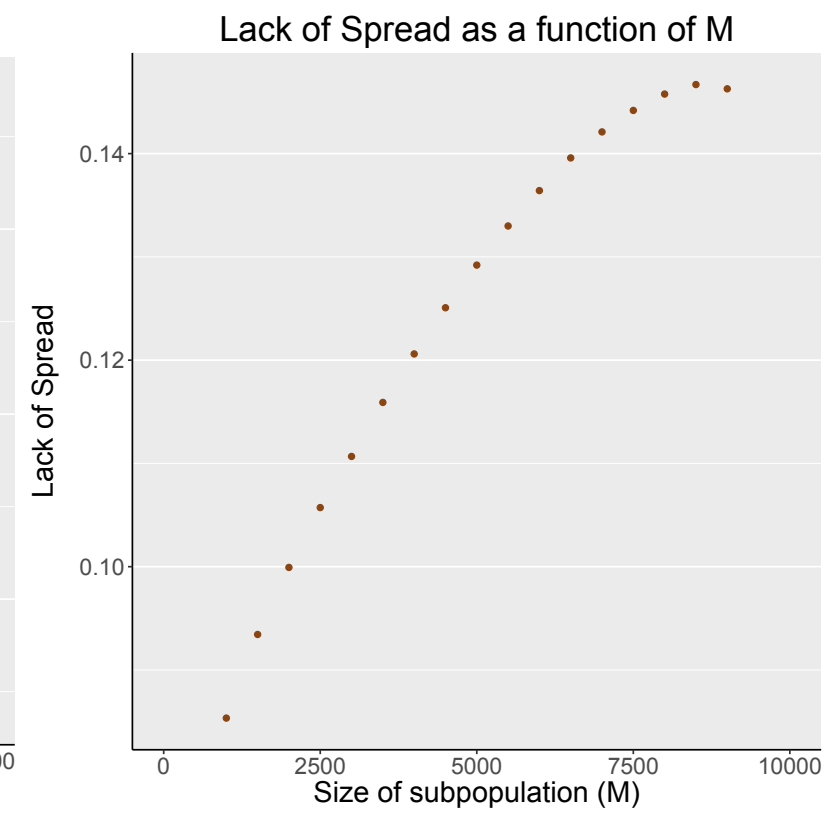

(c) Lack of Spread

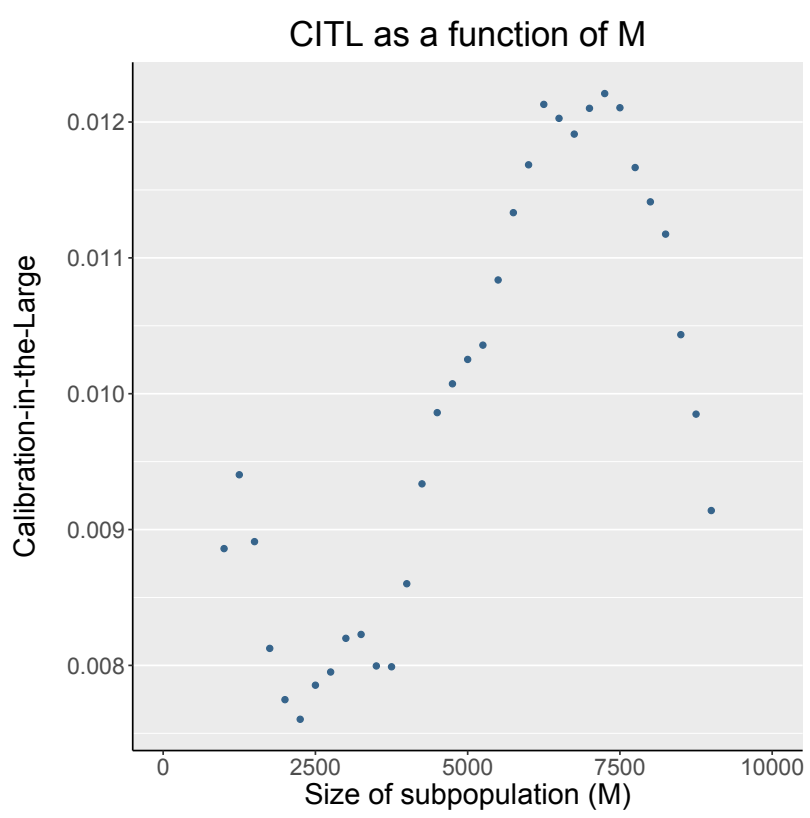

(d) CITL

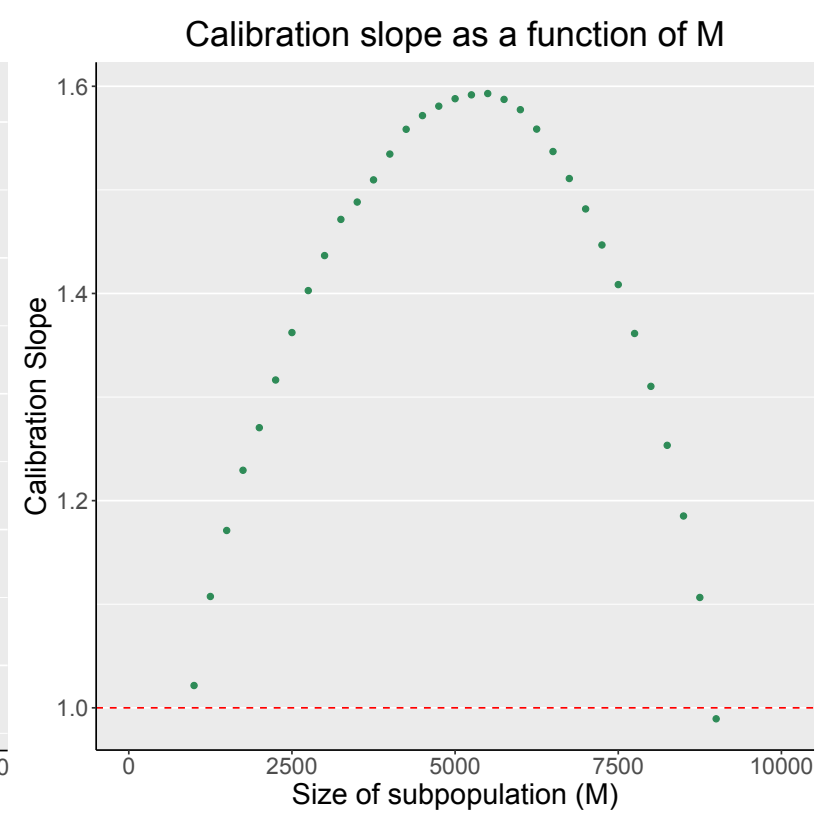

(e) Calibration Slope

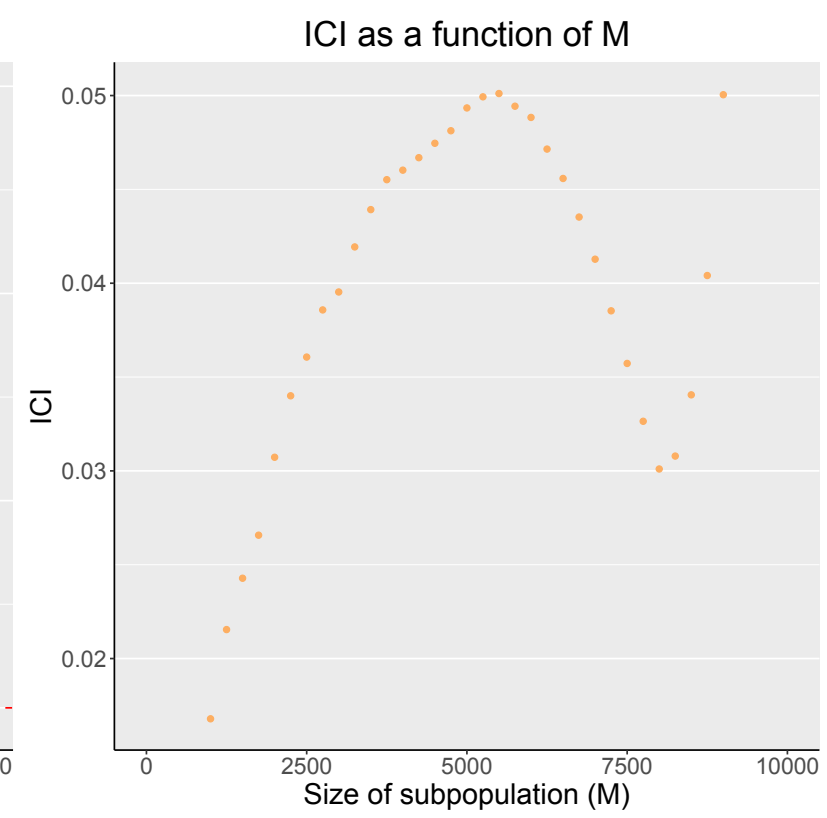

(f) ICI

Figure S3: Performance measures as a function of M when the outcome is simulated by a model which is linear, has 10 predictors associated with the outcome and results in a binary outcome with a prevalence of approximately 0.50.

S.1.4 Scenario 4: Linear, 20 Association, Low Prevalence

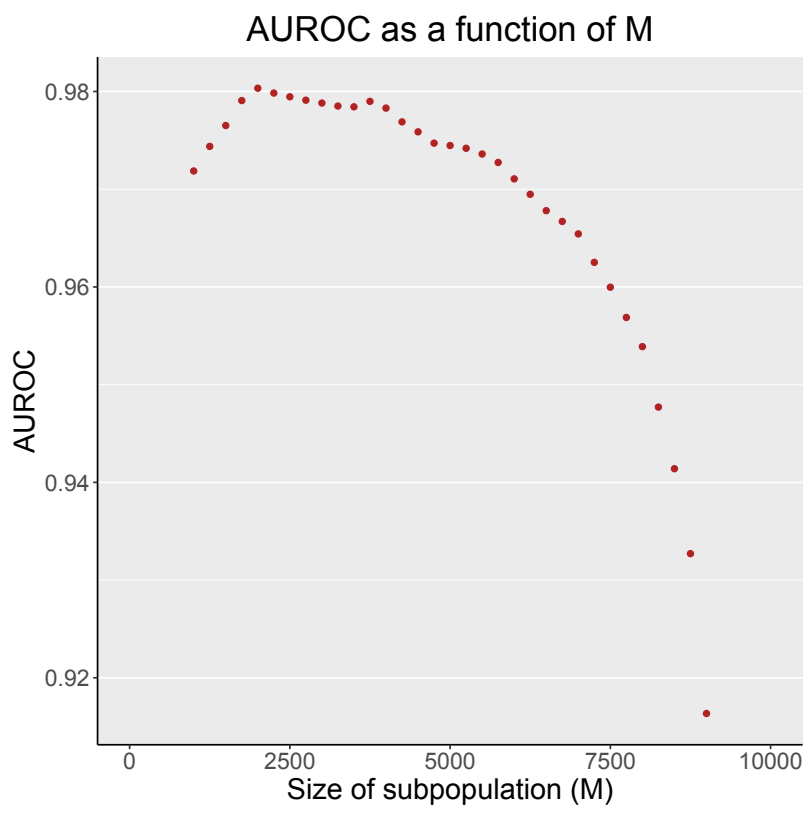

(a) AUROC

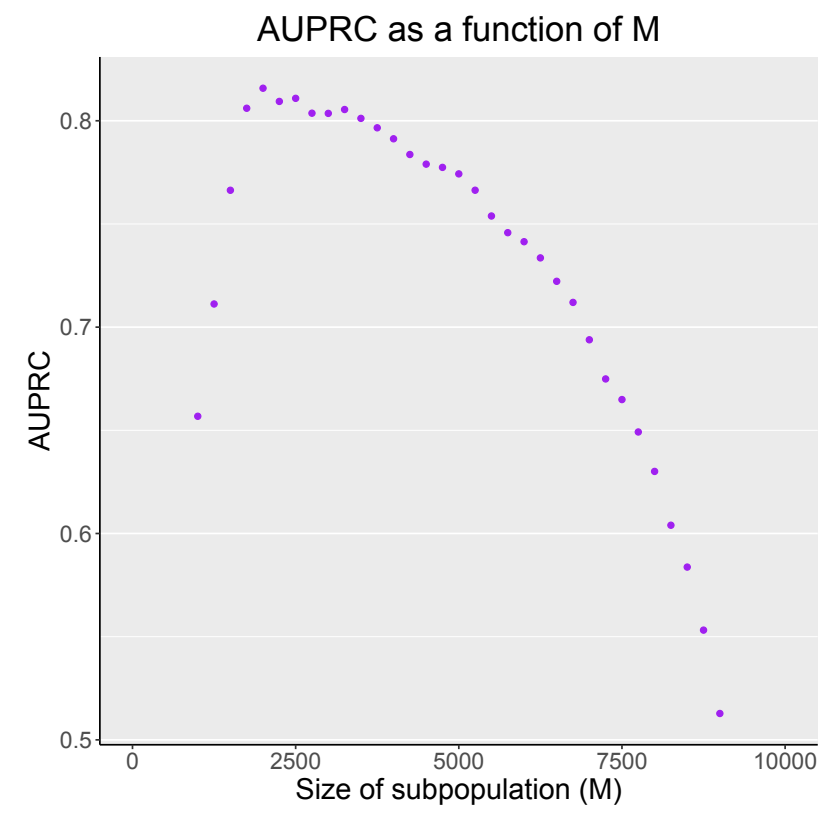

(b) AUPRC

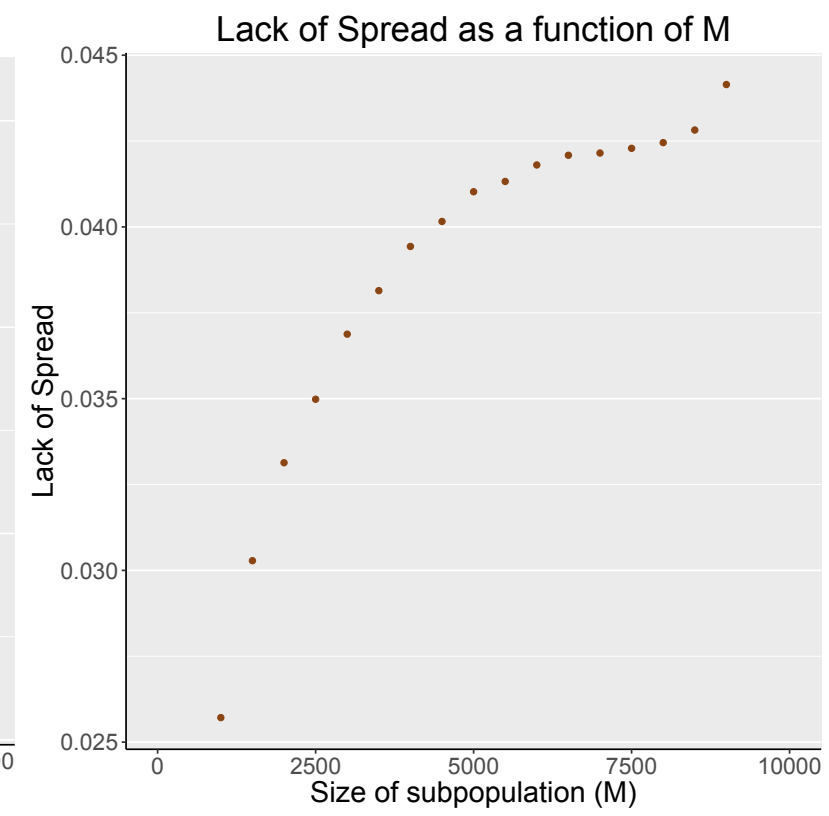

(c) Lack of Spread

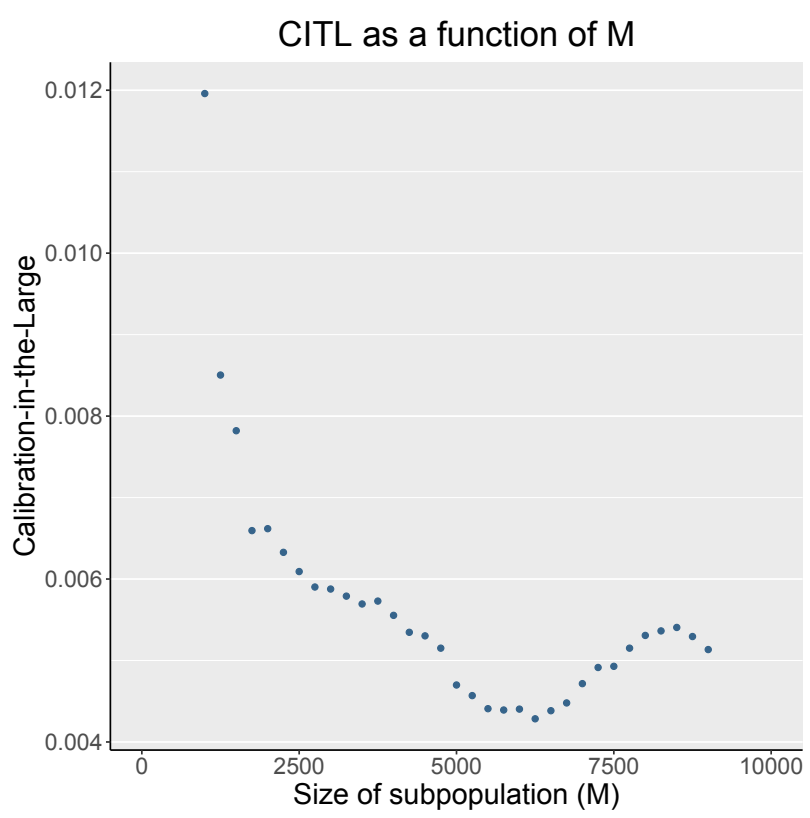

(d) CITL

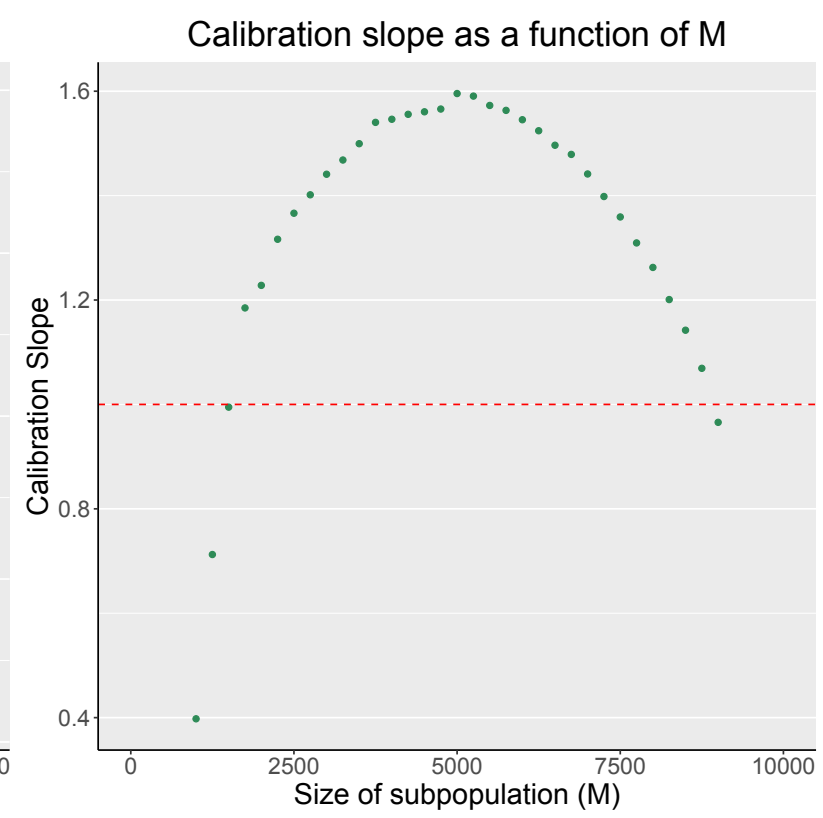

(e) Calibration Slope

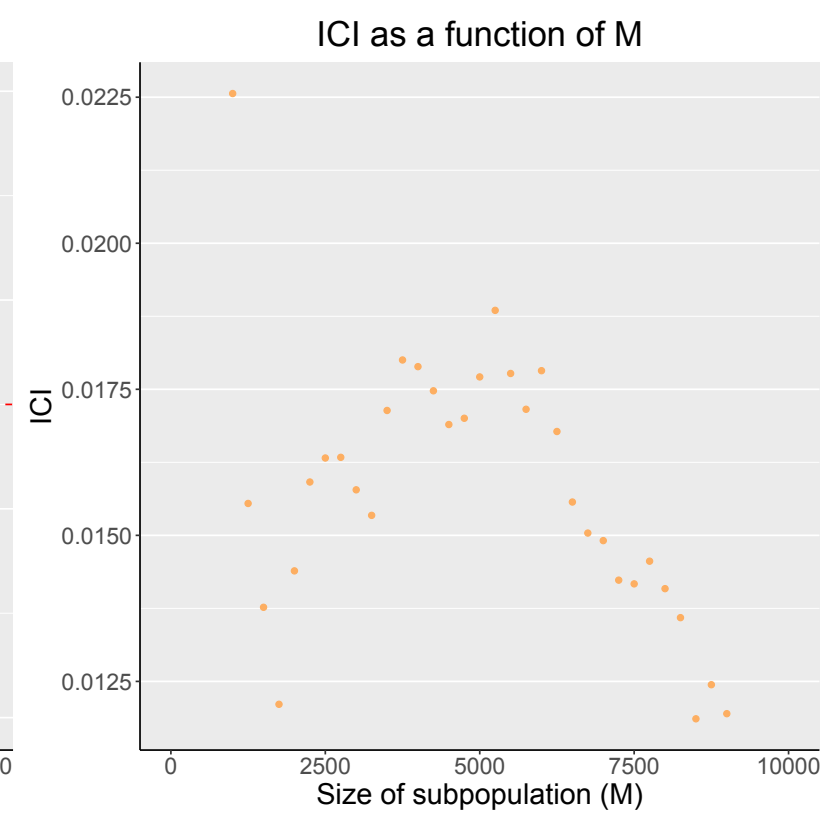

(f) ICI

Figure S4: Performance measures as a function of M when the outcome is simulated by a model which is linear, has 20 predictors associated with the outcome and results in a binary outcome with a prevalence of approximately 0.05.

S.1.5 Scenario 6: Linear, 20 Association, Balanced Prevalence

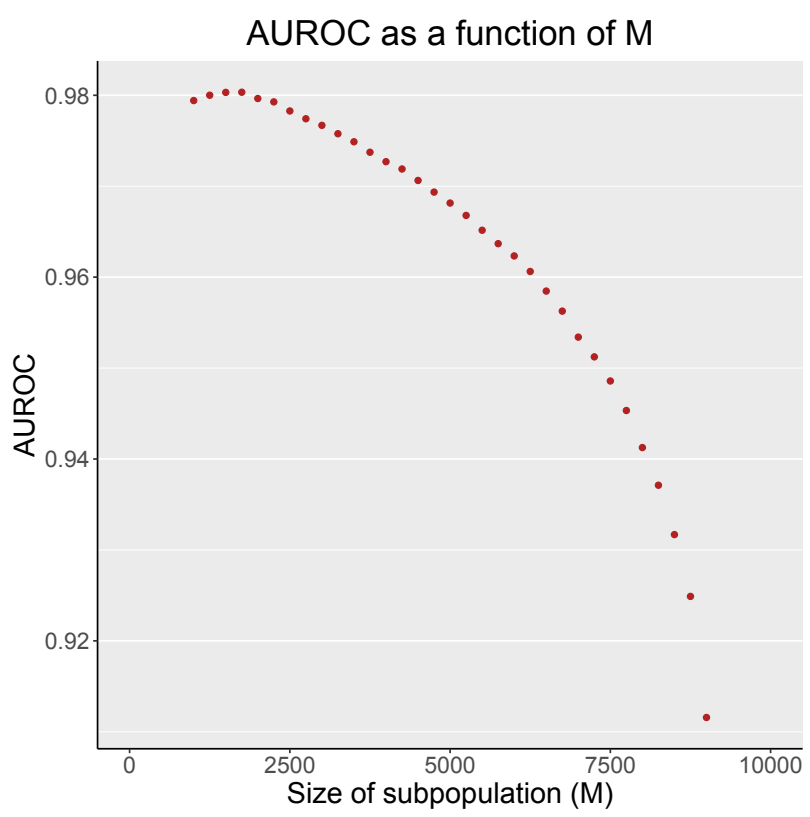

(a) AUROC

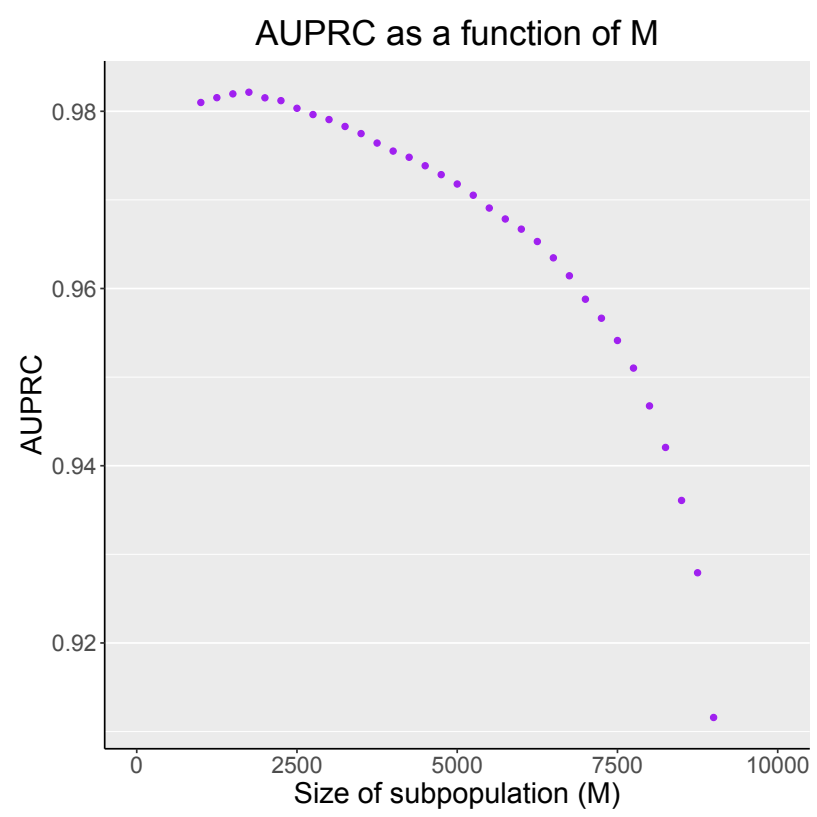

(b) AUPRC

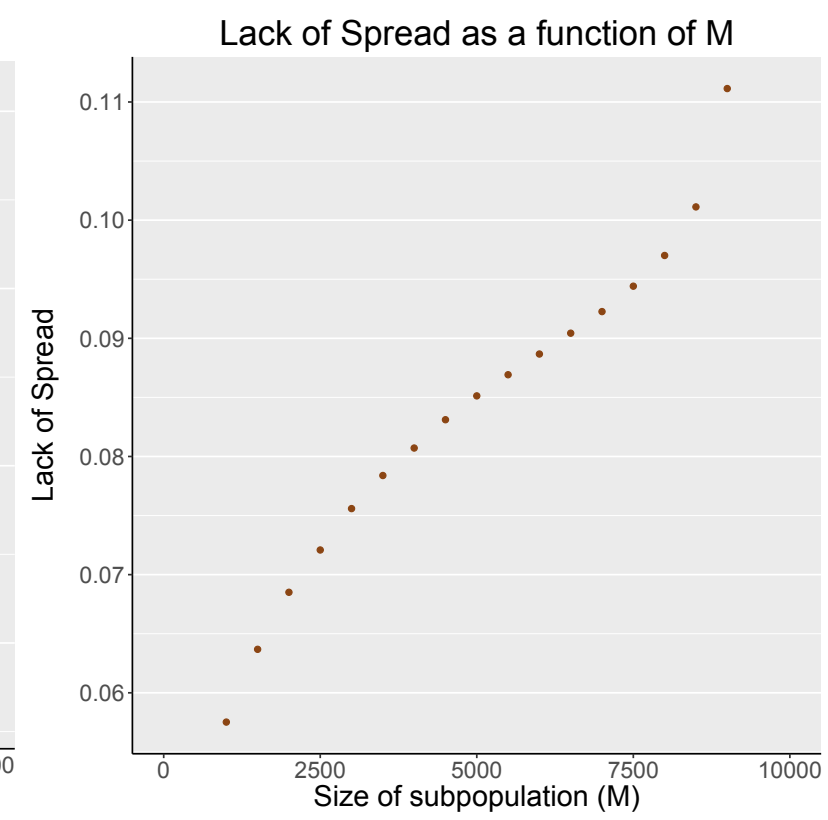

(c) Lack of Spread

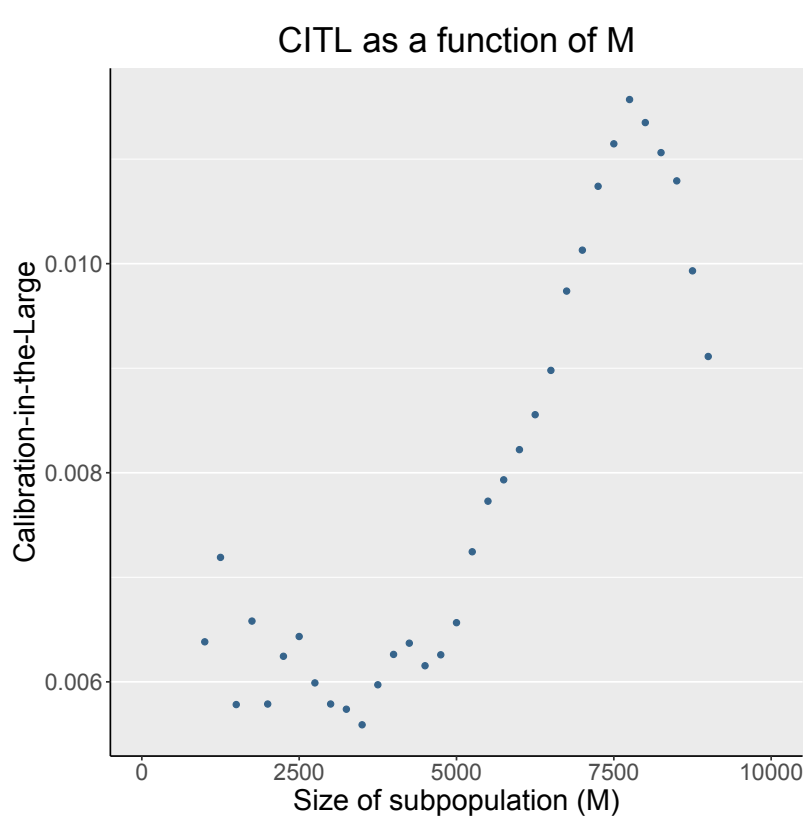

(d) CITL

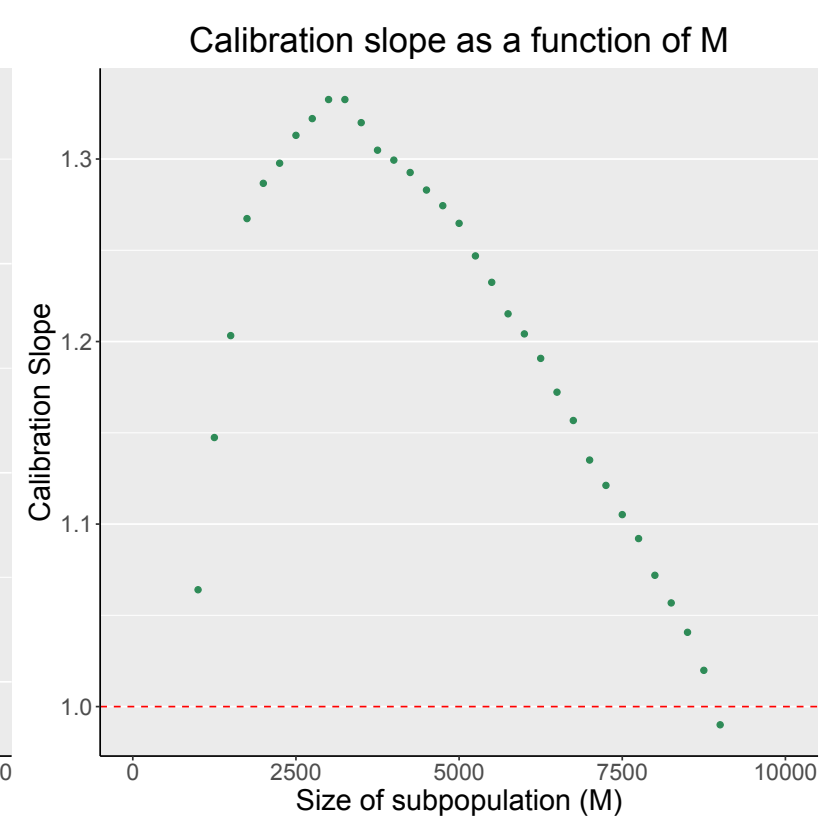

(e) Calibration Slope

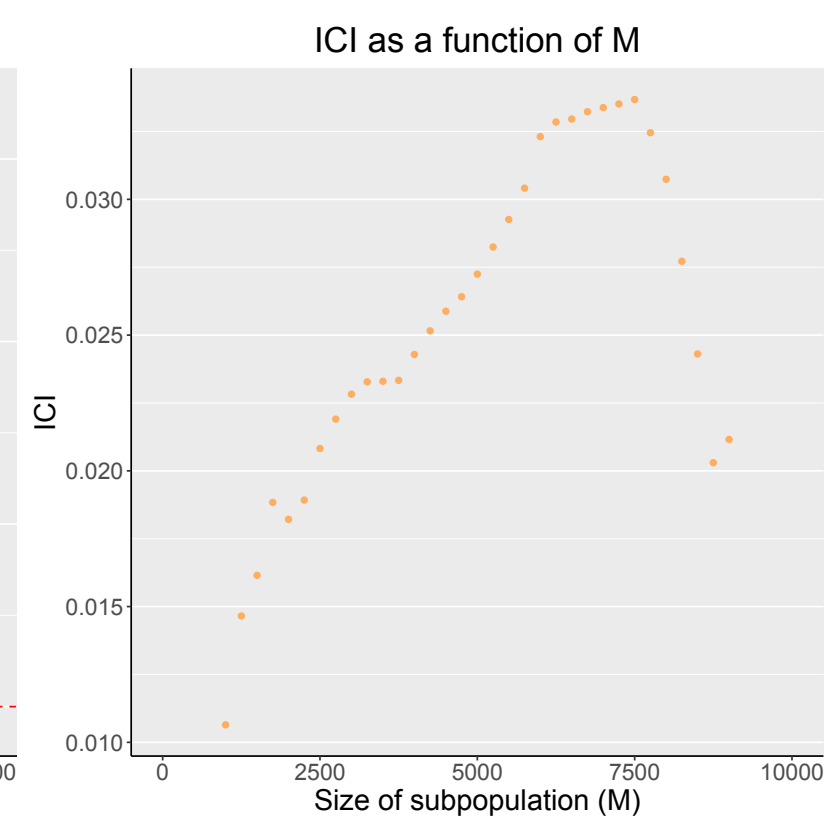

(f) ICI

Figure S5: Performance measures as a function of M when the outcome is simulated by a model which is linear, has 20 predictors associated with the outcome and results in a binary outcome with a prevalence of approximately 0.50.

S.1.6 Scenario 7: Non-Linear, 10 Association, Low Prevalence

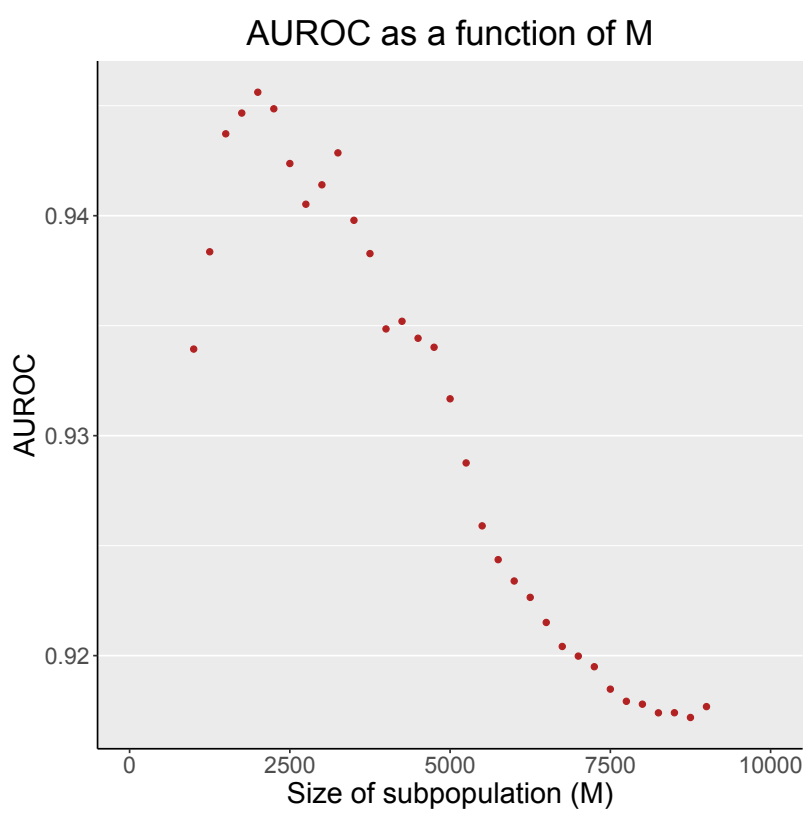

(a) AUROC

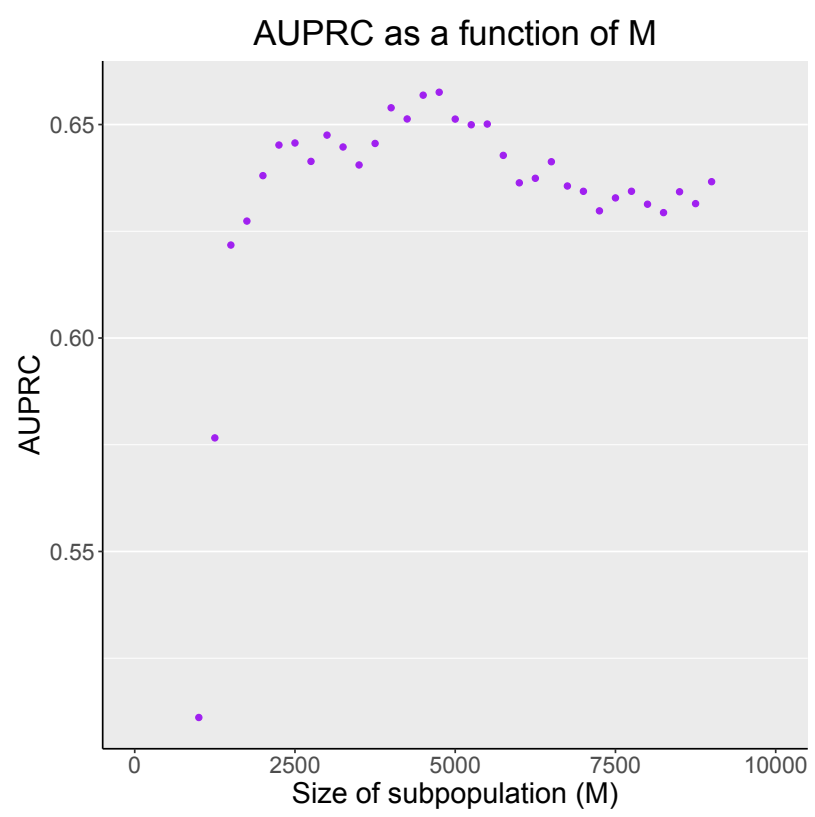

(b) AUPRC

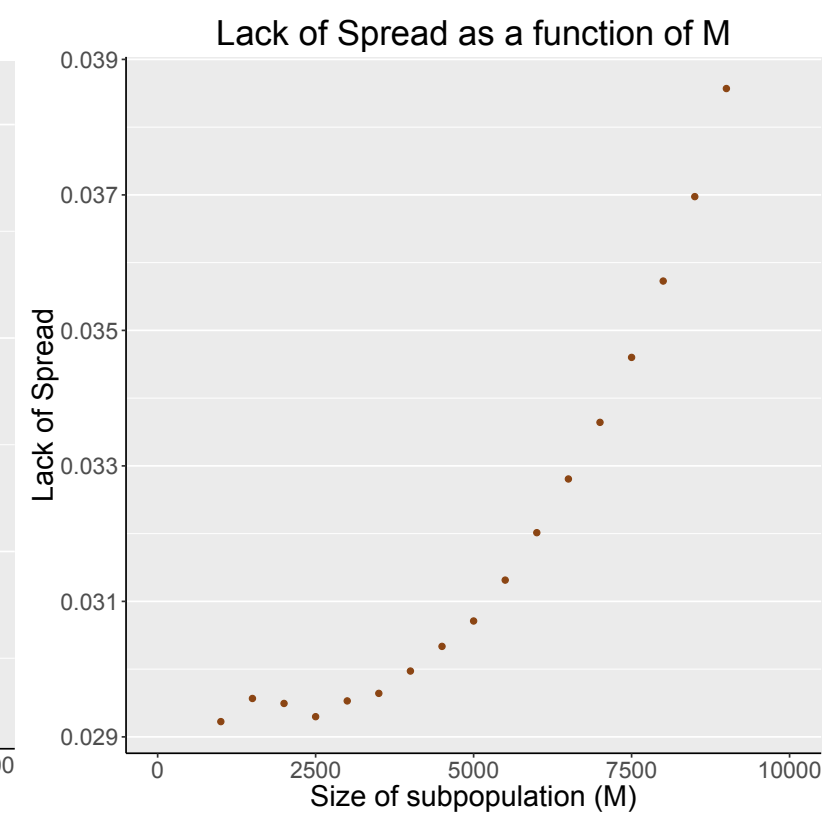

(c) Lack of Spread

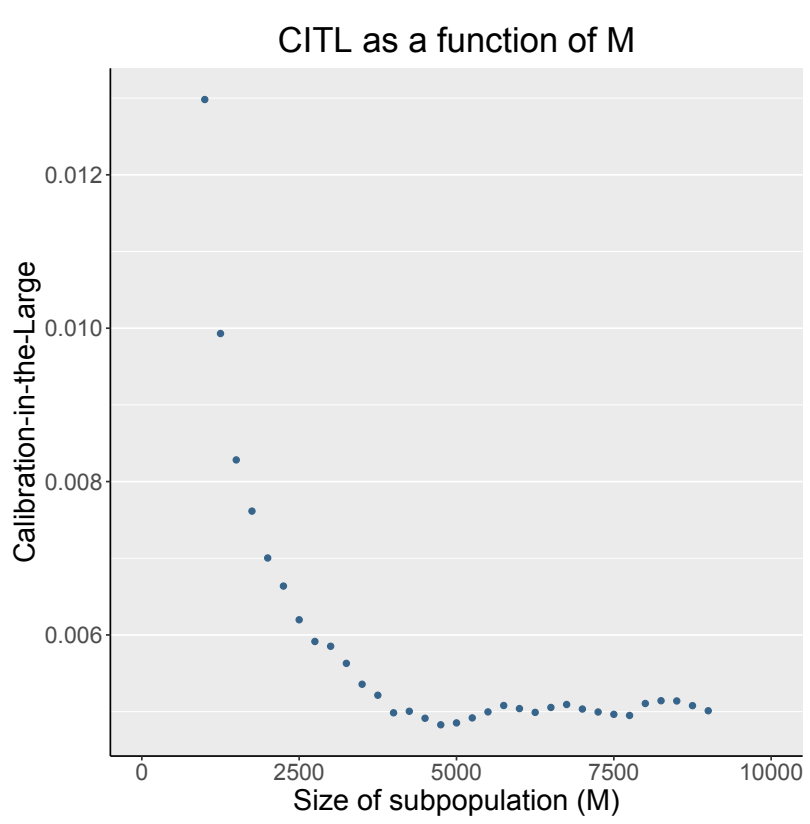

(d) CITL

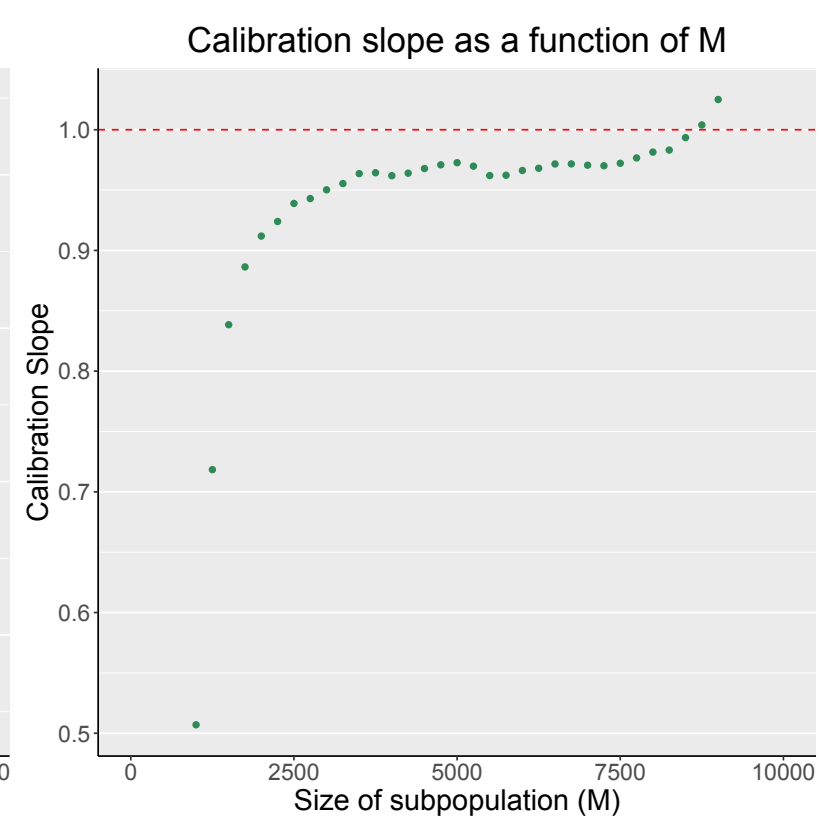

(e) Calibration Slope

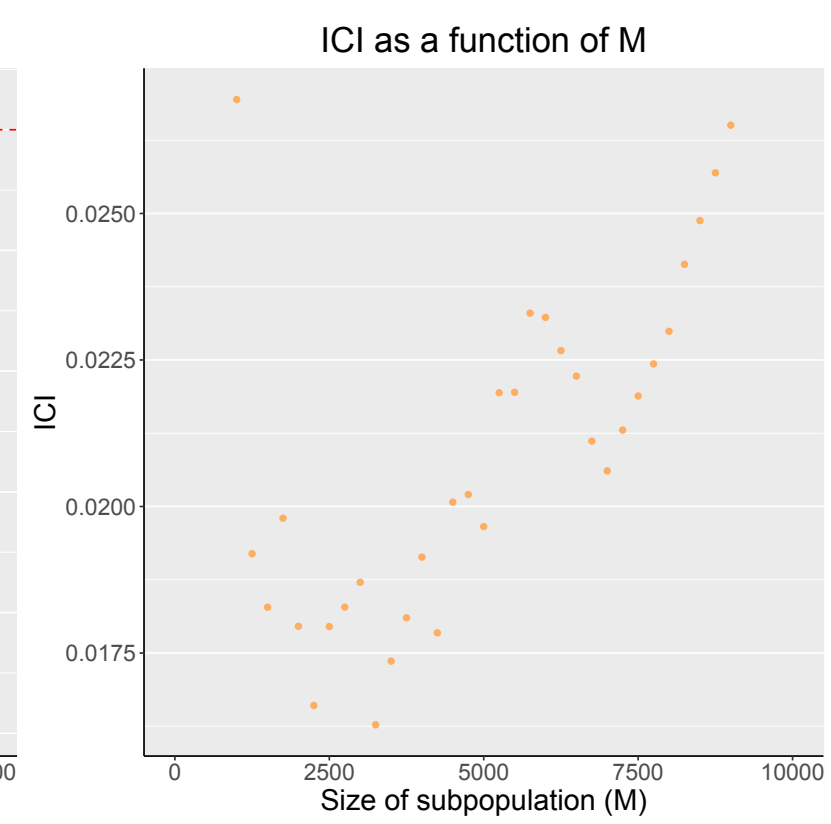

(f) ICI

Figure S6: Performance measures as a function of M when the outcome is simulated by a model which is non-linear, has 10 predictors associated with the outcome and results in a binary outcome with a prevalence of approximately 0.05.

S.1.7 Scenario 8: Non-Linear, 10 Association, Moderate Prevalence

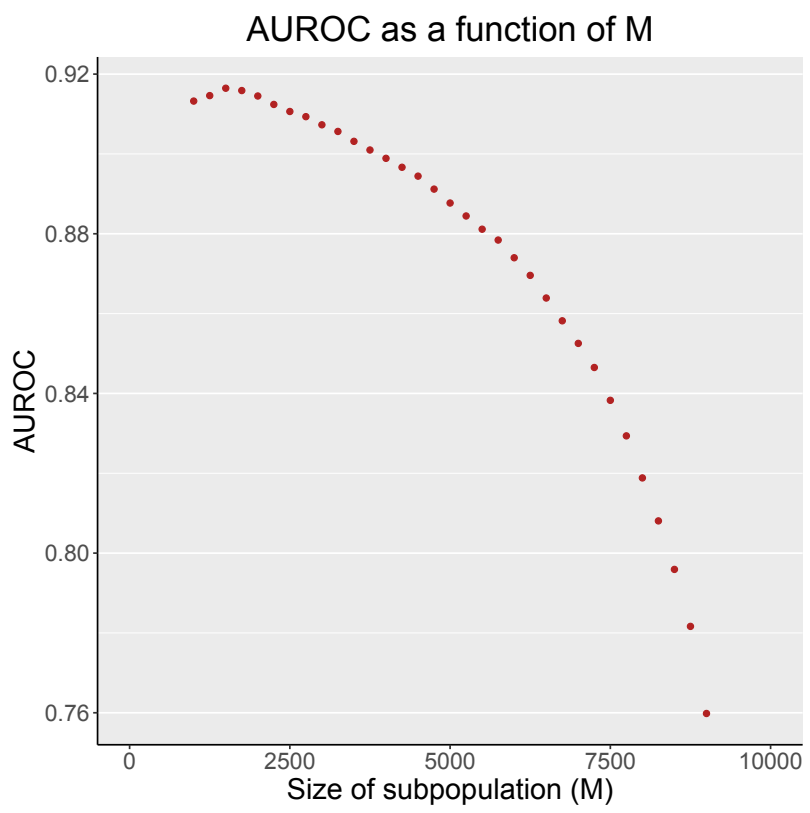

(a) AUROC

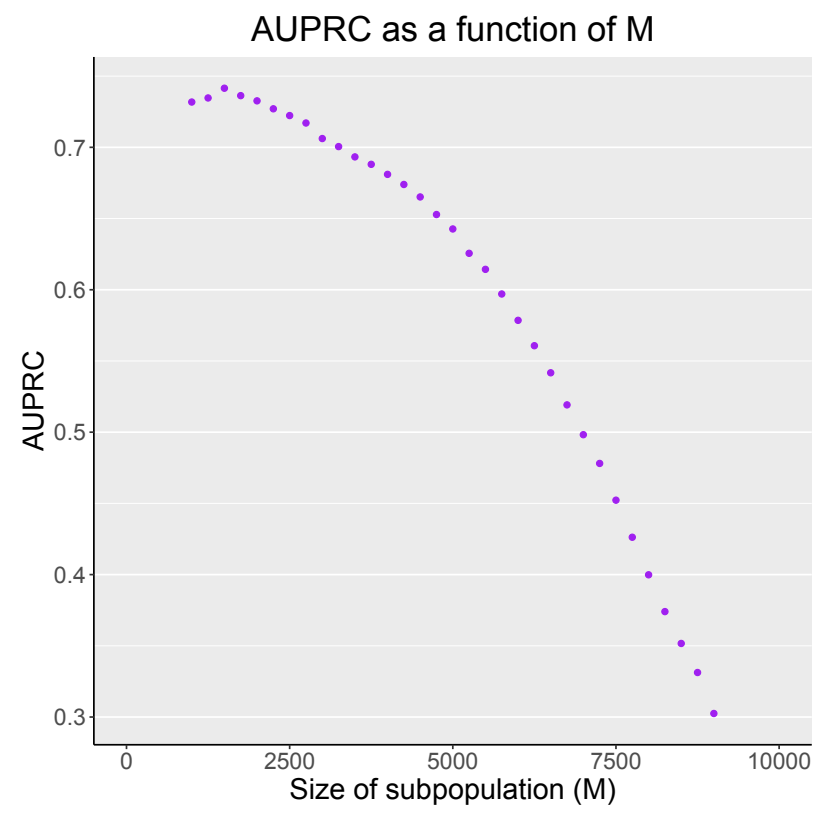

(b) AUPRC

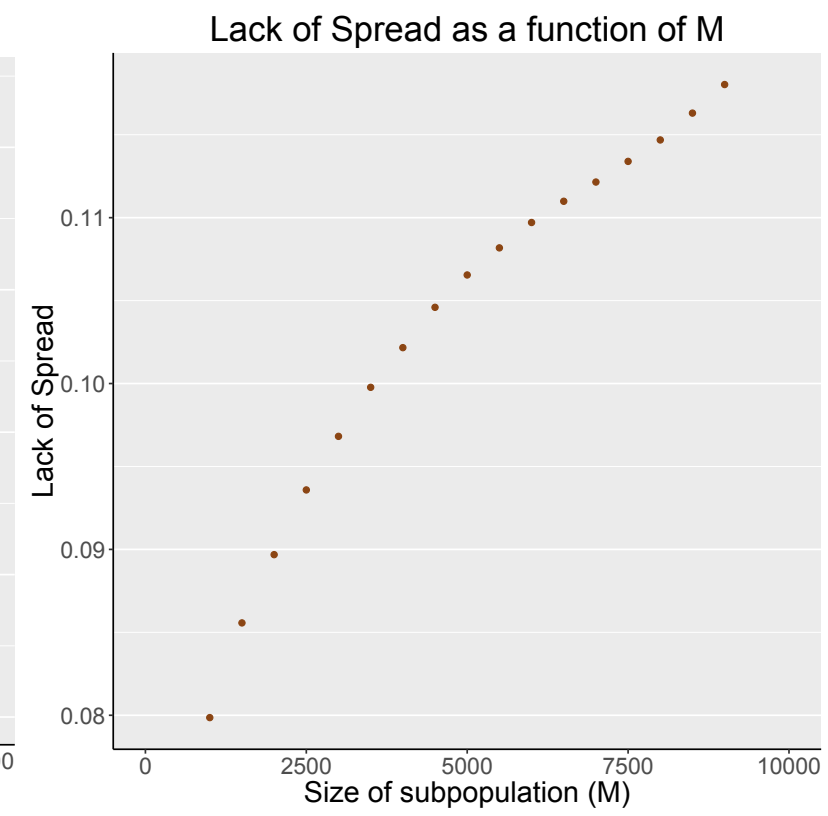

(c) Lack of Spread

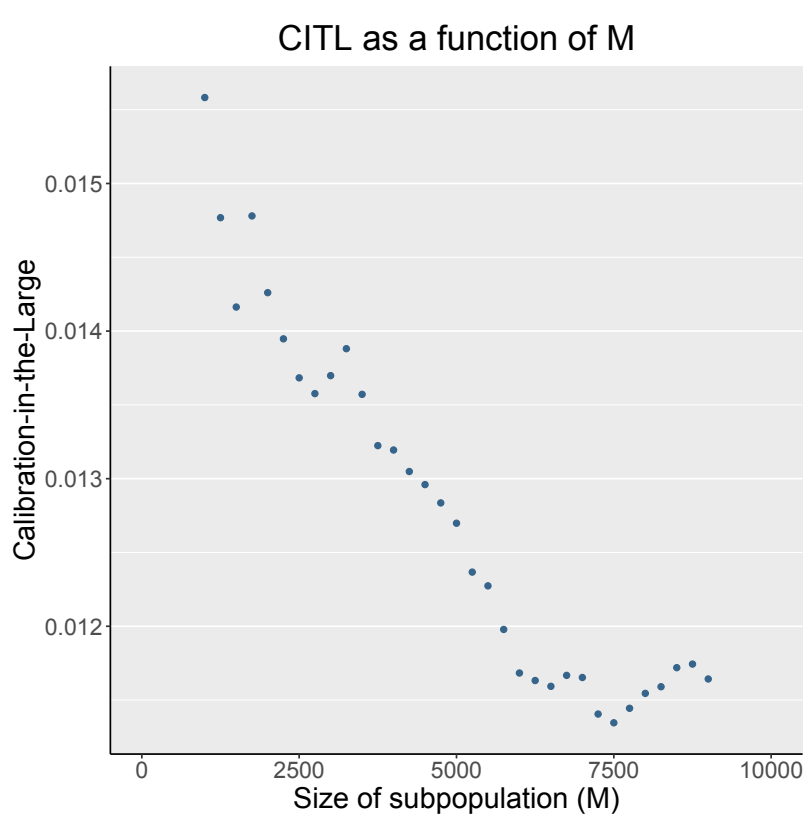

(d) CITL

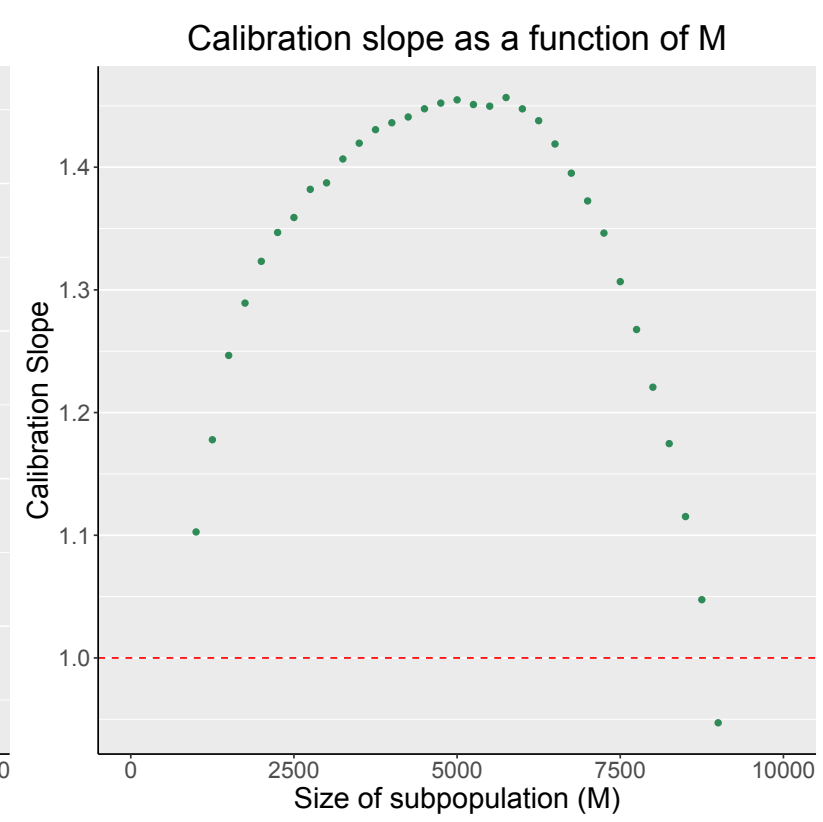

(e) Calibration Slope

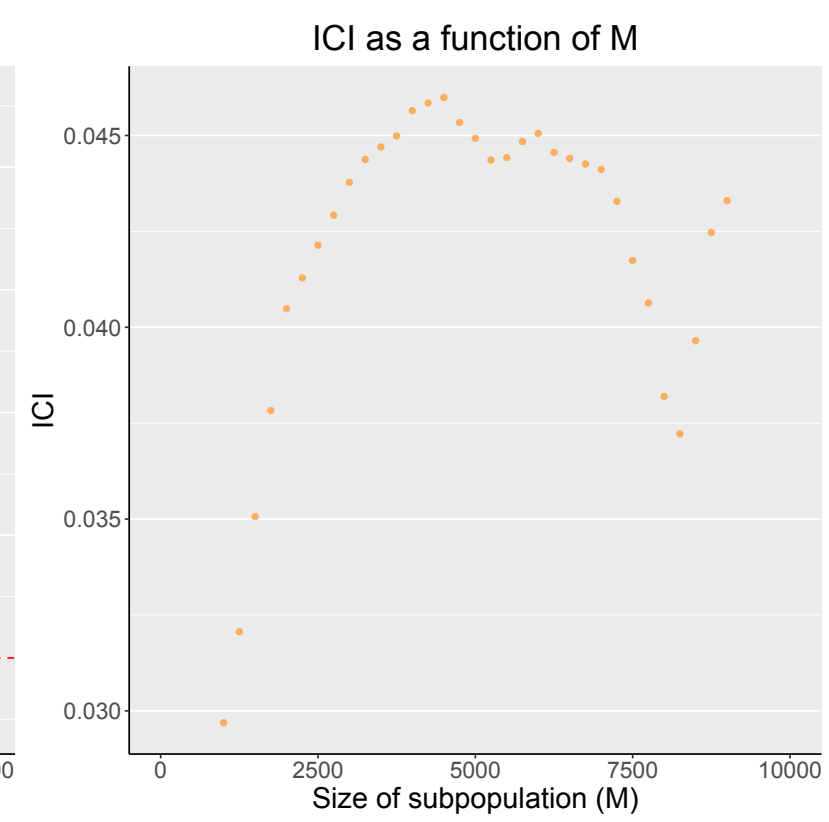

(f) ICI

Figure S7: Performance measures as a function of M when the outcome is simulated by a model which is non-linear, has 10 predictors associated with the outcome and results in a binary outcome with a prevalence of approximately 0.15.

S.1.8 Scenario 9: Non-Linear, 10 Association, Balanced Prevalence

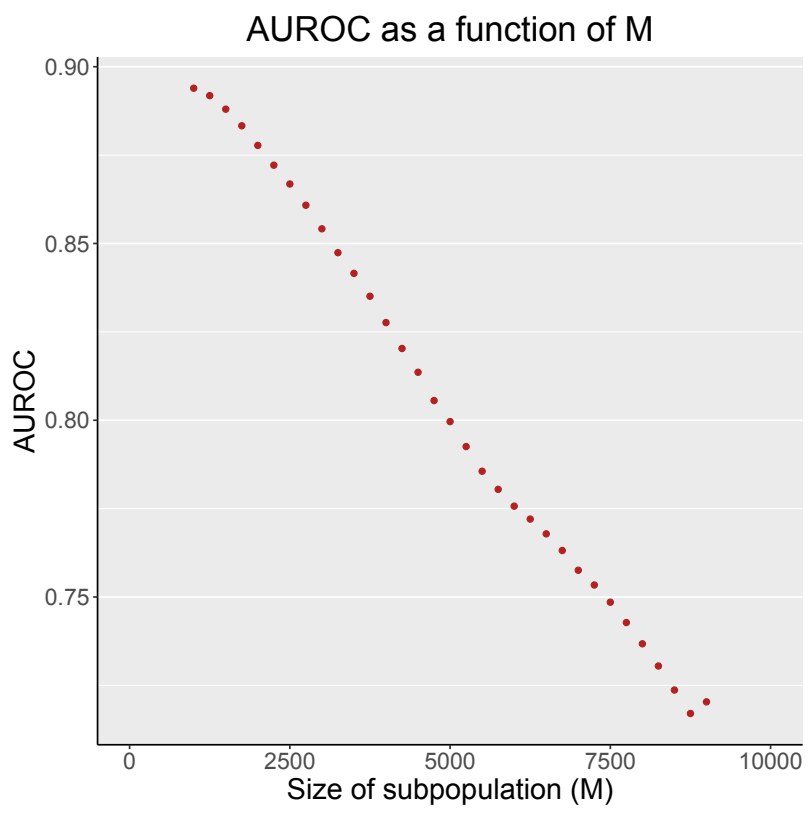

(a) AUROC

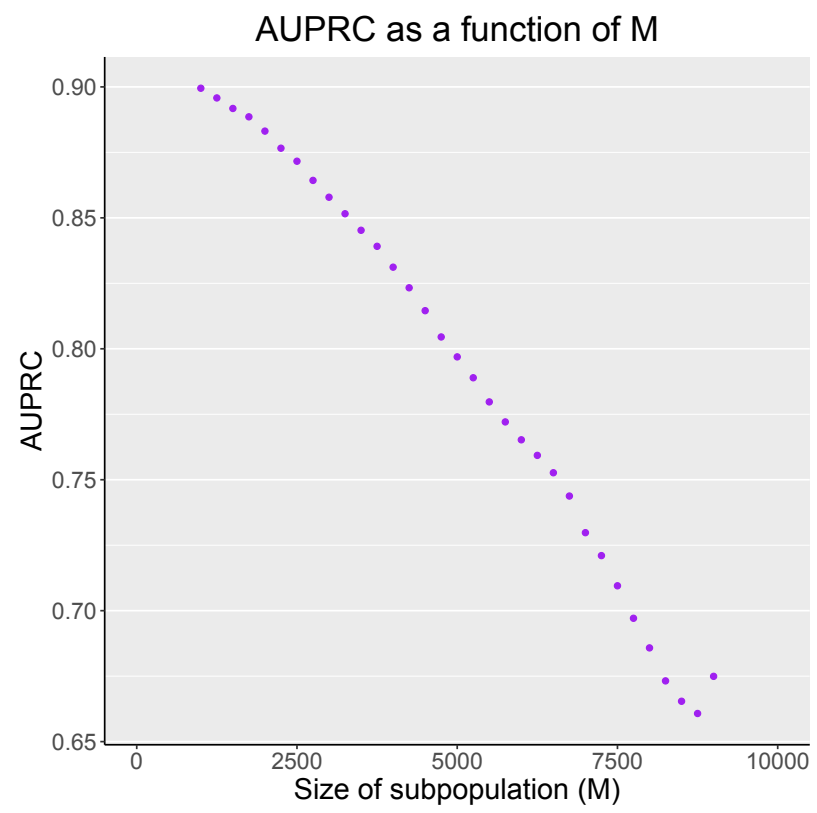

(b) AUPRC

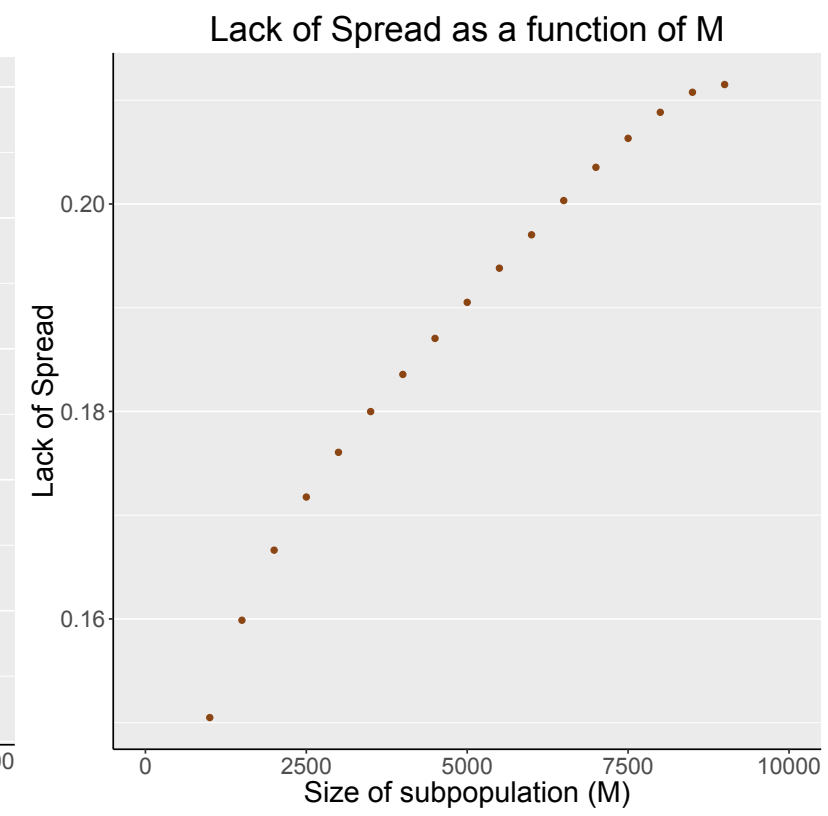

(c) Lack of Spread

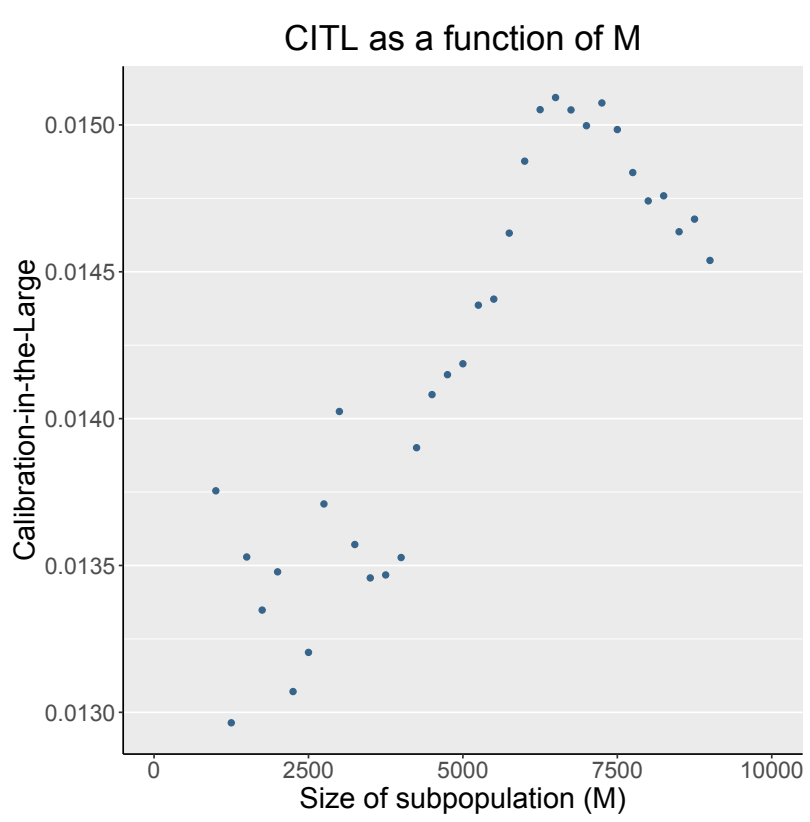

(d) CITL

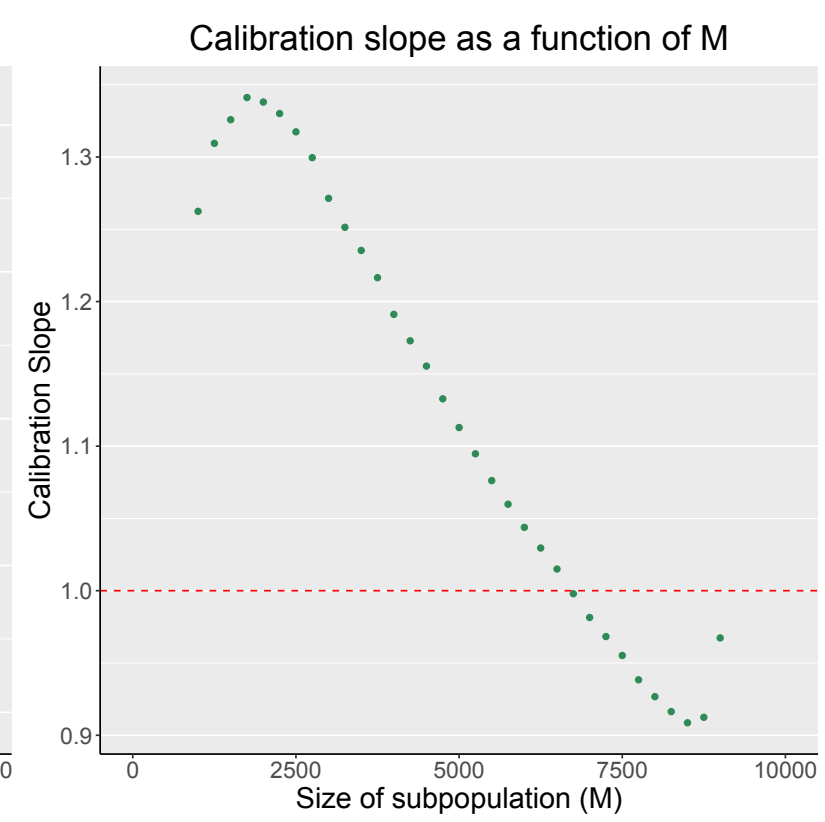

(e) Calibration Slope

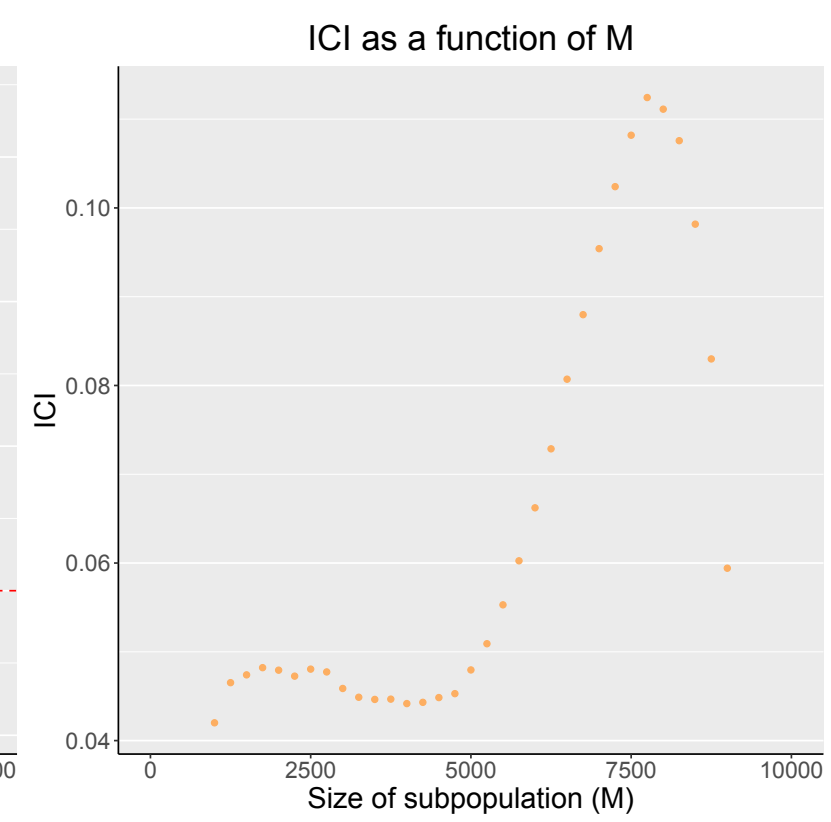

(f) ICI

Figure S8: Performance measures as a function of M when the outcome is simulated by a model which is non-linear, has 10 predictors associated with the outcome and results in a binary outcome with a prevalence of approximately 0.50.

S.1.9 Scenario 11: Non-Linear, 20 Association, Moderate Prevalence

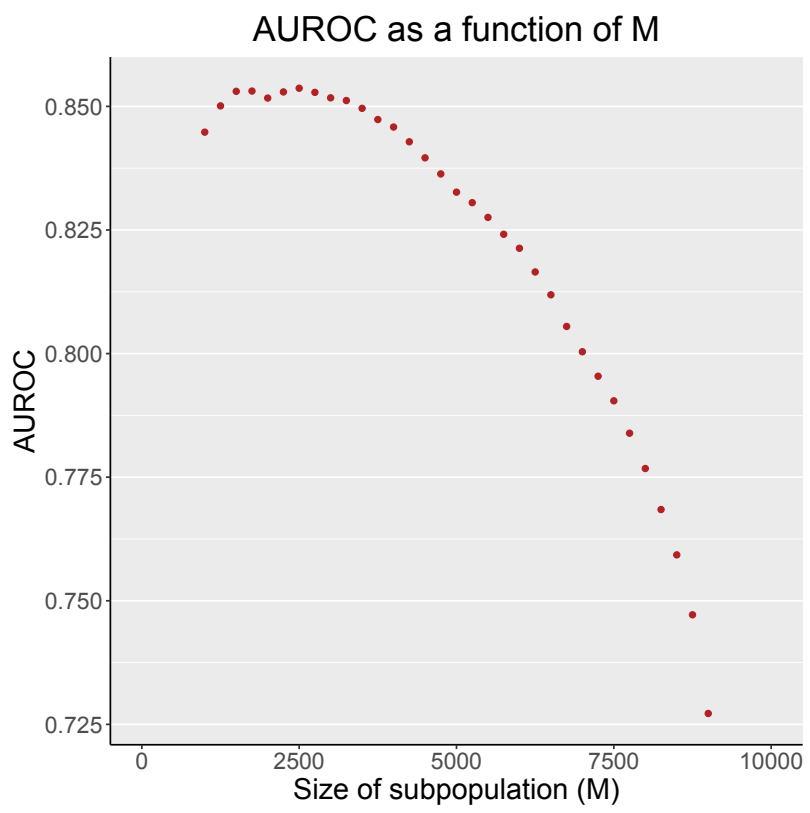

(a) AUROC

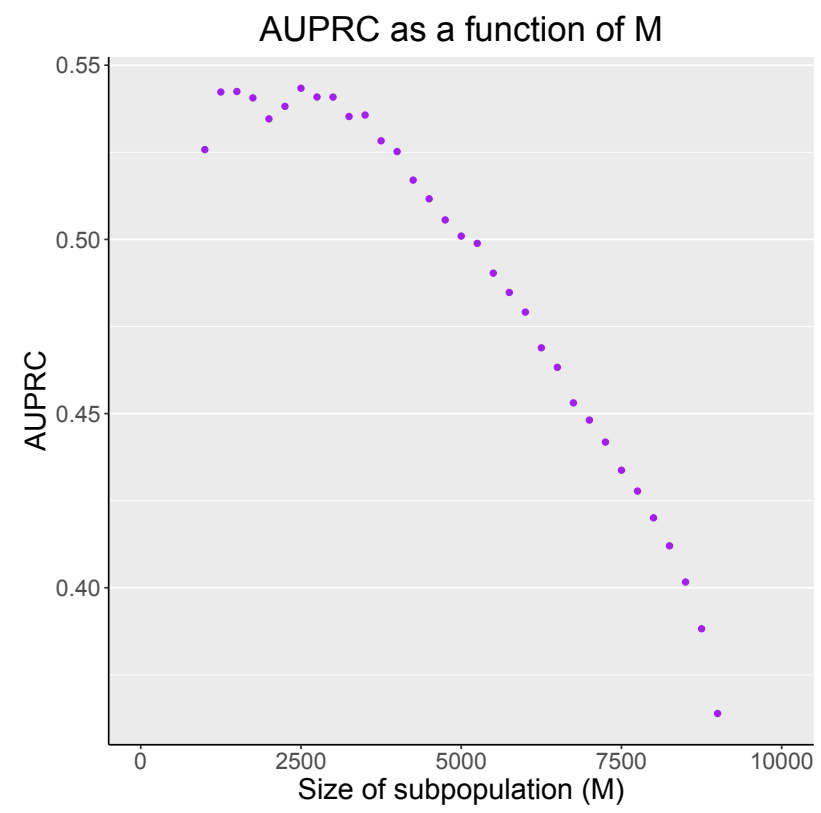

(b) AUPRC

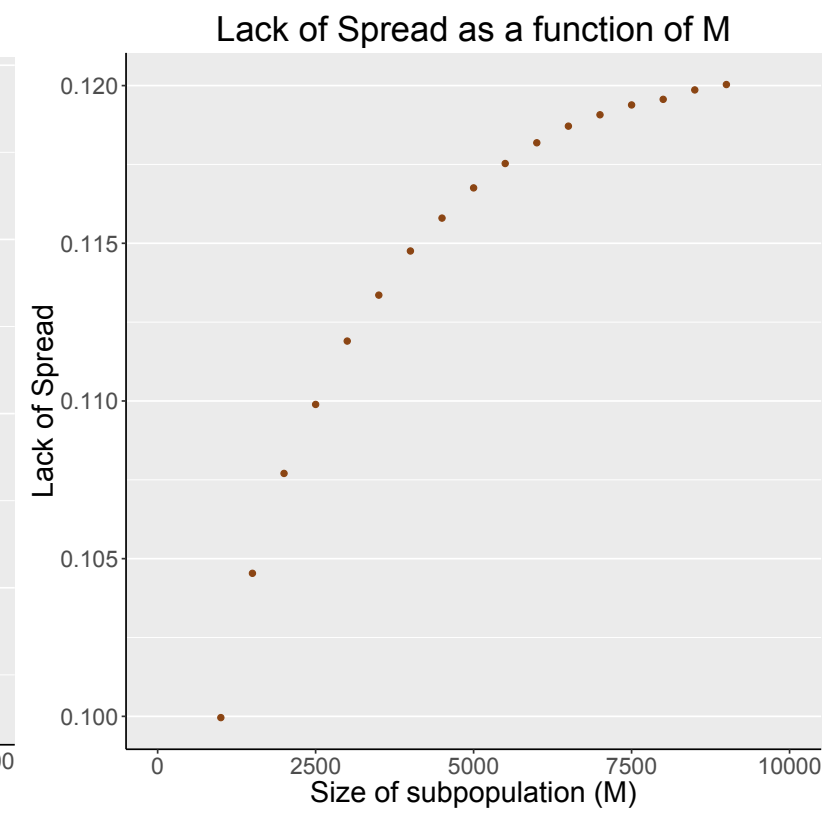

(c) Lack of Spread

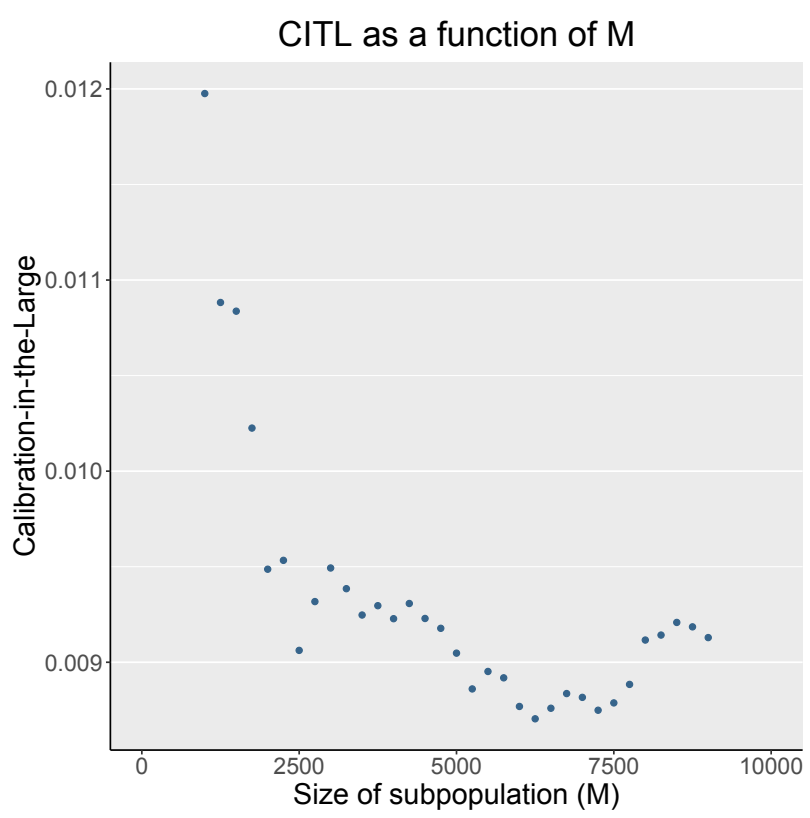

(d) CITL

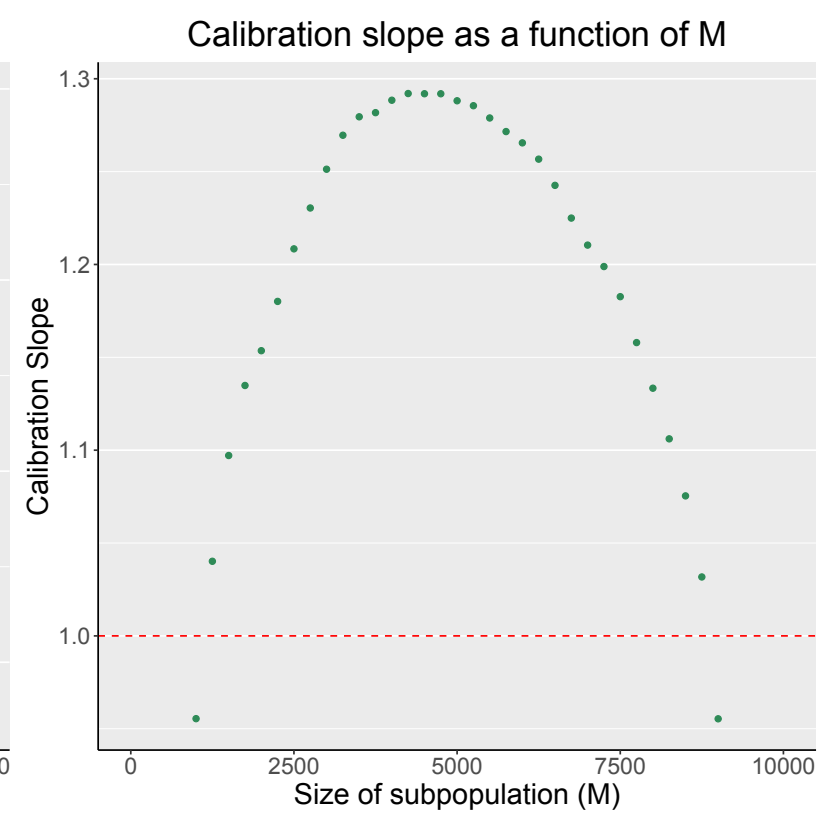

(e) Calibration Slope

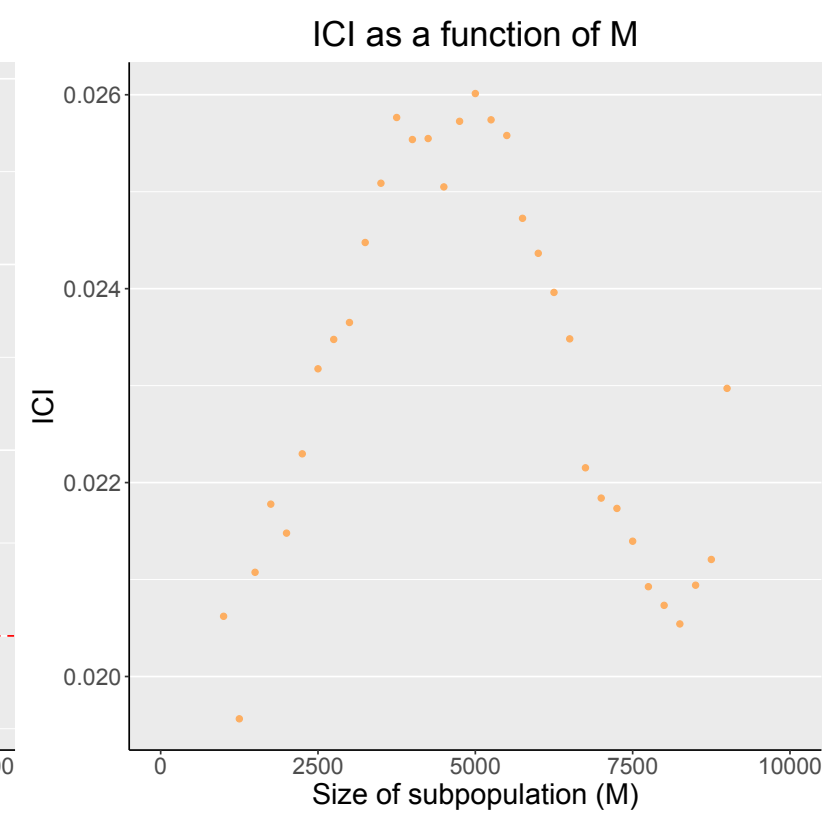

(f) ICI

Figure S9: Performance measures as a function of M when the outcome is simulated by a model which is non-linear, has 20 predictors associated with the outcome and results in a binary outcome with a prevalence of approximately 0.15.

S.1.10 Scenario 12: Non-Linear, 20 Association, Balanced Prevalence

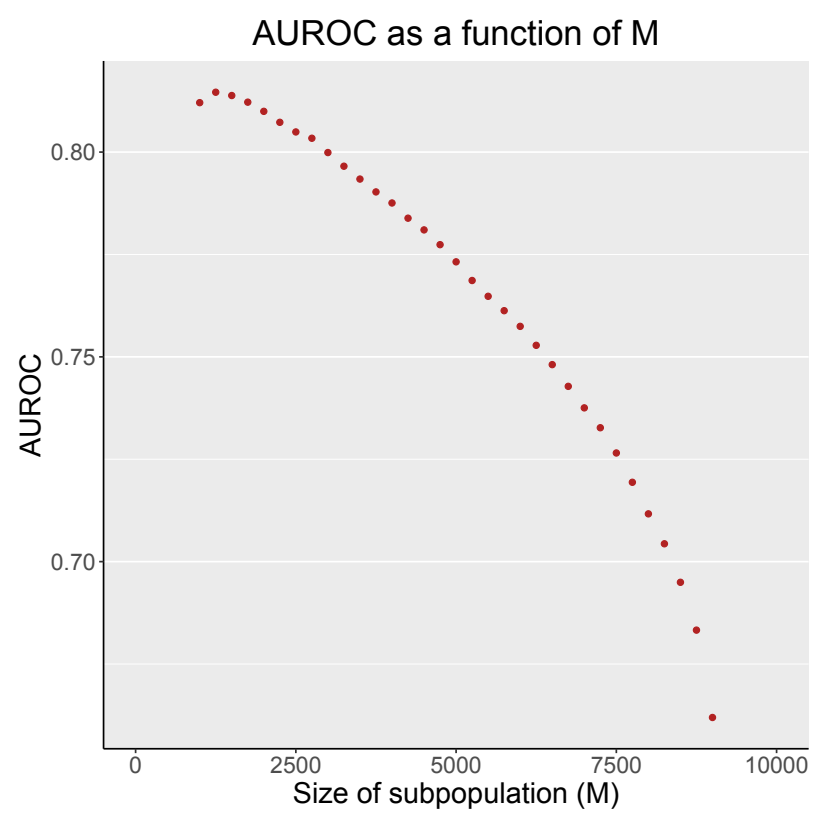

(a) AUROC

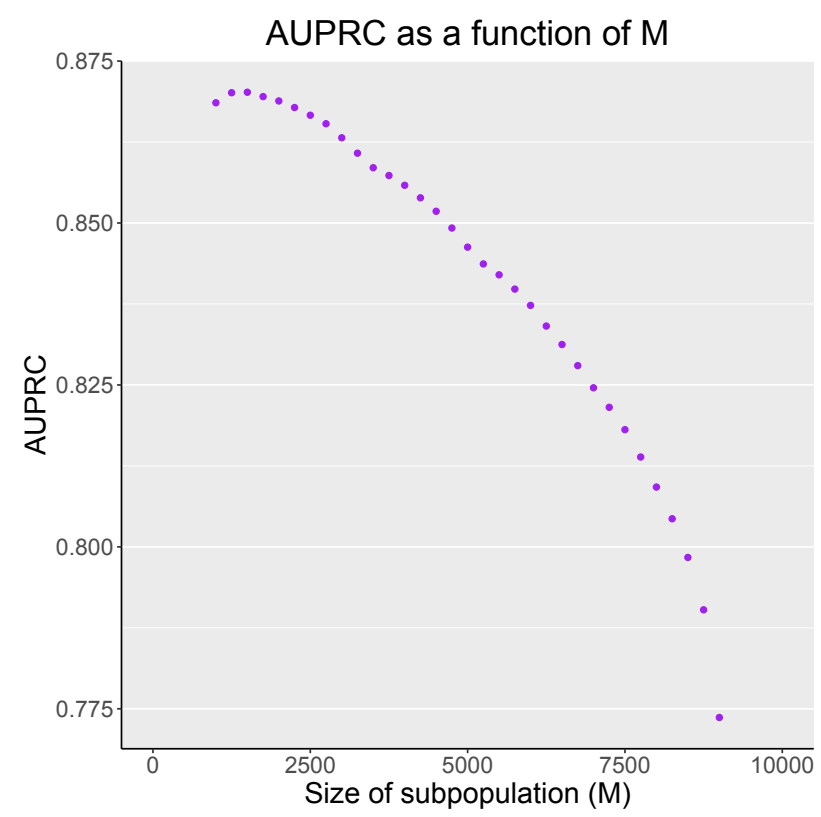

(b) AUPRC

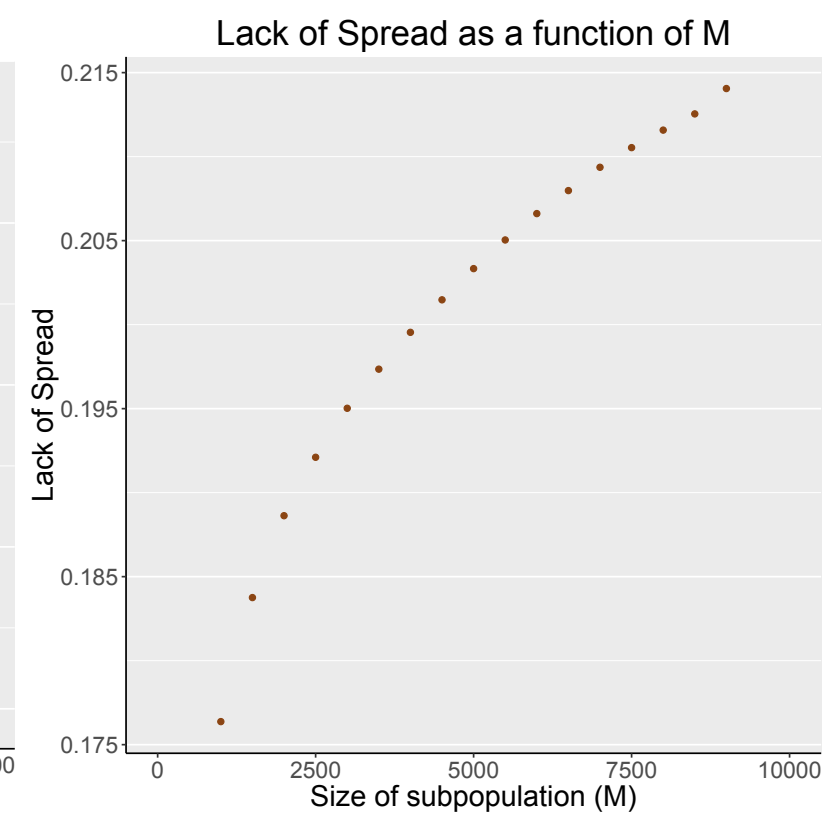

(c) Lack of Spread

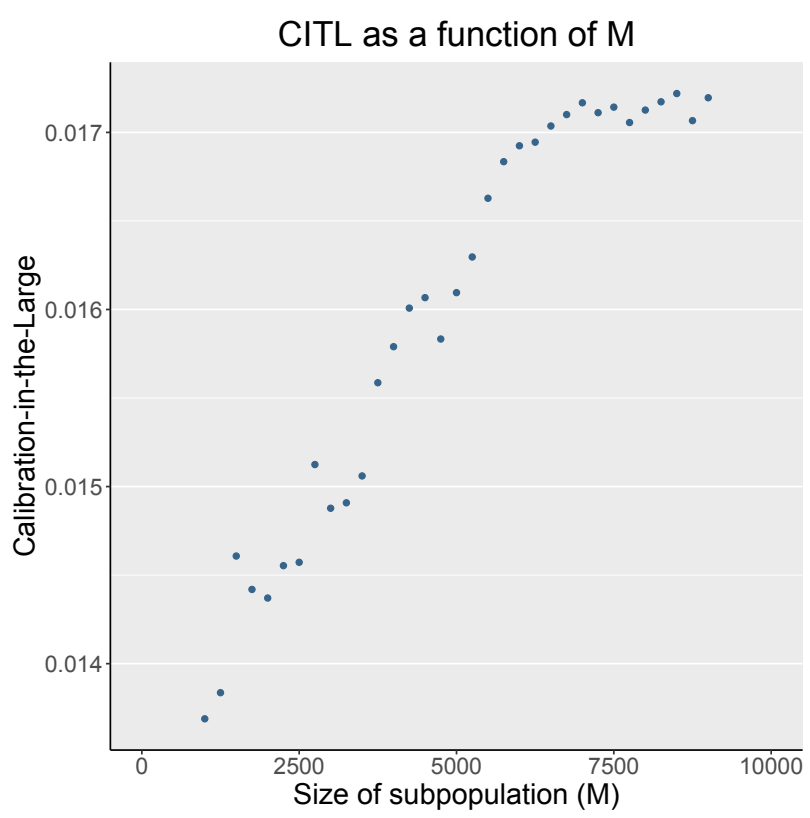

(d) CITL

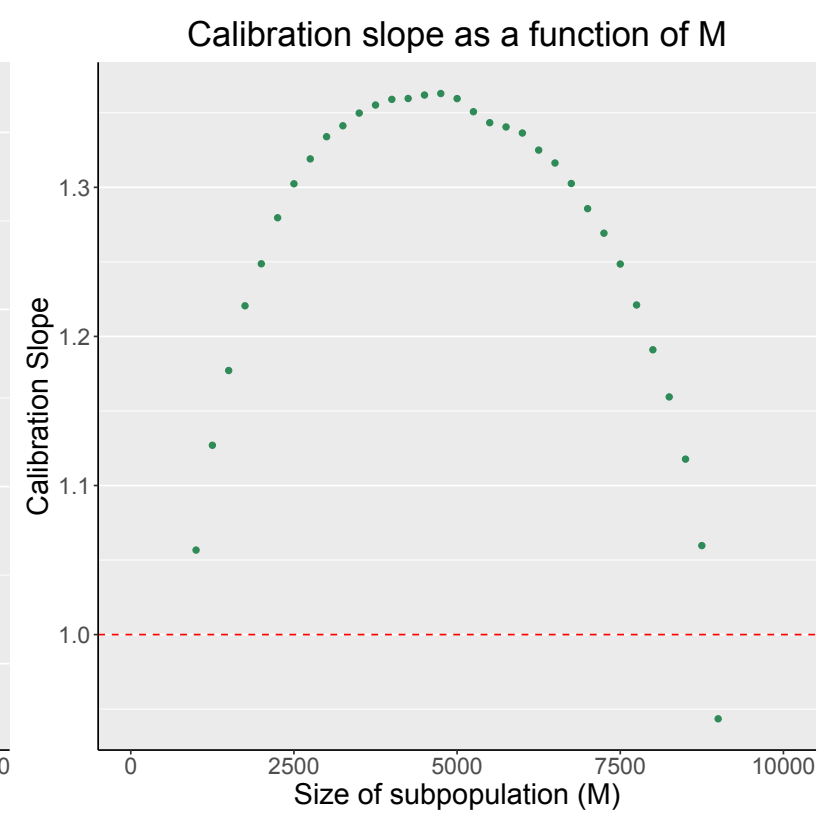

(e) Calibration Slope

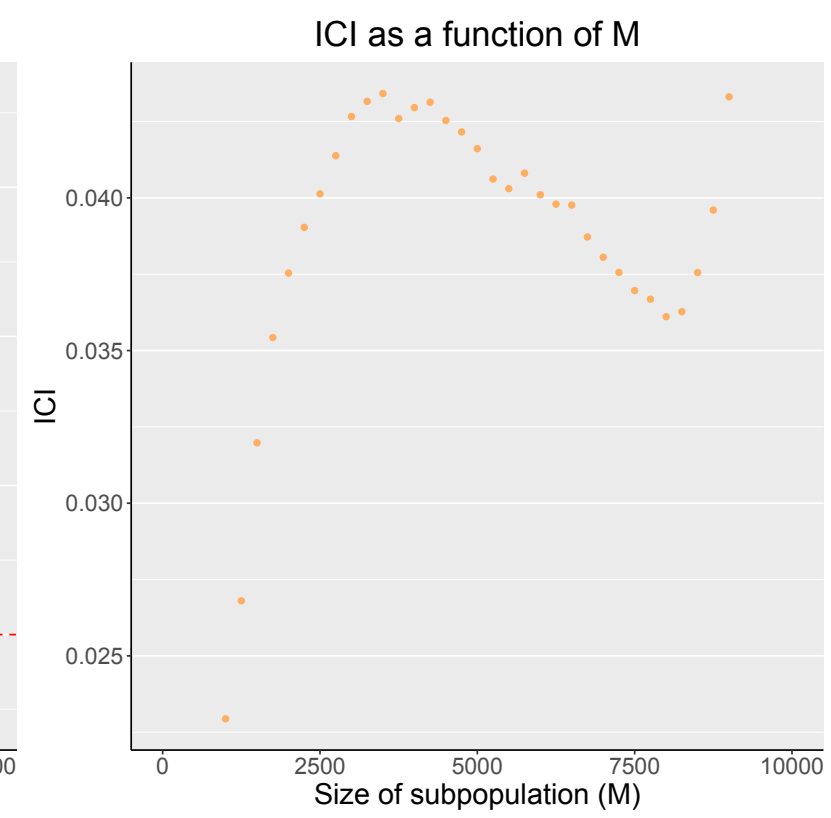

(f) ICI

Figure S10: Performance measures as a function of M when the outcome is simulated by a model which is non-linear, has 20 predictors associated with the outcome and results in a binary outcome with a prevalence of approximately 0.50.

S.2 Sample Size Investigation

We conducted a small simulation study to investigate the effect of sample size on the relationship between M and the various performance measures considered. We considered the scenario where the outcome was simulated using a linear true outcome model with 20 predictors associated with the outcome and a resulting prevalence that is approximately 0.15. We considered three different sample sizes: $N = \{5000, 10000, 20000\}$, and we fit PPMs using logistic regression. We display the relationships between M and six performance measures (AUROC, AUPRC, lack of spread, CITL, calibration slope, ICI) in Figures S11, S12, and S13. In all cases, M and discrimination have a negatively proportional relationship: as M increases, the discriminative ability of the PPMs decrease. Further, we have a generally quadtratic relationship between M and all levels of calibration for all sample sizes considered as well. In Figure S11, where $N = 5000$, there is some instability for small values of M where the calibration is poor, but if we exclude those outliers, the relationship is quadratic.

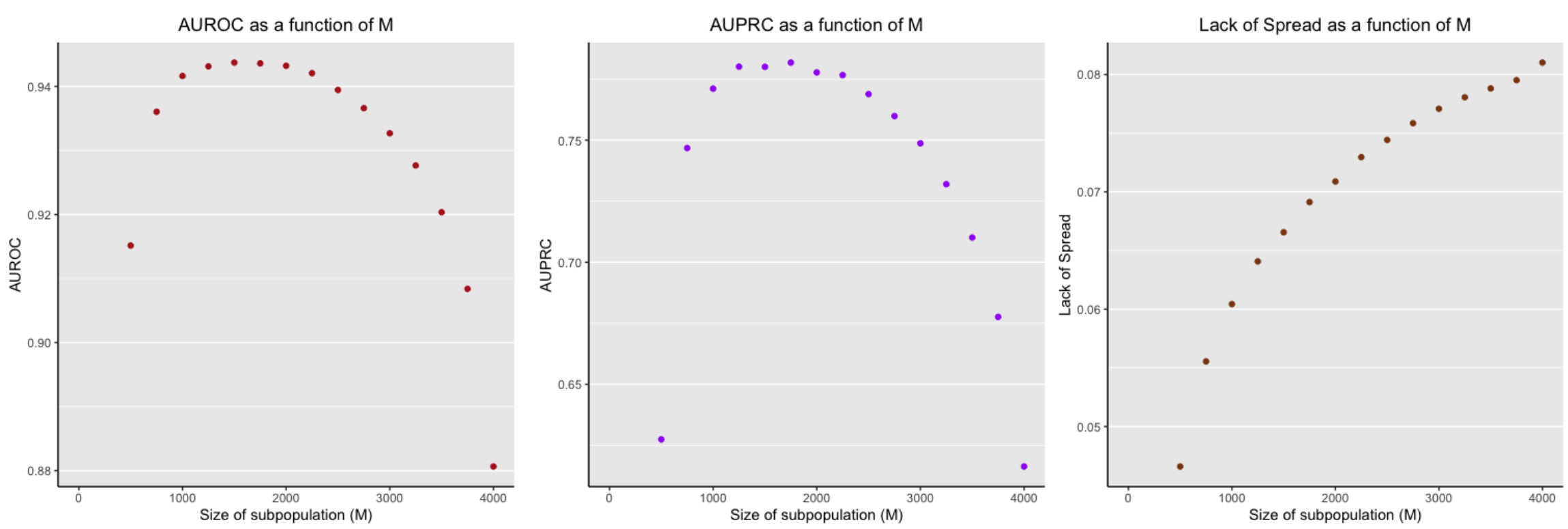

(a) AUROC (b) AUPRC (c) Lack of Spread

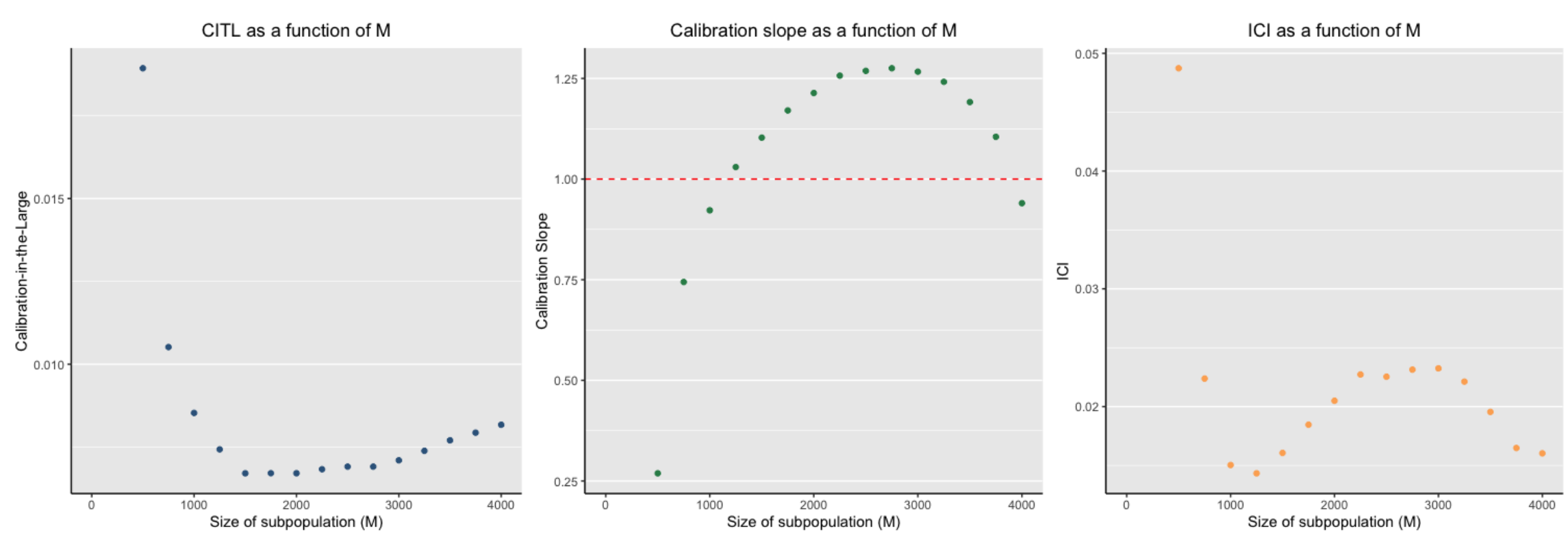

(d) CITL (e) Calibration Slope (f) ICI

Figure S11: Performance measures as a function of M for Scenario A. In this simulation study, $N = 5000$ to assess the effect of sample size on the relationship between M and the various performance measures considered.

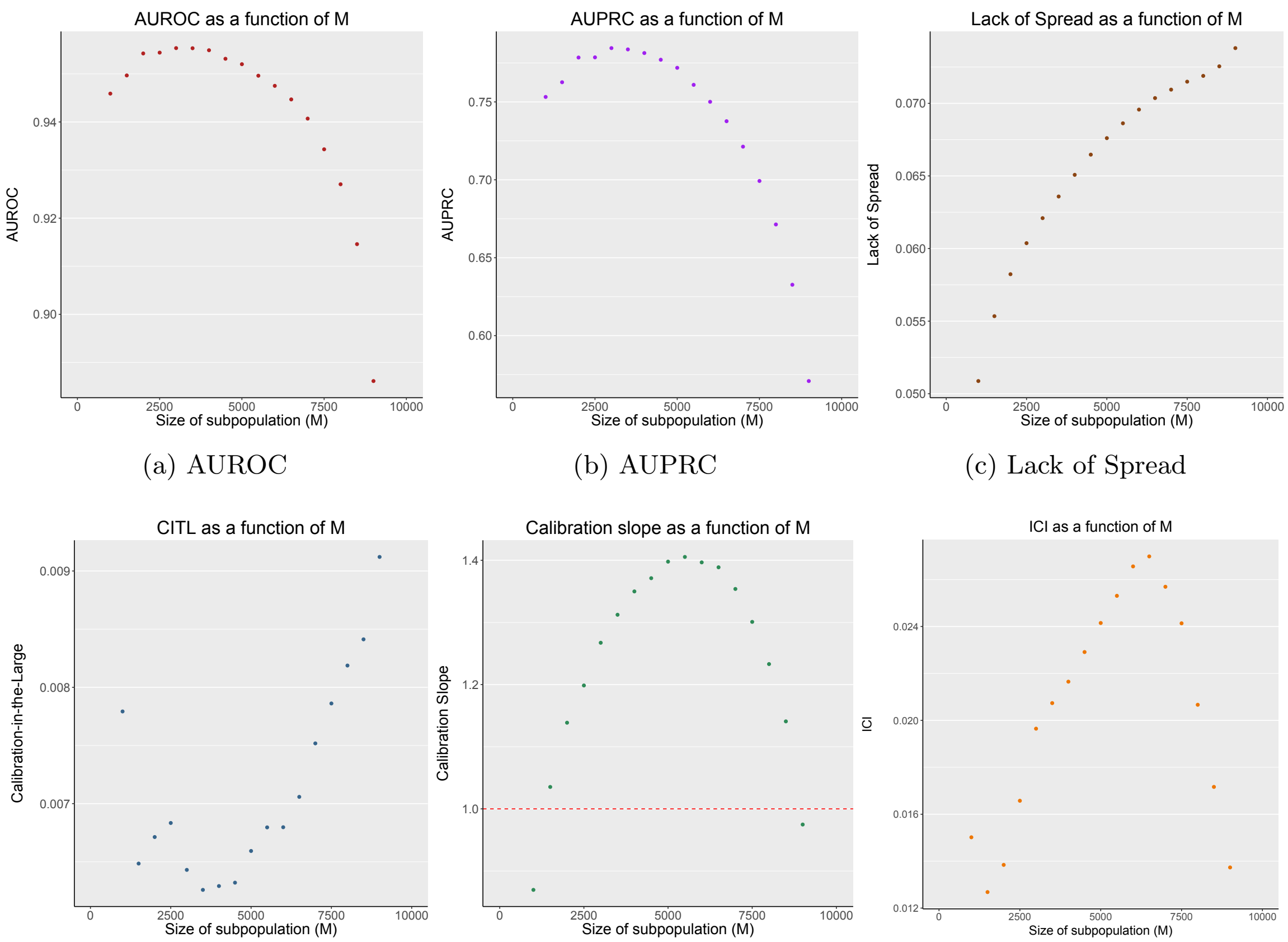

(a) AUROC (b) AUPRC (c) Lack of Spread

(d) CITL (e) Calibration Slope (f) ICI

Figure S12: Performance measures as a function of M for Scenario A. In this simulation study, $N = 10000$ to assess the effect of sample size on the relationship between M and the various performance measures considered.

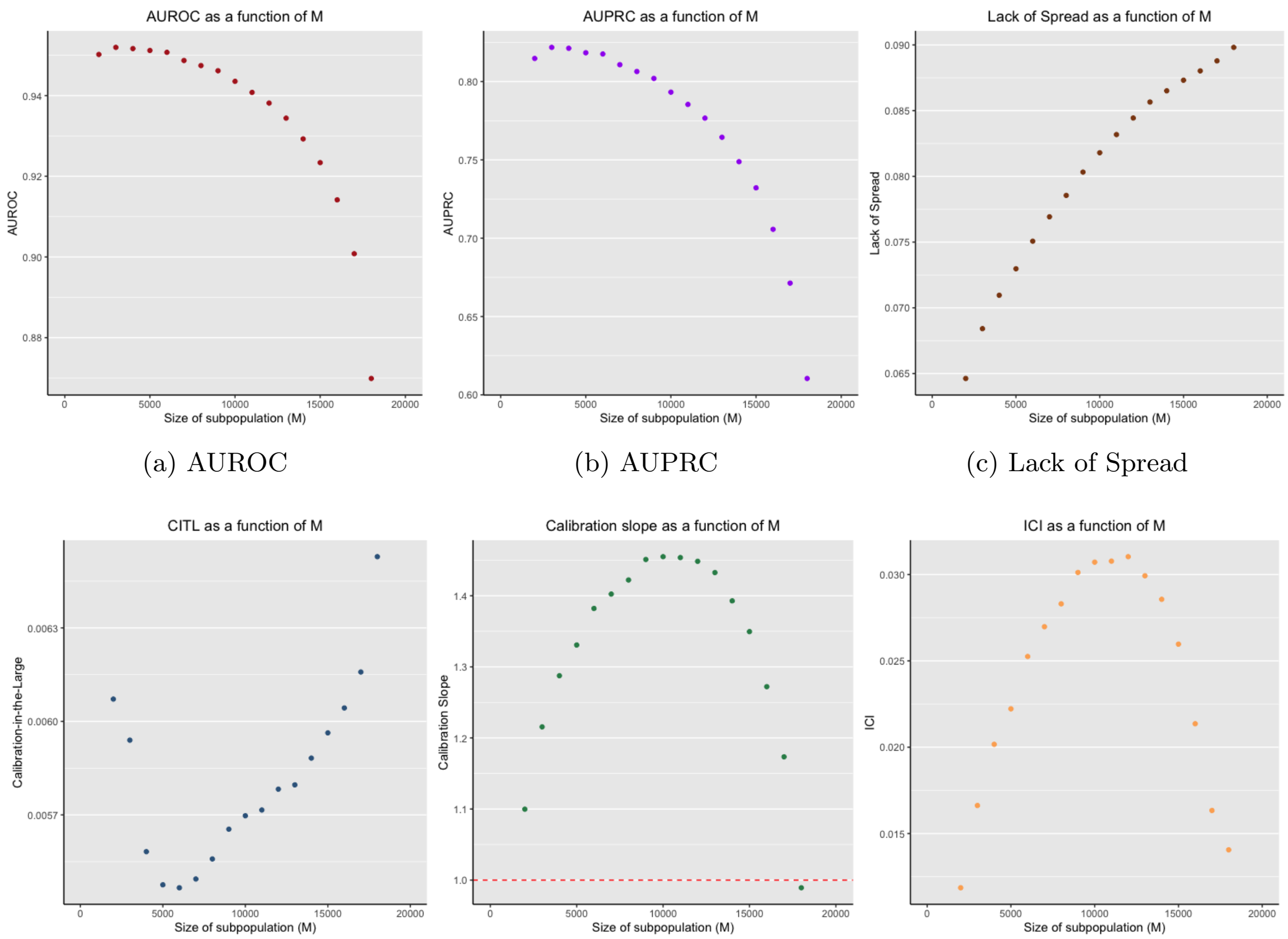

(a) AUROC (b) AUPRC (c) Lack of Spread

(d) CITL (e) Calibration Slope (f) ICI

Figure S13: Performance measures as a function of M for Scenario A. In this simulation study, $N = 20,000$ to assess the effect of sample size on the relationship between M and the various performance measures considered.

S.3 eICU Cohort Selection

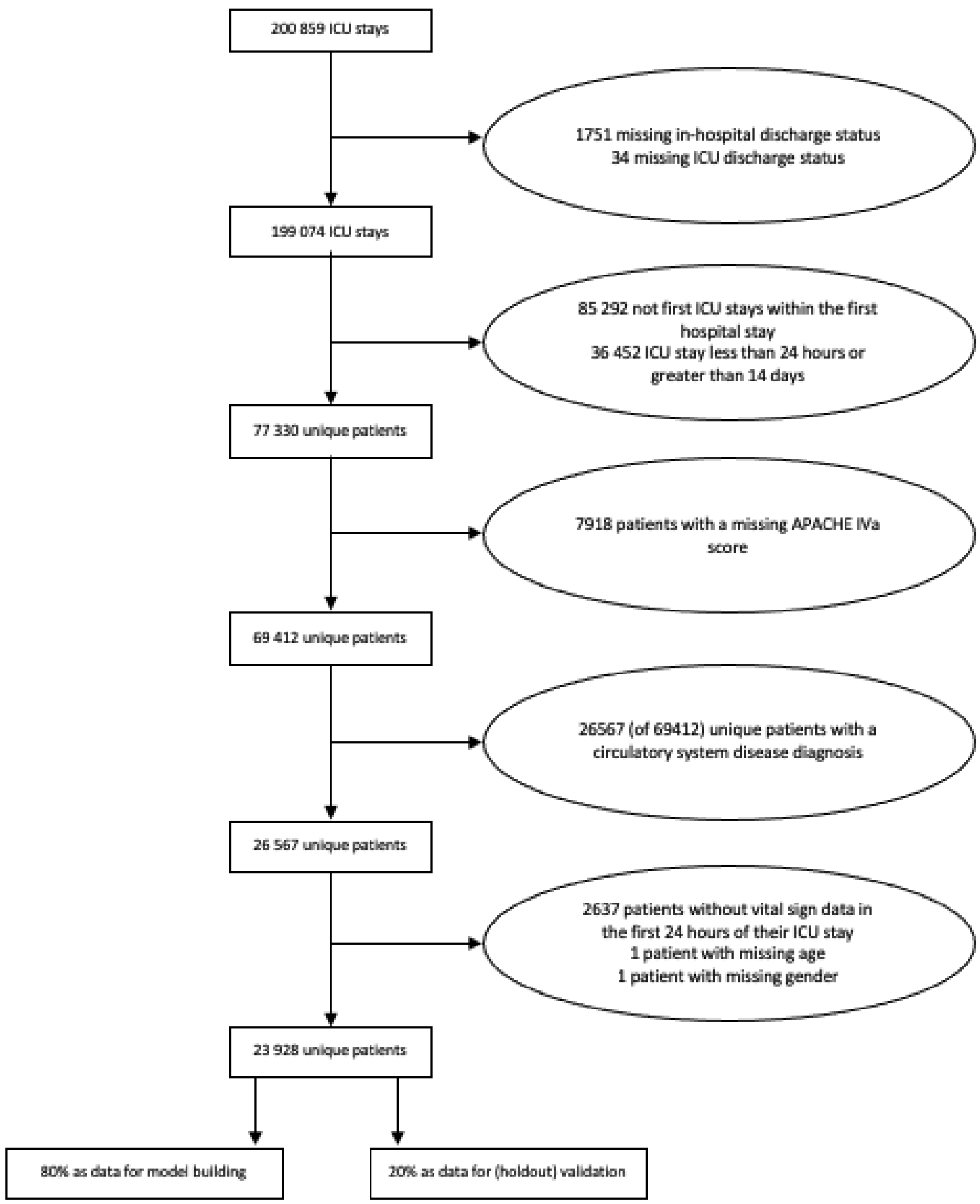

Figure S14: Flow chart of cohort selection from the eICU database.
